# *In situ* morphometric survey elucidates the evolutionary systematics of the Eurasian *Himantoglossum* clade (Orchidaceae: Orchidinae)

**DOI:** 10.7717/peerj.2893

**Published:** 2017-01-31

**Authors:** Richard M. Bateman, Attila Molnár V., Gábor Sramkó

**Affiliations:** 1 Royal Botanic Gardens Kew, Richmond, Surrey, United Kingdom; 2 Department of Botany, University of Debrecen, Debrecen, Hungary; 3 MTA-DE “Lendület” Evolutionary Phylogenomics Research Group, Debrecen, Hungary

**Keywords:** *Barlia*, *Comperia*, Disparity, Functional constraints, Heterochrony, *Himantoglossum*, Hybridisation, Migration, Molecular phylogeny, Morphometrics, Orchid, Parallelism, Speciation, Systematics

## Abstract

**Background and Aims:**

The charismatic *Himantoglossum s.l.* clade of Eurasian orchids contains an unusually large proportion of taxa that are of controversial circumscriptions and considerable conservation concern. Whereas our previously published study addressed the molecular phylogenetics and phylogeography of every named taxon within the clade, here we use detailed morphometric data obtained from the same populations to compare genotypes with associated phenotypes, in order to better explore taxonomic circumscription and character evolution within the clade.

**Methods:**

Between one and 12 plants found in 25 populations that encompassed the entire distribution of the *Himantoglossum s.l.* clade were measured *in situ* for 51 morphological characters. Results for 45 of those characters were subjected to detailed multivariate and univariate analyses.

**Key Results:**

Multivariate analyses readily separate subgenus *Barlia* and subgenus *Comperia* from subgenus *Himantoglossum*, and also the early-divergent *H. formosum* from the less divergent remainder of subgenus *Himantoglossum*. The sequence of divergence of these four lineages is confidently resolved. Our experimental approach to morphometric character analysis demonstrates clearly that phenotypic evolution within *Himantoglossum* is unusually multi-dimensional.

**Conclusions:**

Degrees of divergence between taxa shown by morphological analyses approximate those previously shown using molecular analyses. *Himantoglossum s.l*. is readily divisible into three subgenera. The three sections of subgenus *Himantoglossum*—*hircinum*, *caprinum* and *formosum—*are arrayed from west to east with only limited geographical overlap. At this taxonomic level, their juxtaposition combines with conflict between contrasting datasets to complicate attempts to distinguish between clinal variation and the discontinuities that by definition separate *bona fide* species. All taxa achieve allogamy via food deceit and have only weak pollinator specificity. Artificial crossing demonstrates that intrinsic sterility barriers are weak. Although we have found evidence of gene flow among and within the three sections of subgenus *Himantoglossum*, reports of natural hybrids are surprisingly rare, probably because putative parents are sufficiently similar to questionably warrant the status of species. Phenological separation and increased xeromorphy characterise the origin of subgenus *Barlia*. Several individual morphological characters show evidence of parallel acquisition, and loss of features is especially frequent in floral markings among members of section *caprinum*. Detailed patterns of gain and loss demonstrate that several different categories of flower markings are inherited independently. Along with the dimensions of labellar lobes, these pigmentation characters have been over-emphasised in previous taxonomic treatments. Increased plant vigour was a crucial element of the origin of the genus, but vegetative characters underwent remarkably little subsequent evolution. Attempts to reconstruct hypothetical ancestors at internal nodes of the phylogeny are weakened by (a) uncertain placement of *Steveniella* as sister to *Himantoglossum s.l.* and (b) uncertain relationships among subtly different putative species within section *caprinum*. Nonetheless, heterochronic/allometric trends, ultimately limited by functional constraints, clearly dictate transitions between contrasting flower sizes and complex labellum shapes.

## Introduction

### Background to the genus

**Figure 1:**
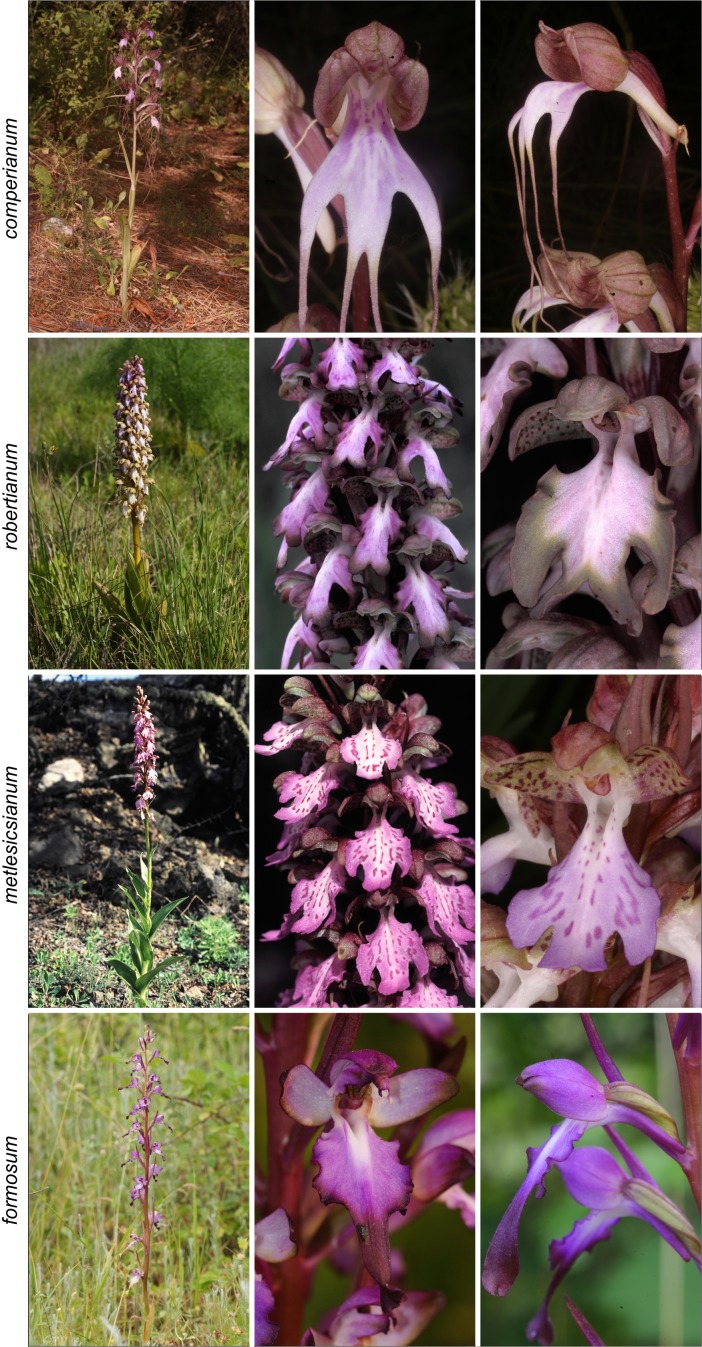
Typical flowers of taxa of *Himantoglossum* analysed in the present study, 1: *H. comperianum*, *H. robertianum*, *H. metlesicsianum*, and *H. formosum.* Images: Attila Molnár V.

**Figure 2:**
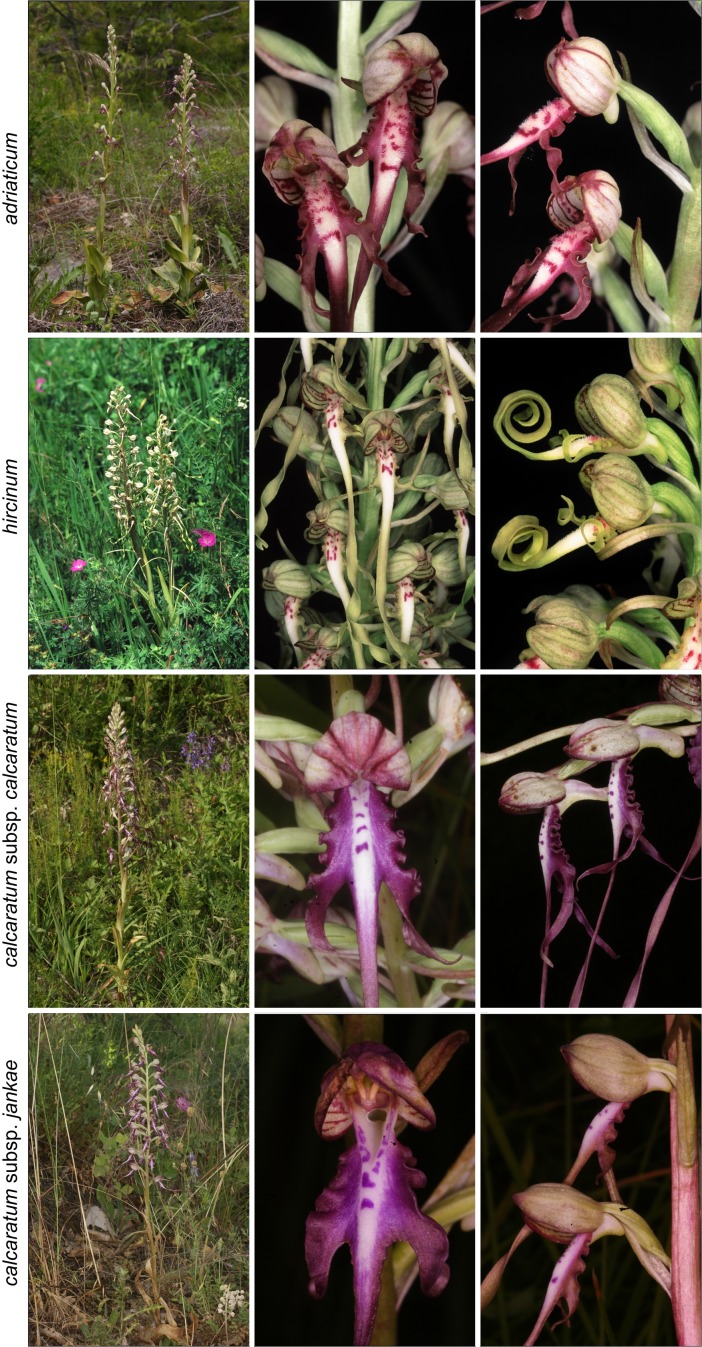
Typical flowers of taxa of *Himantoglossum* analysed in the present study, 2: *H. adriaticum*, *H. hircinum*, *H. calcaratum calcaratum* and *H. calcaratum jankae* (formerly *H. jankae* and *H. caprinum s.n.*). Images: Attila Molnár V.

**Figure 3:**
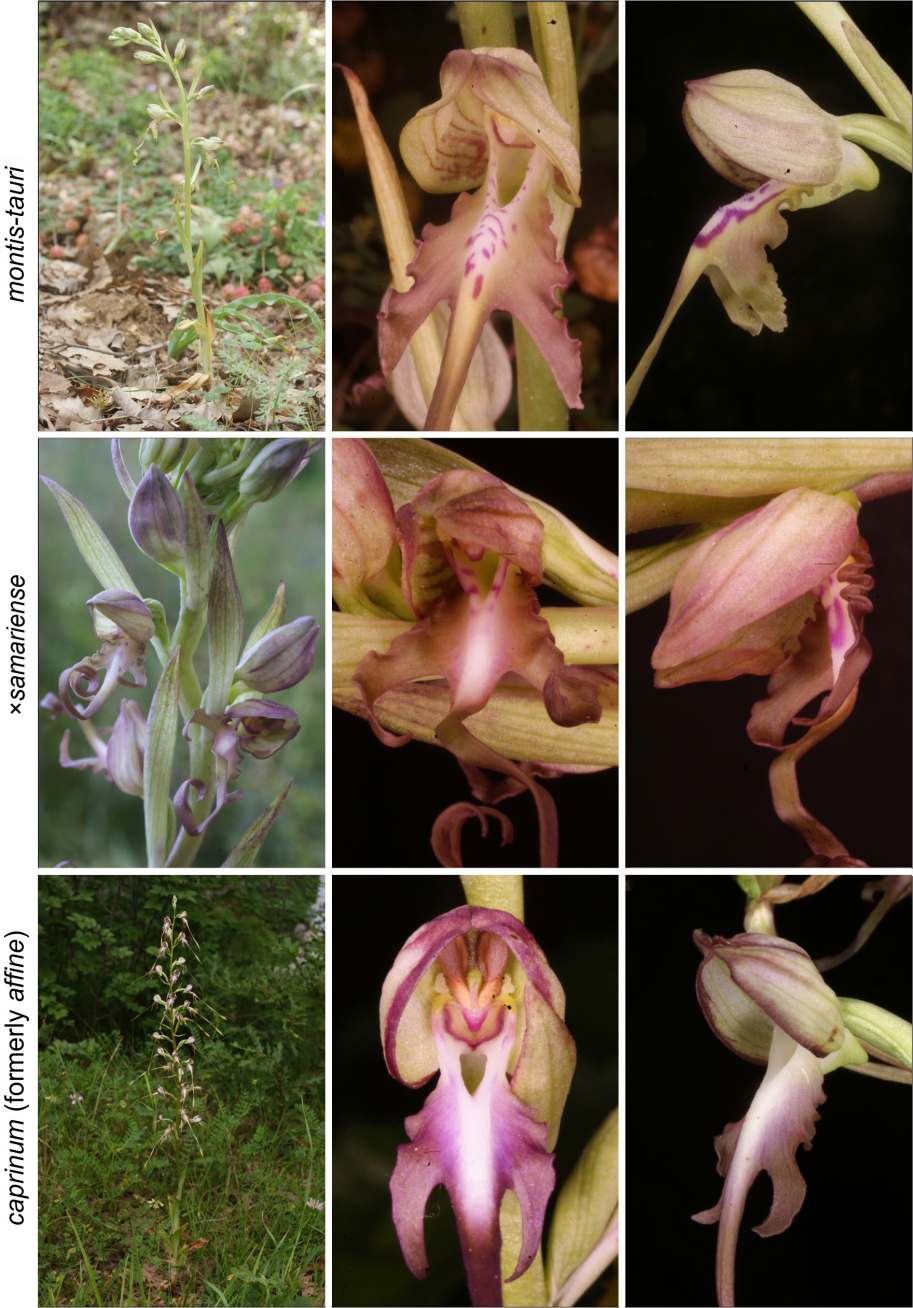
Typical flowers of taxa of *Himantoglossum* analysed in the present study, 3: *H. montis-tauri*, *H.* × *samariense* and *H. caprinum* (formerly *H. affine*). Images: Attila Molnár V.

The *Himantoglossum s.l.* clade (broadly termed the lizard orchids) is a particularly appealing group for detailed examination by evolutionary systematists. All members of *Himantoglossum* are large and charismatic plants, despite having a more diminutive putative sister-group in the form of *Steveniella satyrioides* (Delforge, 2000; Bateman et al., 2003). These terrestrial orchids (well-illustrated by Griebl, 2008) are vegetatively robust and produce long racemes of large flowers that are characterised by distinctive, unusually elaborate labella (Figs. 1–3).

Within the genus, two distinct levels of evolutionary divergence have become evident from phylogenetic studies. At the higher level, four groups—two of them previously viewed as arguably monotypic genera—are readily distinguishable using either morphological or molecular characters. Although the distinctiveness of these four groups is not in question, their evolutionary relationships have been much debated, detailed morphological accounts (e.g., Nelson, 1968; Teschner, 1980; Delforge, 1999) having graded into molecular phylogenetic studies toward the close of the 20th century (e.g., Pridgeon et al., 1997; Bateman et al., 2003; reviewed by Delforge, 1999; Bateman, 2012a). More recently, Sramkó et al. (2014) presented a multi-genome phylogenetic study of the group that was strongly supported statistically, and revealed substantial errors in each of the speculative classificatory systems and/or evolutionary scenarios devised by previous authors from morphological observations alone.

**Figure 4:**
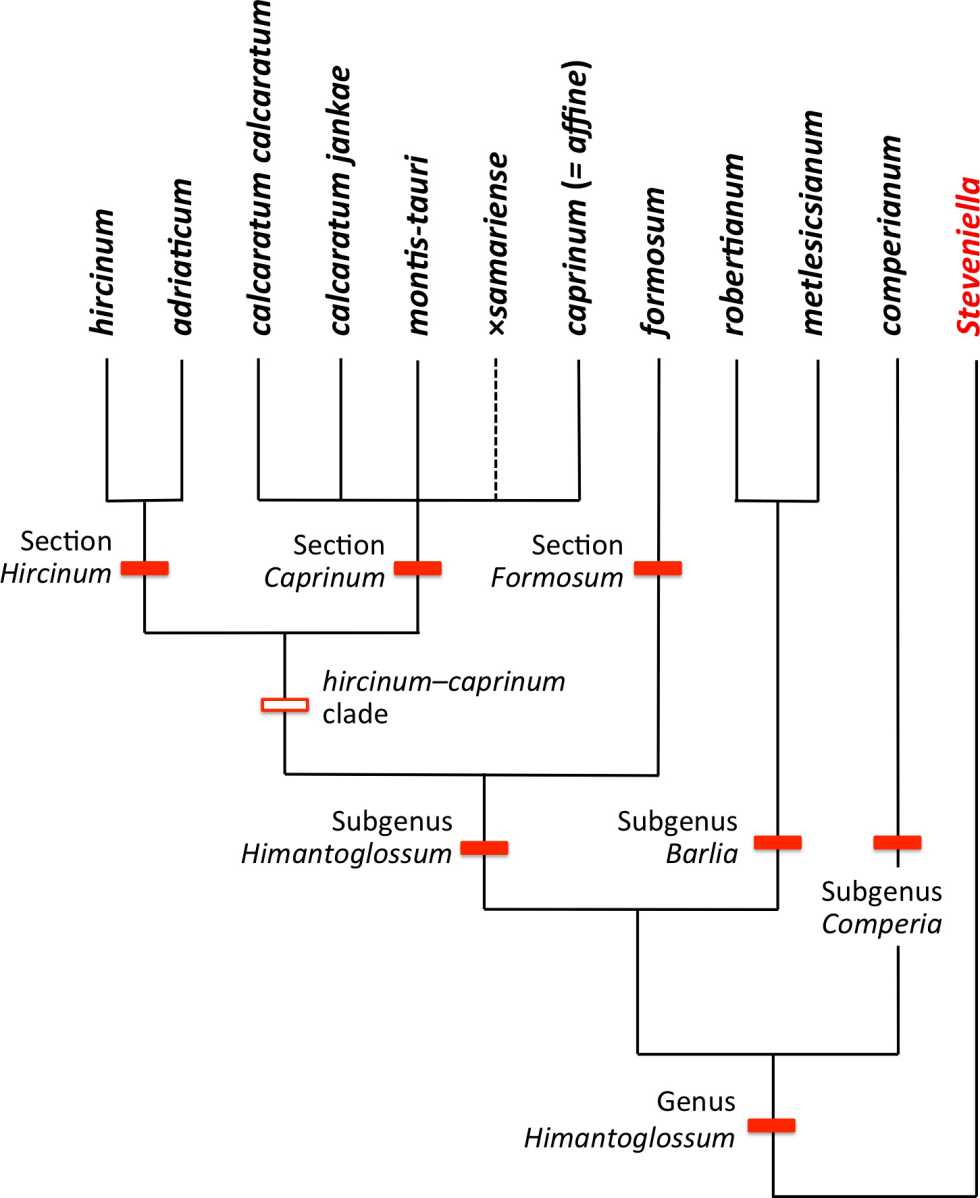
Taxonomy of the genus *Himantoglossum s.l.* generated by integrating the results of the present study with those of Sramkó et al. (2014).

At lower taxonomic levels, several taxa that are more subtly differentiated on either morphological or molecular grounds have at various times been recognised formally within the *H. robertianum* group (formerly the genus *Barlia*) and especially within the *H. hircinum–caprinum* group (Fig. 4). Such ambiguity inevitably leads to debates concerning the biological validity, optimal circumscription, and most appropriate taxonomic rank of each named taxon (cf. Nelson, 1968; Sundermann, 1973; Vermeulen, 1977; Moore, 1980; Sundermann, 1980; Teschner, 1980; Wood, 1983; Delforge, 1999; Bateman et al., 2003; Kreutz, 2004; Delforge, 2006; Kreutz, 2006; Vakhrameeva & Tatarenko, 2008; Bateman, 2012a; Sramkó & Molnár, 2012; Bateman et al., 2013; Sramkó et al., 2014; Tsiftsis, 2016). These ambiguities of taxonomic circumscription have contributed to, but have not been wholly responsible for, several past nomenclatural errors, including those that recently required the confusing transfer of the epithet *‘caprinum’* from one familiar taxon to another (Molnár et al., 2012; Sramkó et al., 2012).

These taxonomic and nomenclatural disputes inevitably have downstream consequences, not least because several of the more contentious taxa within *Himantoglossum* figure prominently in various international conservation initiatives (reviewed by Sramkó & Molnár, 2012; Sramkó et al., 2014). Indeed, we suspect that *all* named taxa in the genus feature in at least one conservation programme at the more localised scale of individual nations. Interest in the *Himantoglossum s.l.* clade is by no means confined to taxonomic issues. Along with many other European orchid species, *Himantoglossum* taxa have been studied at least superficially for their pollinator spectra (summarised by Claessens & Kleynen, 2011) and for their long-term, quantitative population demographics (e.g., Carey, 1999; Pfeifer, Heinrich & Jetschke, 2006) and phenology (e.g., Kreutz & Steinfeld, 2013; Biró & Bódis, 2015). A subset of these taxa have also featured in studies of climate change (Good, 1936; Carey, 1999; Pfeifer et al., 2009), the frequency of geitonogamy (Kropf & Renner, 2008), molecular evolution (Sramkó et al., 2014), or the ontogeny of unusually complex flowers (Fig. 11 of Bateman et al., 2013). Fortunately, recent studies (Molnár, 2011; Sramkó et al., 2014; Biró & Bódis, 2015; Tsiftsis, 2016) have brought knowledge of the systematics and biology of the eastern members of the clade significantly closer to levels previously achieved for the most westerly taxon, *H. hircinum* (reviewed by Carey & Farrell, 2002; Pfeifer et al., 2010; Bateman et al., 2013). When placed in a more explicit evolutionary context, members of the *Himantoglossum* clade have in addition contributed to discussions of founder effects on oceanic islands (Bateman, 2012b; Bateman et al., 2014) and of phenotypic convergence (Bateman et al., 2013).

### Project objectives and classificatory preamble

We here report a detailed *in situ* morphometric survey that, together with the molecular phylogenetic study of Sramkó et al. (2014), constitutes an integrated monograph of the expanded genus. The present morphometric survey arguably includes all of the named Eurasian taxa in the *Himantoglossum s.l.* clade other than the taxonomically controversial *H. galilaeum* (a putative endemic of the Levant). Our study was performed with the following objectives:

(1) Determining the optimal circumscriptions of, and most appropriate ranks for, taxa previously awarded formal names within the *Himantoglossum* clade.
(2) Identifying the most diagnostic characters that separate those re-circumscribed taxa, thereby facilitating their eventual re-description.
(3) Assessing the relative rates of morphological divergence (described here) versus molecular divergence (as documented by Sramkó et al., 2014) among the taxa.
(4) Summarising phenotypic character evolution within the clade, in search of patterns that could imply the intervention of particular underlying evolutionary processes.
(5) Speculating on the nature and relative significance of the inferred evolutionary processes.

Past taxonomic and nomenclatural treatments have together placed *Himantoglossum* in a frustratingly ambiguous quagmire of errors and uncertainties, where the most sensible (though impractical) solution would be to start afresh. In an attempt to avoid inducing yet more confusion, we have summarised as Fig. 4 our preferred (though still provisional) classification resulting from our studies (i.e., the present work, plus that of Sramkó et al., 2014). As far as possible, the names included in this Figure are used throughout the remainder of this text, though it is important to note that *“H. jankae”* is used throughout the text, Figures and Tables as an abbreviation of *“H. calcaratum jankae”*.

## Materials and Methods

### Fieldwork

**Figure 5:**
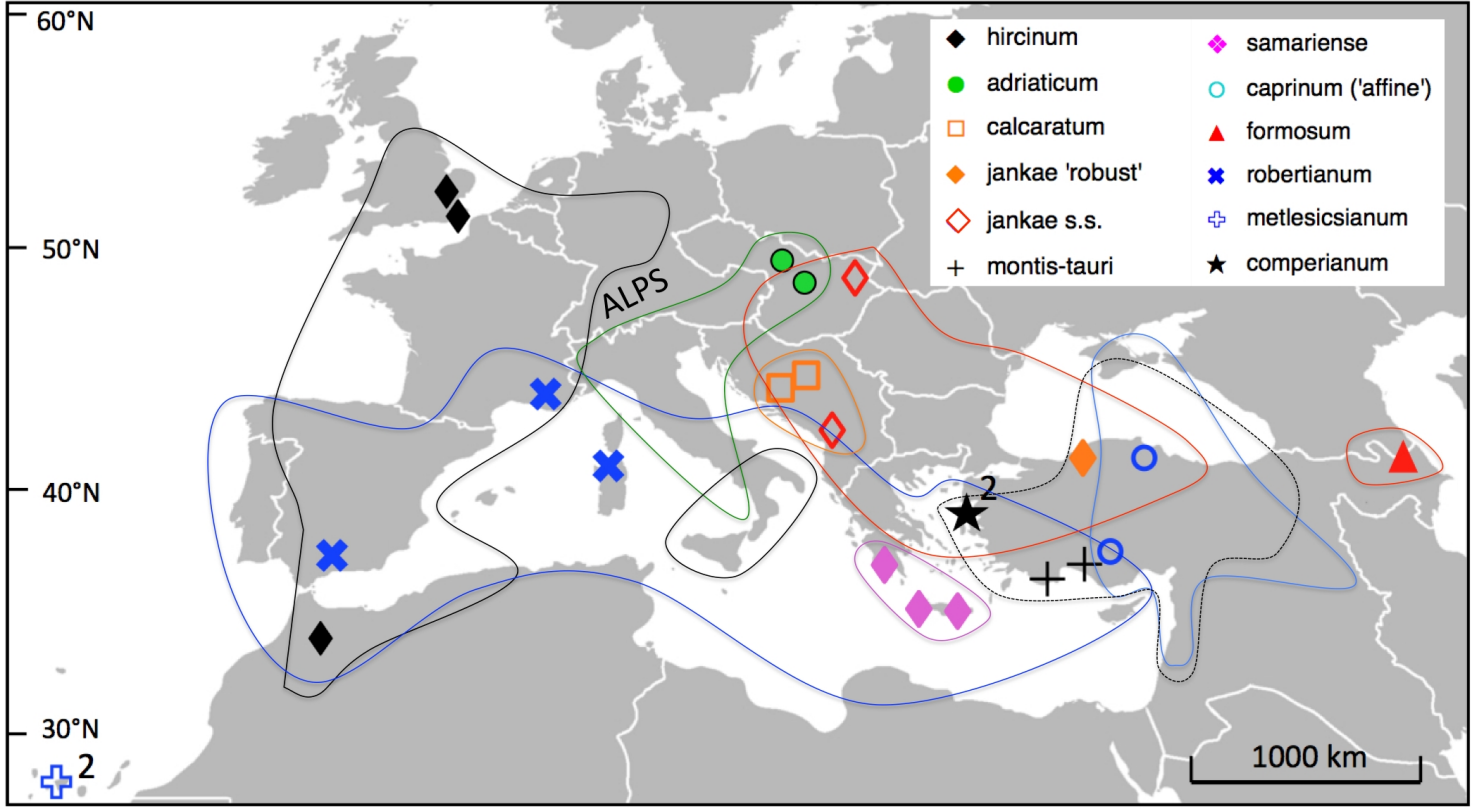
Distributions of the study taxa and locations of the populations measured.

Fieldwork for this study was conducted between spring 2010 and spring 2014, other than the measurements of *H. metlesicsianum* on Tenerife taken during 2001. We sampled across the entire Eurasiatic distribution of the genus *Himantoglossum s.l.* (including the former genera *Barlia* and *Comperia*), excepting only the Kurdish regions of Iran and Iraq (Fig. 5). Where necessary, collections were made under permits NE662, HNPD 45–2/2000, HNPD 250–2/2001, MDENCA 19642, and TTENCHA 60547. RMB (accompanied by PJ Rudall) focused on western European and North African populations plus *H. comperianum*, whereas GS and AMV toured central and eastern Europe. Silica-gel samples for potential DNA analysis were collected from a total of 131 populations (most of them listed in Table S1 of Sramkó et al., 2014), but only a carefully selected subset of 25 populations was subjected to detailed morphometric analysis for the present study. Two or three populations were studied of each named taxon other than the Caucasian endemic *H. formosum*, which was measured for only one population, and the Levant endemic *H. galilaeum*, which we were unable to pursue in the field.

We planned to study at least ten plants per population. However, as the majority of the populations of all species in the group are small, only 11 of the 25 study populations contained more than five measurable plants, and five populations yielded only a single measurable individual. In total, 152 plants were measured, the number of individuals scored per putative species ranging from three (*H.* ×*samariense*) to 30 (*H. jankae*, assuming that this species also includes *‘robustissimum’*); 115 of the measured plants belonged to the taxonomically problematic *hircinum–caprinum* clade.

### Morphometric characters measured

**Figure 6:**
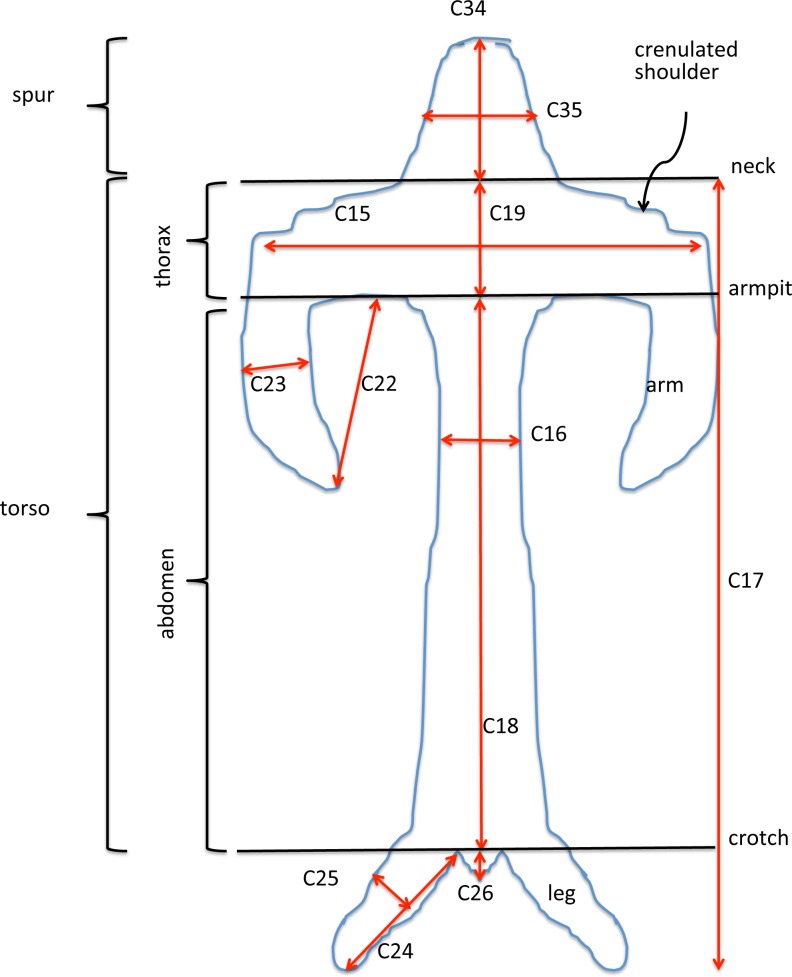
Explanation of labellum terminology and dimensions measured.

Our complete list of potentially scorable characters is presented as Appendix 3. While in the field we measured *in situ 12* vegetative characters and two floral characters (asterisked in Appendix 3); the remaining 37 characters were recorded on the same data sheet later in the same day. Field measurements were made using either a 15 cm steel rule bearing increments of 0.5 mm (RMB) or electronic calipers (GS + AMV). A flower–bract unit for subsequent measurement was, wherever possible, removed from a position one third to halfway from the base to the apex of the inflorescence, to minimise the effect of the flower-size decreases from the base to the apex of the inflorescence that are evident in most Eurasian orchid species (Bateman & Rudall, 2006). Each flower was initially placed in a numbered vial and later mounted onto double-sided adhesive tape attached to a filing card. Following measurement, these cards acted as herbarium vouchers (the permanent mounts are presently divided between **DE** and RMB’s private collection). Metric characters for most floral organs were measured at a resolution of 0.1 mm, using either a Leitz ×8 graduated ocular (RMB) or an electronic caliper (GS and AMV). Labellum dimensions, and our associated anthropomorphic terminology, are illustrated in Fig. 6.

The colours of the ‘limbs’ and the ‘torso’ margin of each labellum, and of the reverse (abaxial) surfaces of the outer perianth segments, were matched to the nearest one or two colour block(s) of the Royal Horticultural Society Colour Chart. They were later quantified through conversion to three CIE (Commission Internationale de I’Eclairage) coordinates. Two of these (‘x’ and ‘y’) define a position on a square grid superimposed onto a near-triangular array of colours that pale toward white at the centre of the triangle. The corners correspond with pure blue, pure green and pure red, respectively. Density of pigment was represented by a third coordinate (reflectivity, ‘Y’), which decreases in value outward from the centre of the triangle.

### Characters utilised

We rapidly compiled a preliminary list of 51 characters (Appendix 3). Beginning with a framework provided by RMB’s previous morphometric studies of European orchids (e.g., Bateman & Denholm, 1985; Bateman, Rudall & Moura, 2013), we then fine-tuned the initial character list to optimally fit the *Himantoglossum* clade.

Two characters included in this preliminary list were shown to be invariant. All study plants proved to have lanceolate rather than oblanceolate leaves (C14: Shape of longest leaf) and, much to our surprise, all had ‘abdomens’ that spiral sinistrally rather than dextrally (C20: Direction of spiralling of torso). The widest leaf was usually also the longest leaf, prompting omission from the analyses of the character representing width of widest leaf (C13) in favour of width of longest leaf (C12). Another character (C51: Distance separating viscidia) proved impractical to measure, as in all species other than *H. comperianum* the paired viscidia are laterally fused and consequently lack readily measurable separation. And the two field teams (RMB + PJ Rudall, GS + AMV) applied subtly different criteria to delineating the near-arbitrary distinction between basal leaves and cauline (bracteoidal) leaves, necessitating summation of values for the two original characters (C9 plus C10) into a single aggregate character (C9A). The above five characters were therefore omitted from all mathematical analyses. A further character (C39: Lateral teeth extending from lateral petals) was scored only after the mathematical analyses had been completed.

The surviving 45 characters described the stem and inflorescence and bracts (8), leaves (3), labellum (18), spur (3), lateral petals (2), lateral sepals (9) and gynostemium (2). They could alternatively be categorised as metric (35), meristic (4), multistate-scalar (5), and bistate (1). Subsets were also specified to represent vegetative characters (C1–C14: 11 of 14 characters usable) and anthocyanin-based pigmentation characters (C3, C27–C31, C41–C46: all 12 characters usable).

### Data analysis

Our chosen approach to data analysis and interpretation was both detailed and experimental. Morphometric data for individual plants were summarised on an Excel v14.3 spreadsheet. Mean values, plus sample standard deviations and coefficients of variation for all metric and meristic characters, were calculated for every character in each study population that yielded three or more measurable individuals. Univariate and bivariate analyses were summarised and presented using Deltagraph v5.6 (SPSS/Red Rock software, 2005).

The full morphometric matrix contained 152 individuals × 45 usable characters and contained only 1.1% missing values. Only two characters incurred more than 4% missing values. The first was basal bract length (C6: 24% missing), a character that was introduced only after data collection had begun. The second was width of longest leaf (C12: 8% missing), a character that was no longer measurable in some plants due to precocious, environmentally-induced desiccation. All calculated ratios were also omitted from the multivariate analyses as, by definition, they duplicated their constituent characters. The assembled data were analysed by multivariate methods using Genstat v14 (Payne et al., 2011).

The 45 surviving characters were used to compute a symmetrical matrix that quantified the similarities of pairs of data sets (i.e., plants) using the Gower Similarity Coefficient (Gower, 1971) on unweighted data sets scaled to unit variance. The resulting matrix was in turn used to construct a minimum spanning tree (Gower & Ross, 1969) and subsequently to calculate principal coordinates (Gower, 1966; Gower, 1985)—compound vectors that incorporate positively or negatively correlated characters that are most variable and therefore potentially diagnostic. Principal coordinates are especially effective for simultaneously analysing heterogeneous suites of morphological characters and have the additional advantage of comfortably accommodating missing values; ordinations have proven invaluable for assessing relationships among orchid species and populations throughout the last three decades (e.g., Bateman & Denholm, 1983; analytical approach reviewed in detail by Bateman, 2001).

Twelve separate multivariate analyses were conducted, differing in (a) whether the rows of data were individual scores or population means, (b) whether non-*hircinum–caprinum* group species were excluded, and (c) whether either the vegetative organ subset or pigmentation subset of characters was omitted from the analyses:

(A) All 152 individuals measured, all 45 characters included.
(B) All 152 individuals measured, all 11 vegetative characters excluded.
(C) All 152 individuals measured, all 12 pigmentation characters excluded.
(D) 115 individuals of the *hircinum–caprinum* clade only, all 44 characters included.
(E) 115 individuals of the *hircinum–caprinum* clade only, all 11 vegetative characters excluded.
(F) 115 individuals of the *hircinum–caprinum* clade only, all 12 pigmentation characters excluded.
(G) All 25 populations measured, all 46 characters included.
(H) All 25 populations measured, all 11 vegetative characters excluded.
(I) All 25 populations measured, all 13 pigmentation characters excluded.
(J) 17 populations of the *hircinum–caprinum* clade only, all 45 characters included.
(K) 17 populations of the *hircinum–caprinum* clade only, all 11 vegetative characters excluded.
(L) 17 populations of the *hircinum–caprinum* clade only, all 13 pigmentation characters excluded.

Compared with the above figures, the number of pigmentation characters increased by one in the population-level analyses because we judged it necessary to add a character indicating the proportion of plants within each population that bore any labellum markings (C30a). In addition, the character that in the matrix of individuals represented the length of ‘tail’ on the labellum (C26) was modified to simply represent the proportion of plants in each population that had developed ‘tails’, irrespective of tail length. A further character, position of lateral sepals (C48), became invariant (all plants scoring as state 1) in the six analyses that were restricted to the *hircinum–caprinum* clade.

For each multivariate analysis, the first four principal coordinates (PC1–PC4) were plotted together in pairwise combinations to assess the degree of morphological separation of individuals (and thereby of populations and taxa) in these dimensions, and pseudo-F statistics were obtained to indicate the relative contributions to each coordinate of the original variables.

### Journal nomenclatural statement

The electronic version of this article, produced in Portable Document Format (PDF), will represent a published work according to the International Code of Nomenclature for algae, fungi and plants (ICN). Hence, the new names and new combinations contained in the electronic version are effectively published under that Code from the electronic version alone. In addition, new nomenclatural combinations contained in this article that have been issued with LSID identifiers by International Plant Names Index (IPNI) will eventually be made available by the journal to the Global Names Index. The IPNI LSIDs can be resolved, and the associated information viewed, through any standard Web browser, by appending the LSID contained in this publication to the prefix “http://ipni.org/”. The online version of this work is archived and available from the following digital repositories: PeerJ, PubMed Central and CLOCKSS.

## Results

Table 1 gives population mean values for all 47 usable characters. These were subjected to a range of multivariate and univariate analyses, seeking to tease out biologically meanigful observations from an unusually complex dataset.

### Multivariate analyses

**Figure 7:**
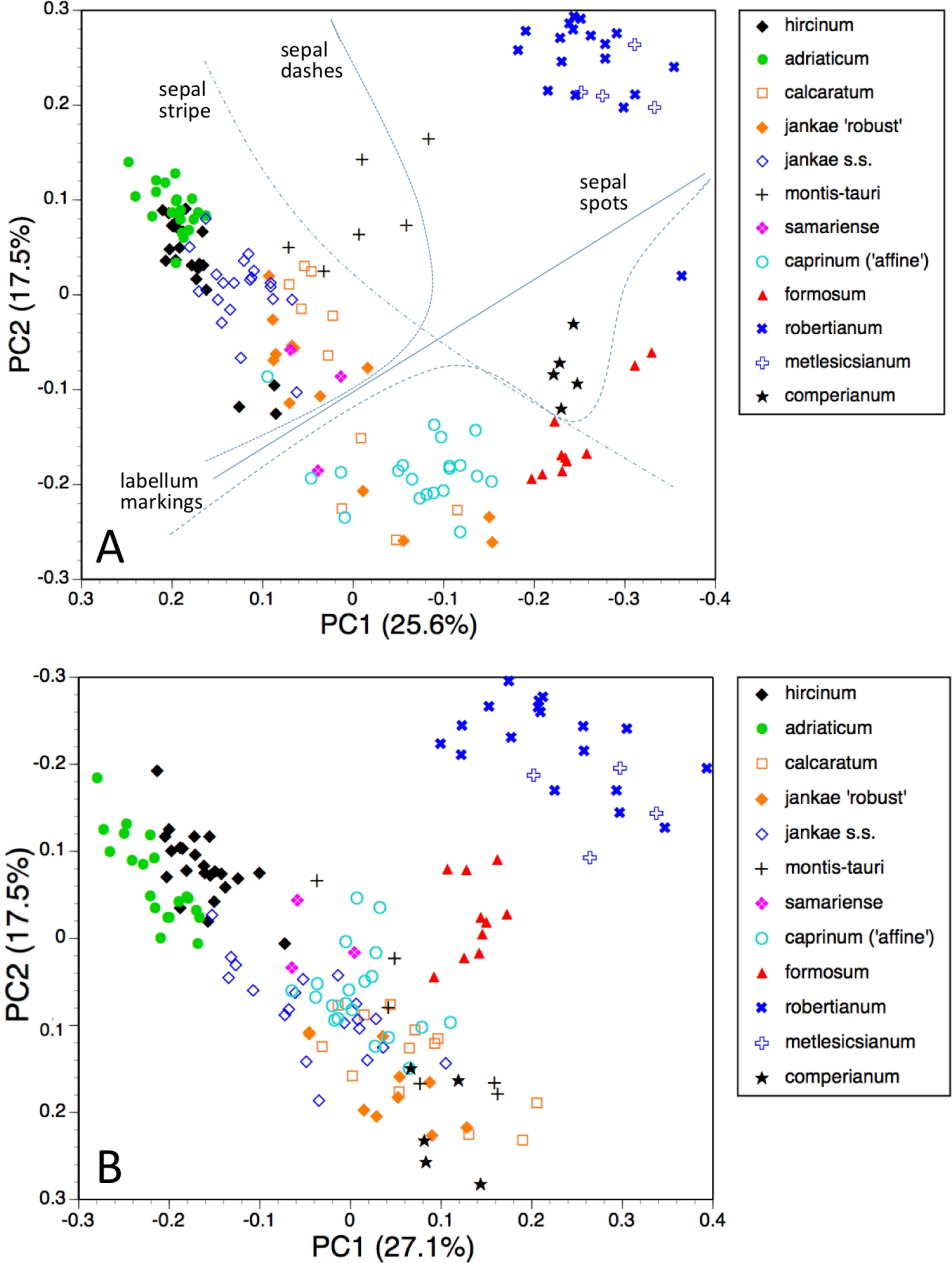
Principal coordinates plots for individual plants of all species. (A) All characters. (B) Pigmentation characters omitted. Characters contributing to the coordinates are given in Tables 2 and 3.

**Figure 8:**
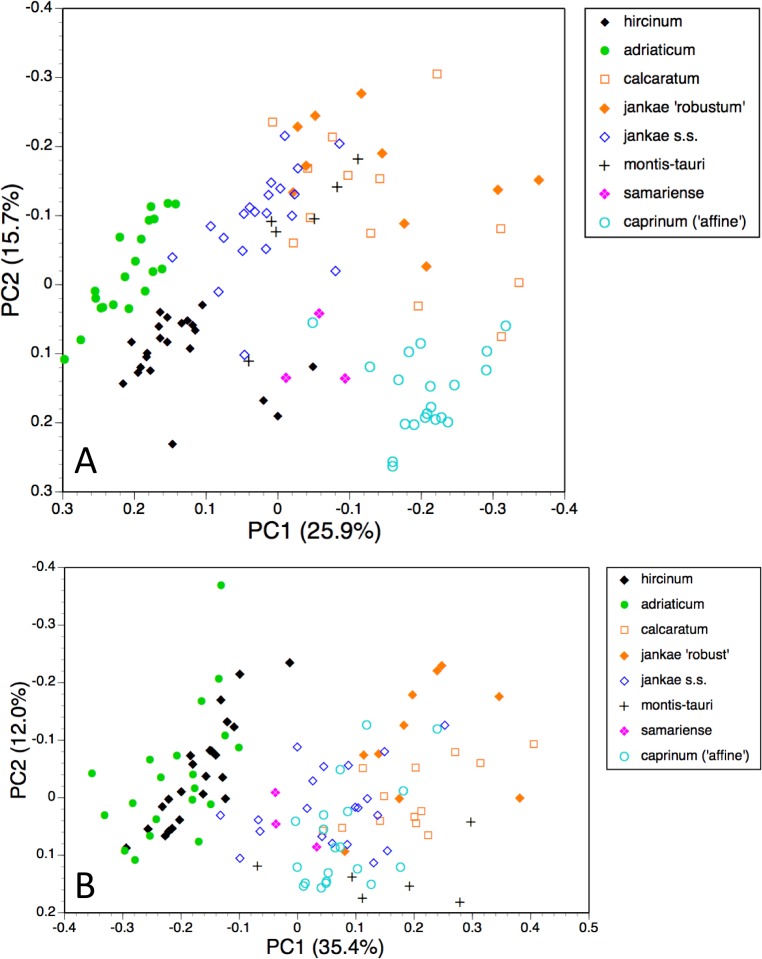
Principal coordinates plots for individual plants of the *hircinum*-*caprinum* clade only. (A) All characters. (B) Pigmentation characters omitted. Characters contributing to the coordinates are given in Tables 4 and 5.

**Figure 9:**
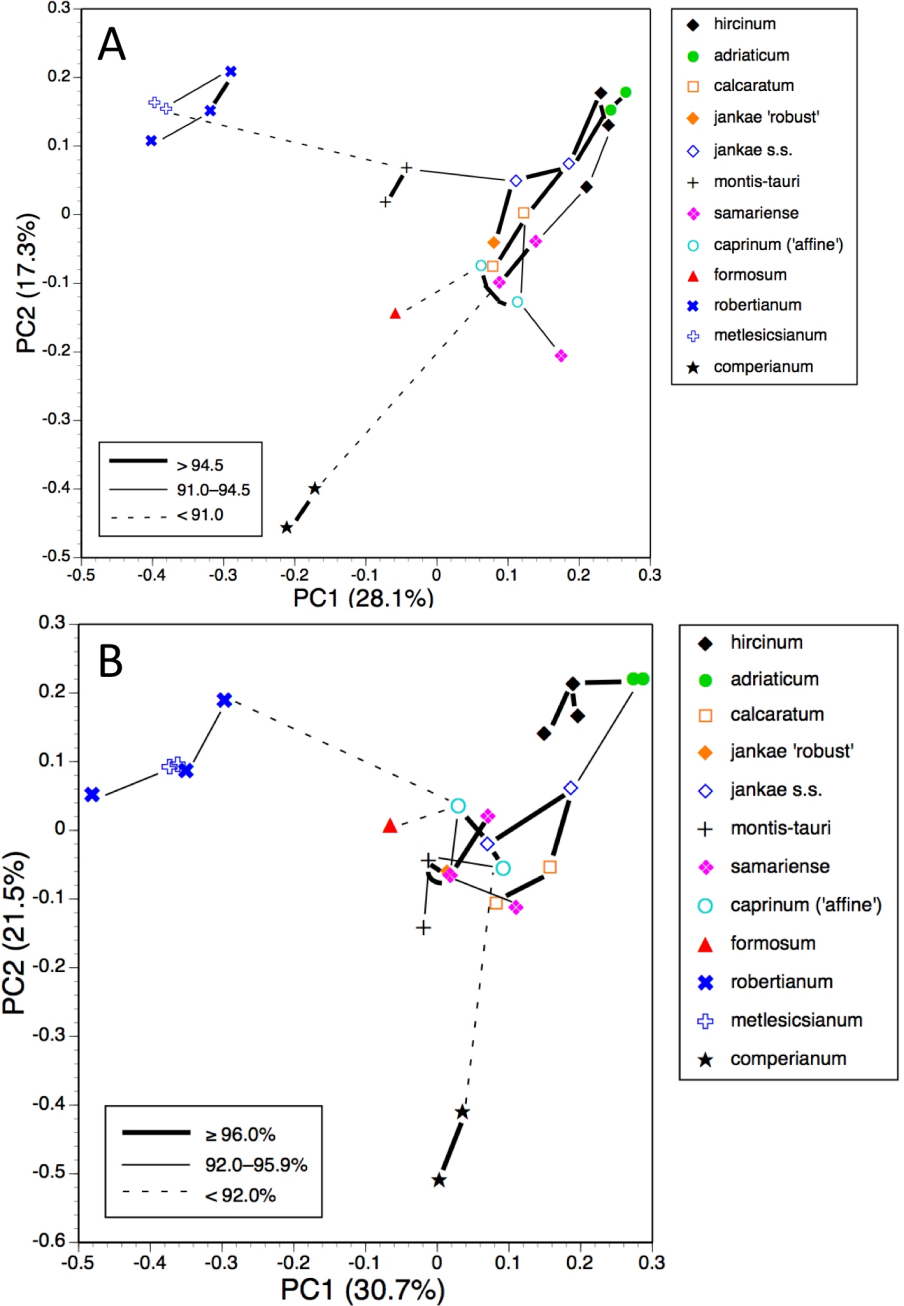
Principal coordinates plots and minimum spanning trees for populations of all species. (A) All characters. (B) Pigmentation characters omitted.

**Figure 10:**
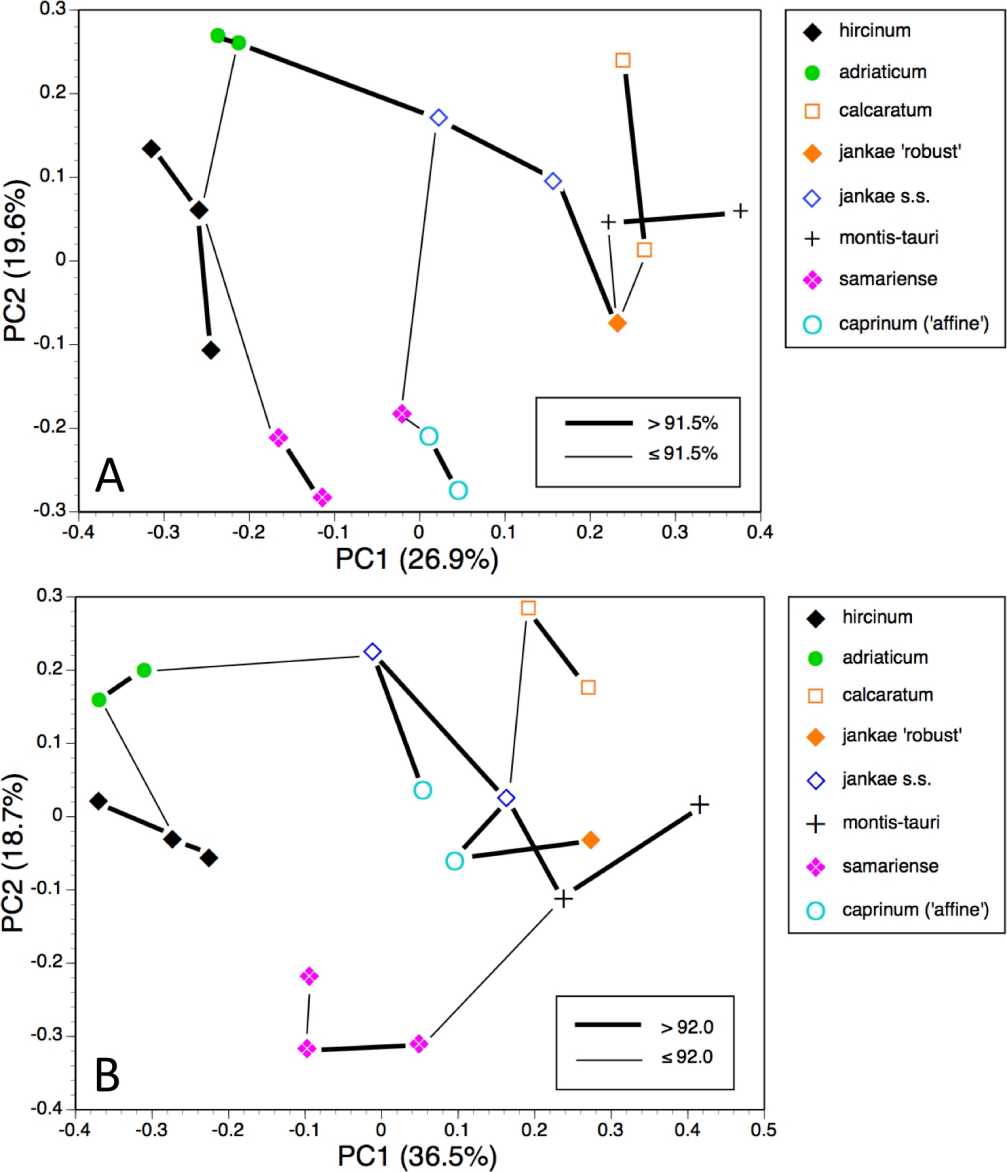
Principal coordinates plots and minimum spanning trees for populations of the *hircinum–caprinum* clade only. (A) All characters. (B) Pigmentation characters omitted.

Of the 12 principal coordinates analyses performed (listed as A–L in the ‘Materials and Methods’), eight proved to be more informative than the remaining four and hence form the core of this paper. The four analyses that were discarded were those based on reduced matrices lacking vegetative characters (analyses B, E, H, K above), which yielded results that were only marginally different from those obtained from the full matrices. Moreover, examination of the relative contributions of individual characters to each principal coordinate further emphasised that vegetative characters had proved to be relatively unimportant when analysing the *Himantoglossum s.l.* clade. The remaining eight plots of principal coordinates 1 versus 2 (lower-order coordinates are not depicted) are presented as Figs. 7–10, and the characters contributing most to the first four axes of each analysis are presented in Tables 2–5. Characters that are italicised in these Tables increase in value in parallel with increase in the value of the axis (i.e., from negative to positive scores); non-italicised characters increase in the converse direction.

**Table 1:** Character mean values for the 25 study populations. NM, not measured.

Population	Taxon	No. of plants measured	Stem height	Stem diameter	Stem pigment.	Inflor. length	Flower number	Basal bract length	Floral bract length	Ovary length
		*n*	1	2	3	4	5	6	7	8
UK N: Newmarket	*hircinum*	10	367	3.95	0.5	120	27.2	26.0	21.0	12.7
UK S: Sandwich	*hircinum*	10	317	5.18	1.0	150	50.9	NM	30.3	13.0
Ma: Ifrane	*hircinum*	3	380	7.97	0	143	68.5	45.0	27.3	12.7
Hu E: Nyirád	*adriaticum*	10	590	4.62	1.1	292	39.0	34.0	16.2	14.6
Hu W: Kőszeg	*adriaticum*	10	444	3.59	0.9	163	23.9	26.8	15.8	13.0
BiH: Sutjeska	*calcaratum*	12	622	4.11	0.1	217	25.3	36.7	25.8	16.8
Srb: Bačevci	*calcaratum*	1	610	5.22	1.0	243	31.0	29.0	16.3	17.3
Tr: Dereceören	*jankae* ‘robust’	10	543	6.15	1.4	275	51.4	53.1	39.8	15.5
MNe: Bukovik	*jankae s.s*.	10	436	4.60	1.4	214	31.3	31.2	20.6	13.6
Hu: Jósvafő	*jankae s.s*.	10	575	4.06	0.7	235	33.2	29.1	16.3	13.3
Tr S: Termessos	*montis-tauri*	2	550	4.93	0	259	24.0	NM	35.4	15.0
Tr N: Cevizli	*montis-tauri*	4	320	3.98	0	173	12.0	NM	34.1	12.8
Gr: Taigeti	*samariense*	1	225	6.00	0	NM	26.0	47.5	38.6	15.3
Cr E: Kato Simi	*samariense*	1	275	4.37	1.0	97	15.0	48.9	38.3	14.3
Cr W: Samaria	*samariense*	1	307	4.53	1.0	162	15.0	39.5	42.3	18.2
Tr S: Mehmetali	*caprinum*	10	530	5.30	0.6	267	35.2	NM	26.6	14.3
Tr N: Küçükçukur	*caprinum*	10	385	3.58	0.8	197	15.7	38.0	24.0	15.0
Az: Xuçbala	*formosum*	10	551	5.06	1.2	298	26.3	56.5	29.7	16.5
Sp: Torcal	*robertianum*	3	407	9.70	0.3	170	35.0	49.7	31.7	18.3
Fr: Var	*robertianum*	5	312	7.58	1.4	120	23.6	33.8	22.2	15.8
Sar: Pattamona	*robertianum*	10	448	7.37	0.9	198	41.9	NM	16.9	16.0
Ten N: Santiago	*metlesicsianum*	3	500	8.53	0	148	31.3	41.3	27.3	17.0
Ten S: Chirche	*metlesicsianum*	1	450	6.40	0	130	19.0	39.0	24.0	14.0
Les N: Sanctuario	*comperianum*	3	313	3.43	1.0	110	9.3	41.7	26.3	20.3
Les S: Olimbos	*comperianum*	2	385	4.40	0.5	105	13.5	54.0	23.0	16.5

Population	Taxon	Total leaf number	Length longest leaf	Width longest leaf	Lip width shoulders	Lip width abdomen	Lip max. length	Lip length torso	Lip length thorax	Lip crenulae no.
		9a	11	12	15	16	17	18	19	21
UK N: Newmarket	*hircinum*	8.6	105	NM	6.6	1.67	39.4	38.1	5.1	4.7
UK S: Sandwich	*hircinum*	10.6	96	31.3	7.3	1.71	44.6	43.7	4.8	4.9
Ma: Ifrane	*hircinum*	12.7	88	42.7	7.1	1.83	49.7	47.3	6.8	4.7
Hu E: Nyirád	*adriaticum*	9.5	122	27.7	5.1	1.29	49.5	41.5	6.0	6.8
Hu W: Kőszeg	*adriaticum*	9.7	108	20.7	4.6	1.09	51.0	49.3	6.8	6.8
BiH: Sutjeska	*calcaratum*	10.6	134	30.0	11.0	1.79	74.2	54.9	11.8	7.1
Srb: Bačevci	*calcaratum*	10.0	122	20.0	12.6	1.65	79.1	43.8	12.0	8.0
Tr: Dereceören	*jankae* ‘robust’	11.9	139	38.7	11.8	2.06	64.7	41.6	9.7	5.6
MNe: Bukovik	*jankae s.s*.	9.6	122	39.4	8.8	1.63	58.6	40.1	9.1	7.1
Hu: Jósvafő	*jankae s.s*.	10.6	103	26.0	8.7	1.53	65.9	45.2	9.9	5.2
Tr S: Termessos	*montis-tauri*	6.0	97	25.0	13.4	1.73	59.6	42.2	10.3	8.0
Tr N: Cevizli	*montis-tauri*	5.8	130	30.0	13.2	1.64	44.8	33.8	8.9	7.3
Gr: Taigeti	*samariense*	7.0	160	39.0	8.4	1.30	45.8	35.3	6.0	6.0
Cr E: Kato Simi	*samariense*	6.0	142	38.0	9.9	1.17	40.1	31.2	8.0	8.0
Cr W: Samaria	*samariense*	5.0	100	33.0	8.1	1.12	53.3	34.0	6.2	5.0
Tr S: Mehmetali	*caprinum*	8.9	125	38.7	8.2	1.74	48.5	39.8	8.7	4.8
Tr N: Küçükçukur	*caprinum*	8.4	75	20.8	8.8	1.54	58.2	42.6	8.8	4.4
Az: Xuçbala	*formosum*	9.1	130	27.8	8.1	2.58	25.5	23.6	11.4	9.8
Sp: Torcal	*robertianum*	6.3	259	76.0	11.0	6.23	19.3	15.2	8.9	2.0
Fr: Var	*robertianum*	6.2	117	58.0	10.2	5.64	16.7	13.6	7.6	2.6
Sar: Pattamona	*robertianum*	6.4	193	65.8	12.3	4.58	14.4	12.0	6.8	1.8
Ten N: Santiago	*metlesicsianum*	8.3	159	50.0	16.6	5.60	21.6	16.6	11.3	4.0
Ten S: Chirche	*metlesicsianum*	7.0	130	37.0	17.0	8.10	20.0	15.5	12.3	4.0
Les N: Sanctuario	*comperianum*	5.3	118	28.5	10.3	5.17	70.3	22.7	17.7	0
Les S: Olimbos	*comperianum*	5.5	75	24.0	8.5	3.75	63.0	21.0	17.5	0

Population	Taxon	Lip arm length	Lip arm width	Lip leg length	Lip leg width	Lip tail present	Lip limbs colour (x)	Lip limbs colour (y)	Lip limbs colour (Y)	Lip spots present
		22	23	24	25	26a	27	28	29	30a
UK N: Newmarket	*hircinum*	6.59	1.02	1.21	0.72	0.10	0.374	0.364	12.8	1.00
UK S: Sandwich	*hircinum*	12.88	1.04	1.47	0.76	0	0.400	0.364	9.2	1.00
Ma: Ifrane	*hircinum*	9.67	0.97	2.87	0.60	0	0.415	0.348	13.0	1.00
Hu E: Nyirád	*adriaticum*	7.90	0.98	7.46	0.80	0	0.371	0.347	11.6	1.00
Hu W: Kőszeg	*adriaticum*	6.41	0.86	1.82	0.60	0	0.346	0.298	13.1	1.00
BiH: Sutjeska	*calcaratum*	13.60	1.89	19.80	1.11	0	0.287	0.237	12.2	0.58
Srb: Bačevci	*calcaratum*	19.79	2.05	33.90	1.04	0	0.320	0.201	8.6	1.00
Tr: Dereceören	*jankae* ‘robust’	9.22	2.21	22.90	1.10	0	0.310	0.225	6.3	0.80
MNe: Bukovik	*jankae s.s*.	5.42	2.08	17.80	1.27	0	0.323	0.239	6.7	1.00
Hu: Jósvafő	*jankae s.s*.	4.05	1.78	20.40	0.89	0	0.336	0.286	19.2	1.00
Tr S: Termessos	*montis-tauri*	7.82	2.39	16.50	1.45	0	0.372	0.335	45.1	1.00
Tr N: Cevizli	*montis-tauri*	8.16	2.45	10.90	1.55	0	0.363	0.366	57.5	1.00
Gr: Taigeti	*samariense*	4.12	0.90	10.90	0.91	0	0.409	0.448	16.9	1.00
Cr E: Kato Simi	*samariense*	9.22	1.21	9.10	1.15	0	0.410	0.298	3.4	1.00
Cr W: Samaria	*samariense*	9.21	0.76	19.70	0.57	0	0.310	0.246	4.9	0
Tr S: Mehmetali	*caprinum*	3.78	1.59	8.78	1.28	0	0.373	0.266	11.7	0
Tr N: Küçükçukur	*caprinum*	5.04	1.56	14.60	1.14	0	0.354	0.314	12.8	0
Az: Xuçbala	*formosum*	1.56	1.43	1.85	1.34	0	0.284	0.189	8.7	0
Sp: Torcal	*robertianum*	6.10	2.97	5.10	3.77	0.67	0.404	0.331	21.3	0.67
Fr: Var	*robertianum*	5.02	2.66	4.46	3.72	0.20	0.388	0.325	14.4	1.00
Sar: Pattamona	*robertianum*	4.15	1.98	3.15	2.59	0.20	0.351	0.264	9.7	1.00
Ten N: Santiago	*metlesicsianum*	5.27	3.50	4.10	2.00	0.67	0.308	0.262	45.3	1.00
Ten S: Chirche	*metlesicsianum*	3.80	3.80	4.20	3.50	0	0.313	0.274	55.0	1.00
Les N: Sanctuario	*comperianum*	54.70	0.60	47.70	0.30	0	0.289	0.238	32.7	1.00
Les S: Olimbos	*comperianum*	34.00	0.50	42.00	0.40	0	0.298	0.265	46.0	1.00

Population	Taxon	Lip no. of spots	Lip distrib. spots	Pos. torso vs stem	Pos. arms vs torso	Spur length	Spur width	Spur curvature	Lateral petal length	Lateral petal width
		30	31	32	33	34	35	36	37	38
UK N: Newmarket	*hircinum*	14.7	1.9	2.0	4.0	2.20	1.95	4.4	6.66	1.39
UK S: Sandwich	*hircinum*	9.0	1.3	2.0	3.8	2.62	2.26	4.0	7.69	1.19
Ma: Ifrane	*hircinum*	12.3	1.0	2.0	3.0	3.07	2.37	5.0	8.87	1.63
Hu E: Nyirád	*adriaticum*	10.2	2.9	2.1	3.0	2.72	1.43	4.7	5.97	1.44
Hu W: Kőszeg	*adriaticum*	13.2	3.0	2.0	2.5	2.82	1.79	3.5	6.80	1.07
BiH: Sutjeska	*calcaratum*	6.9	1.9	1.0	3.3	11.15	3.48	4.3	9.06	2.09
Srb: Bačevci	*calcaratum*	17.0	3.0	2.0	4.0	7.49	2.24	4.0	8.33	1.70
Tr: Dereceören	*jankae* ‘robust’	4.3	2.0	1.0	3.2	5.25	2.73	3.9	10.86	2.76
MNe: Bukovik	*jankae s.s*.	14.5	3.0	1.5	3.7	5.76	3.20	4.2	10.60	2.51
Hu: Jósvafő	*jankae s.s*.	13.5	2.6	1.4	3.5	4.41	2.45	4.3	8.14	2.15
Tr S: Termessos	*montis-tauri*	22.0	3.0	1.0	5.0	6.54	4.06	5.0	10.37	2.94
Tr N: Cevizli	*montis-tauri*	20.3	3.0	1.0	5.0	5.60	3.31	4.0	9.48	2.25
Gr: Taigeti	*samariense*	11.0	2.0	3.0	3.0	6.04	2.87	3.0	9.07	1.66
Cr E: Kato Simi	*samariense*	12.0	2.0	2.0	3.0	5.12	2.67	4.0	11.16	2.10
Cr W: Samaria	*samariense*	0	0	3.0	3.0	7.19	2.48	5.0	9.66	1.97
Tr S: Mehmetali	*caprinum*	0	0	1.5	3.4	5.88	3.41	3.3	9.66	2.28
Tr N: Küçükçukur	*caprinum*	0	0	1.1	2.9	6.45	2.80	4.4	9.90	2.24
Az: Xuçbala	*formosum*	0	0	1.0	1.6	11.80	2.08	5.0	9.43	2.58
Sp: Torcal	*robertianum*	25.0	2.0	1.7	3.3	5.43	3.33	4.0	9.93	3.33
Fr: Var	*robertianum*	12.8	2.0	1.4	2.8	5.34	3.74	4.0	9.18	3.32
Sar: Pattamona	*robertianum*	17.0	3.0	1.6	3.1	4.83	3.28	3.8	6.88	1.94
Ten N: Santiago	*metlesicsianum*	15.3	2.3	1.0	2.0	5.30	3.73	4.0	8.47	2.30
Ten S: Chirche	*metlesicsianum*	20.0	3.0	1.0	2.0	5.60	3.40	4.0	7.80	2.30
Les N: Sanctuario	*comperianum*	42.0	3.0	2.0	3.0	15.70	2.53	5.0	13.30	1.53
Les S: Olimbos	*comperianum*	17.0	2.0	2.0	3.0	14.35	2.75	5.0	13.05	1.35

Population	Taxon	Lateral sepal length	Lateral sepal width	Sepal colour (x)	Sepal colour (y)	Sepal colour (Y)	Sepal marginal stripe	Sepal internal lines	Sepal internal dots	Position lateral sepals
		40	41	42	43	44	45	46	47	48
UK N: Newmarket	*hircinum*	8.91	4.50	0.377	0.438	83.0	1.0	1.00	0.90	1.0
UK S: Sandwich	*hircinum*	10.27	5.24	0.373	0.454	33.0	1.0	1.00	1.00	1.0
Ma: Ifrane	*hircinum*	11.93	4.87	0.357	0.421	58.0	1.0	1.00	0	1.0
Hu E: Nyirád	*adriaticum*	8.29	4.60	0.319	0.297	53.8	1.0	1.00	1.00	1.0
Hu W: Kőszeg	*adriaticum*	8.88	4.28	0.336	0.219	11.3	1.0	1.00	1.00	1.0
BiH: Sutjeska	*calcaratum*	13.78	6.68	0.322	0.302	27.1	1.0	0.83	0.67	1.0
Srb: Bačevci	*calcaratum*	12.54	6.01	0.320	0.201	8.6	1.0	1.00	1.00	1.0
Tr: Dereceören	*jankae* ‘robust’	15.22	7.08	0.301	0.240	20.7	1.0	1.00	0.60	1.0
MNe: Bukovik	*jankae s.s*.	14.15	7.35	0.343	0.294	59.0	1.0	1.00	1.00	1.0
Hu: Jósvafő	*jankae s.s*.	12.16	6.19	0.349	0.358	59.6	1.0	1.00	0.80	1.0
Tr S: Termessos	*montis-tauri*	15.98	6.85	0.348	0.350	57.6	0	1.00	1.00	1.0
Tr N: Cevizli	*montis-tauri*	13.39	6.57	0.341	0.356	53.0	0.5	0.75	1.00	1.0
Gr: Taigeti	*samariense*	13.88	5.39	0.416	0.374	23.3	1.0	1.00	0	1.0
Cr E: Kato Simi	*samariense*	14.78	6.06	0.408	0.236	7.6	1.0	1.00	0	1.0
Cr W: Samaria	*samariense*	13.14	5.45	0.324	0.356	58.7	1.0	1.00	0	1.0
Tr S: Mehmetali	*caprinum*	13.15	6.29	0.332	0.322	49.2	1.0	0.10	0	1.0
Tr N: Küçükçukur	*caprinum*	13.16	6.25	0.345	0.391	75.7	1.0	0.30	0.10	1.0
Az: Xuçbala	*formosum*	12.28	5.28	0.274	0.197	9.6	0.8	0	0	2.3
Sp: Torcal	*robertianum*	12.40	7.47	0.385	0.326	32.0	0	0	0.67	2.3
Fr: Var	*robertianum*	11.34	6.06	0.314	0.248	20.0	0	0	1.00	2.2
Sar: Pattamona	*robertianum*	9.63	5.45	0.306	0.248	35.6	0	0	1.00	2.7
Ten N: Santiago	*metlesicsianum*	12.47	6.10	0.309	0.250	35.7	0	0	1.00	2.3
Ten S: Chirche	*metlesicsianum*	11.50	6.00	0.308	0.233	26.0	0	0	1.00	3.0
Les N: Sanctuario	*comperianum*	19.20	7.47	0.459	0.317	8.0	0	0	0	1.0
Les S: Olimbos	*comperianum*	19.00	6.90	0.457	0.317	8.0	0	0	0	1.0

Population	Taxon	Column length	Column width	Lateral petal teeth pres.	Lip length abdomen
		49	50	39	18a
UK N: Newmarket	*hircinum*	4.02	2.42	0.20	33.0
UK S: Sandwich	*hircinum*	3.80	2.62	<1	38.9
Ma: Ifrane	*hircinum*	4.23	3.33	<1	40.5
Hu E: Nyirád	*adriaticum*	3.12	1.50	0	35.5
Hu W: Kőszeg	*adriaticum*	3.85	2.16	?	42.5
BiH: Sutjeska	*calcaratum*	6.26	3.44	0.08	43.1
Srb: Bačevci	*calcaratum*	5.99	2.96	0.10	31.8
Tr: Dereceören	*jankae* ‘robust’	5.82	2.88	0	31.9
MNe: Bukovik	*jankae s.s*.	6.12	2.41	0.10	31.0
Hu: Jósvafő	*jankae s.s*.	4.60	2.02	?	35.3
Tr S: Termessos	*montis-tauri*	8.45	3.12	0	31.9
Tr N: Cevizli	*montis-tauri*	7.09	2.92	0	34.9
Gr: Taigeti	*samariense*	5.67	3.18	0	29.3
Cr E: Kato Simi	*samariense*	6.23	3.18	0	23.2
Cr W: Samaria	*samariense*	6.16	2.89	0	27.8
Tr S: Mehmetali	*caprinum*	6.97	3.46	0	31.1
Tr N: Küçükçukur	*caprinum*	6.80	2.95	0	33.8
Az: Xuçbala	*formosum*	7.08	3.33	0.73	12.2
Sp: Torcal	*robertianum*	7.05	3.40	?	6.3
Fr: Var	*robertianum*	6.80	4.20	?	6.0
Sar: Pattamona	*robertianum*	6.60	4.19	?	5.2
Ten N: Santiago	*metlesicsianum*	NM	3.67	?	5.3
Ten S: Chirche	*metlesicsianum*	NM	3.80	?	3.2
Les N: Sanctuario	*comperianum*	6.15	3.55	1.00	5.0
Les S: Olimbos	*comperianum*	4.95	3.60	1.00	3.5

#### Individual plants, all taxa present

The first two coordinates based on analysis of individuals of all taxa for all usable characters (Fig. 7A) together account for 43% of the total variance, and work together to organise the plants in a diagonal array. All members of the *hircinum–caprinum* clade other than *H. montis-tauri* form a near-linear arrangement from the top-left to the mid-bottom of the plot, whereas the morphologically distinctive subgenus *Barlia* is isolated in the top-right quadrant. Placed between these two groups as separate clusters are *H. montis-tauri*, *H. formosum* and *H. comperianum*. Both coordinates are dominated by markings found on the sepals and/or perianths (Table 2). All individuals located below the solid line superimposed onto Fig. 7A lack any internal markings on the sepals and all but*H. comperianum* lack discrete labellum markings. Larger gynostemia and broader ‘abdomens’ also help to separate the *hircinum–caprinum* clade (left) from the remainder on the first coordinate. The considerably less informative third and fourth coordinates are not depicted here. The third coordinate partially separates *H. hircinum*, *H. adriaticum* and *H. caprinum* from the remaining taxa on the basis of the larger sepals and labella of the latter, whereas the fourth coordinate uses primarily the diffuse background colours of the sepals and labella to wholly separate the purple-flowered *H. formosum* from the paler, greenish-flowered *H. caprinum* and *H. montis-tauri*.

The main consequence of omitting the 12 pigmentation characters from the full matrix was to collapse *H. montis-tauri* and *H. comperianum* into the main group of plants (Fig. 7B), demonstrating that their apparent morphological distinctiveness relies heavily on anthocyanin-based characters. Their downward displacement on the second coordinate leaves only *H. formosum* as morphologically intermediate between the main group and subgenus *Barlia*. Predictably, the dimensionality of the variation is reduced, such that the first two coordinates account for an increased 47% of the total variance. In compensation, *H. adriaticum* becomes separated (just) from *H. hircinum* on the first coordinate, and a narrow discontinuity opens between them and *H. jankae*—morphologically the closest member to section *hircinum* of the remainder of the *hircinum–caprinum* group. This separation of *H. hircinum* and especially *H. adriaticum* from the remaining taxa reflects several characters, including their comparatively small columns, narrow shoulders and torsos, and narrow limbs (Table 3). The much weaker third coordinate (not shown) widely separates *H. comperianum* and, to a lesser degree, *H. formosum* from the remainder on the basis of their long, curved spurs and, in the case of *H. comperianum*, their few-flowered inflorescences. The fourth coordinate separates *H. formosum* from *H. comperianum*, due primarily to its longer stem and correspondingly longer inflorescence (Table 3).

#### Individual plants, taxa restricted to *hircinum–caprinum* clade

*Himantoglossum comperianum*, *H. robertianum*, *H. metlesicsianum* and *H. formosum* were then removed from the analysis in order to better explore the more subtle variation evident within the *hircinum–caprinum* clade (Fig. 8).

Despite these additional constraints, the first two coordinates utilising all characters account for a similar proportion (42%) of the total variance (Fig. 8A). Although a wider range of dimensions of floral organs now dominates the first coordinate (Table 4), the relative positions of the taxa on the first coordinate resemble those evident in the all-taxon analysis (Fig. 7A): *hircinum* plus *adriaticum* occupy one end of the coordinate and *caprinum* plus *jankae* ‘robust’ occupy the other. The second coordinate summarises a wide, heterogeneous range of characters, including several that represent anthocyanin markings. It largely separates *hircinum*, *caprinum* and ×*samariense* from the remainder, including narrowly distinguishing *H. adriaticum* from *H. hircinum*. The much weaker third coordinate separates the anthocyanin-deficient, vegetatively comparatively weak *H. montis-tauri* from the remainder.

Removing pigmentation characters (Fig. 8B) increases the amount of variance accommodated by the first coordinate, which now dictates a narrow discontinuity that separates *hircinum* plus *adriaticum* from the remaining taxa, primarily on the basis of their small sepals, though many other characters also contribute to the coordinate (Table 5). The second coordinate is almost entirely determined by characters that represent vegetative vigour and consequently has limited taxonomic relevance, serving primarily to distinguish the comparatively small-bodied *H. montis-tauri*. The third coordinate (not shown) succeeds only in partially separating *adriaticum*, *calcaratum* and *jankae s.s.* from the remainder.

**Table 2:** Characters contributing to the first four principal coordinates for individual plants, all taxa and all characters included (see Fig. 7A).

Coordinate	PC1	PC2	PC3	PC4
Percentage of variance accounted for	25.6	17.5	9.6	8.2
Taxonomic significance	‘*Barlia*’ + ‘*Comperia*’ +*formosum* : REST	‘*Barlia*’ : REST	*hircinum*+*adriaticum*+*caprinum* : *jankae*+*calcaratum*	*formosum* : *caprinum*+*montis-tauri*
Contributory characters, listed in order of decreasing contribution	Sepal interior dashes*	Sepal interior spots*	Sepal width*	Sepal colour x*
	Lip torso width	Lip markings distribution*	Sepal length	Lip colour Y*
	Column width	Sepal peripheral stripe	Lip leg length	*Sepal colour y*
	Column length	*Lip markings number*	Lip shoulder width	Stem pigmentation
	Sepal position	Lip overall length	Lip overall length	Inflorescence length
	*Sepal peripheral stripe*	Petal length	Lip length to armpit	Lip colour y
	*Lip torso length*	Sepal length	Lip arm width	*Lip arm length*

**Table 3:** Characters contributing to the first four principal coordinates for individual plants, all taxa included but pigmentation characters omitted (see Fig. 7B).

Coordinate	PC1	PC2	PC3	PC4
Percentage of variance accounted for	27.1	20.0	9.5	8.4
Taxonomic significance	‘*Barlia*’ + ‘*Comperia*’ +*formosum* : REST : *hircinum*+*adriaticum*	‘*Barlia*’ : REST : ‘*Comperia*’	‘*Comperia*’ : REST	*formosum* : REST
Contributory characters, listed in order of decreasing contribution	*Column length*	Lip overall length*	Spur curvature*	Inflorescence length*
	*Lip ‘shoulder’ width*	*Sepal length*	Flower number*	Stem height*
	*Lip torso width*	*Lip leg length*	*Leaf number*	Spur curvature
	*Column width*	*Petal length*	Spur length	Lip crenulae number
	*Lip leg width*	*Lip length to ‘armpit’*		Leaf number
	*Petal width*	Sepal position		Flower number
	*Lip arm width*	*Sepal width*		Bract, length basal
	*Spur median width*	*Lip torso length*		
	*Sepal position*	*Spur length*		

**Table 4:** Characters contributing to the first four principal coordinates for individual plants of the *hircinum–caprinum* clade only, all characters included (see Fig. 8A).

Coordinate	PC1	PC2	PC3	PC4
Percentage of variance accounted for	25.9	15.7	8.7	6.8
Taxonomic significance	*hircinum*+*adriaticum* : REST : *caprinum*+*jankae* ‘rob.’	*hircinum*+*caprinum*+×*samariense* : REST	*montis-tauri* : REST	*hircinum* (pp) : REST
Contributory characters, listed in order of decreasing contribution	Column length*	Lip overall length	Lip colour Y*	Flower number
	Sepal length*	Crenulae number	Flower number	Stem diameter
	Petal length	Lip markings distribution	*Sepal colour Y*	Leaf width
	Spur width	Sepal interior spots	Sepal peripheral stripe	Sepal colour y
	Spur length	Sepal interior dashes	Stem pigmentation	
	Sepal width	Arm width	Leaf number	
	*Sepal interior spots*	Lip length to armpit	*Sepal colour y*	
	Column width	Stem height	*Lip markings number*	
	Petal width	*Lip colour x*		
	*Lip markings distribution*	Sepal width		
	Lip shoulder width	*Sepal colour x*		
	Lip length to armpit	Lip shoulder width		
		*Sepal colour y*		
		*Lip colour y*		

**Table 5:** Characters contributing to the first four principal coordinates for individual plants of the *hircinum–caprinum* clade only, pigmentation characters omitted (see Fig. 8B).

Coordinate	PC1	PC2	PC3	PC4
Percentage of variance accounted for	35.4	12.0	9.2	7.7
Taxonomic significance	*hircinum*+*adriaticum* : REST	*montis-tauri* : REST	*adriaticum*+*calcaratum*+*jankae* s.s. : REST	NONE
Contributory characters, listed in order of decreasing contribution	Sepal length*	Number of flowers*	Lip overall length	Spur curvature
	Sepal width*	Stem diameter*	*Leaf width*	
	*Lip shoulder width*	Number of leaves	Stem height	
	*Petal length*	Inflorescence length	*Floral bract length*	
	*Column length*	Stem height	Spur curvature	
	*Lip length to armpit*	Leaf width		
	*Lip arm width*	Lip torso length		
	*Petal width*	Leaf length		
	*Lip torso width*	Basal bract length		
	*Spur length*			
	*Lip overall length*			
	Lip angle torso vs stem			
	*Lip leg length*			
	*Column width*			
	*Lip leg width*			
	*Ovary length*			

The most striking feature of both ordinations is that the positions of plants across the plot broadly reflect their relative longitudes, western European plants being confined to the left-hand region of the plot and plants of Asia Minor being confined to the right (Figs. 8A and 8B).

#### Population means, all taxa present

Ordinations of population means also have superimposed upon them the corresponding minimum spanning trees, which are useful for indicating the relative strengths of the links connecting populations. Theory predicts that populations of the same species should most closely resemble each other rather than populations of other species. Ideally, to optimise this similarity test, more populations of each species would have been measured by us (indeed, *H. formosum* is represented in our matrix by only one population and so is effectively untestable in this way). Also, within several populations, sample sizes are undesirably small, epitomised by the three populations of *H. ×samariense*—the single measurable plant found in each population risks incurring serious sampling error when we are obliged to view that plant as representing the entire source population.

The plot using all characters for all populations (Fig. 9A) encompasses a similar amount of variation as the plots for individual plants (45%). It greatly separates from the main cluster both *H. comperianum* (on both coordinates) and subgenus *Barlia* (on the first coordinate only); they are linked to the main cluster of populations only weakly, as is *H. formosum*, which is distanced from all other populations on the plot of the third and fourth coordinates (not shown). *Himantoglossum montis-tauri* is also somewhat distanced from the main cluster. Most conspecific populations link to each other strongly, the exception being the single plant representing the population of *H. ×samariense* from its type locality in the Samaria Gorge of western Crete; this instead links weakly to *H. caprinum*.

Omitting pigmentation characters from the analysis (Fig. 9B) increased the variance accounted for to 52% but yielded broadly similar positioning of most populations. The most sigificant changes were that *H. montis-tauri* was pulled deeper into the main cluster of populations, whereas *H. hircinum* and *H. adriaticum* became attached to each other rather than to *H. jankae*, and were further distanced from section *caprinum*. In addition, the Bukovki population of *H. jankae* became interpolated between the two populations of *H. caprinum*.

#### Population means, taxa restricted to *hircinum–caprinum* clade

Restricting the population-level analysis to the *H. hircinum–caprinum* clade (Fig. 10A) considerably reduced the degree of disparity among maximum similarities—in other words, the taxonomic relationships appear more egalitarian. Conspecific populations are reliably connected with strong links, *H. jankae* seemingly occupying a central position within the clade. But as in the all-taxon analysis, the Samaria population of *H. ×samariense* is linked to *H. caprinum*. And in this case, the two remaining *H. ×samariense* ‘populations’ (strictly, plants) are linked, albeit weakly, to the Sandwich population of *H. hircinum*. The third coordinate (not shown) primarily separated *H. montis-tauri* from the remainder.

Omitting pigmentation characters from the analysis (Fig. 10B) once again unified the three populations of *H. ×samariense* (this time weakly attached to *H. montis-tauri*), and as in the all-taxa analysis, the two populations of *H. caprinum* became separated. More surprisingly, the single population of *H. jankae* ‘robust’ became strongly attached to the Mehmetali population of *H. caprinum*. The third and fourth coordinates offered no taxonomic separation.

### Univariate analyses

**Figure 11:**
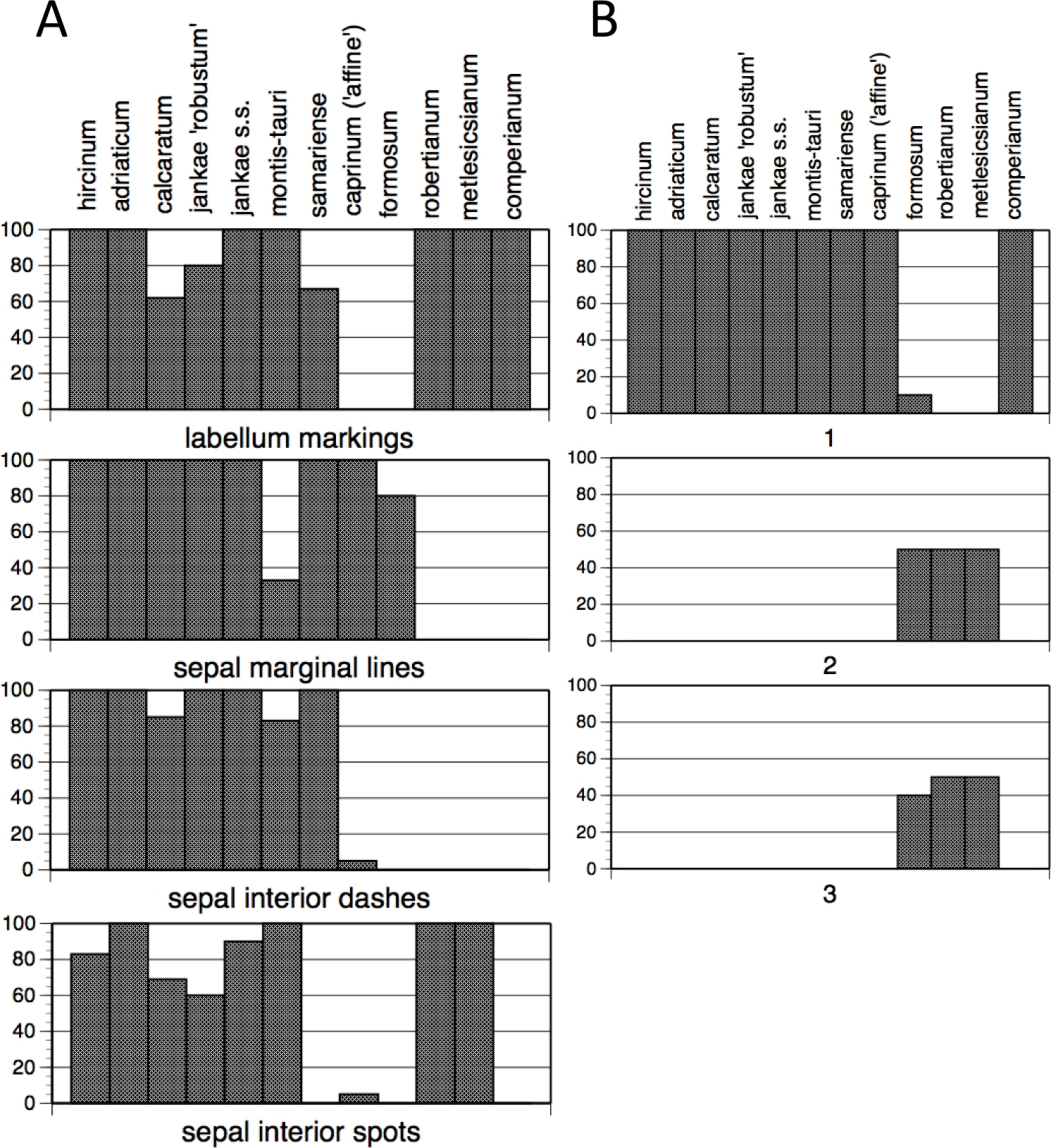
Histograms showing frequencies of (A) flower markings and (B) lateral sepal position. Character states for (B): 1, incorporated into hood; 2, partially spreading; 3, widely spreading.

**Figure 12:**
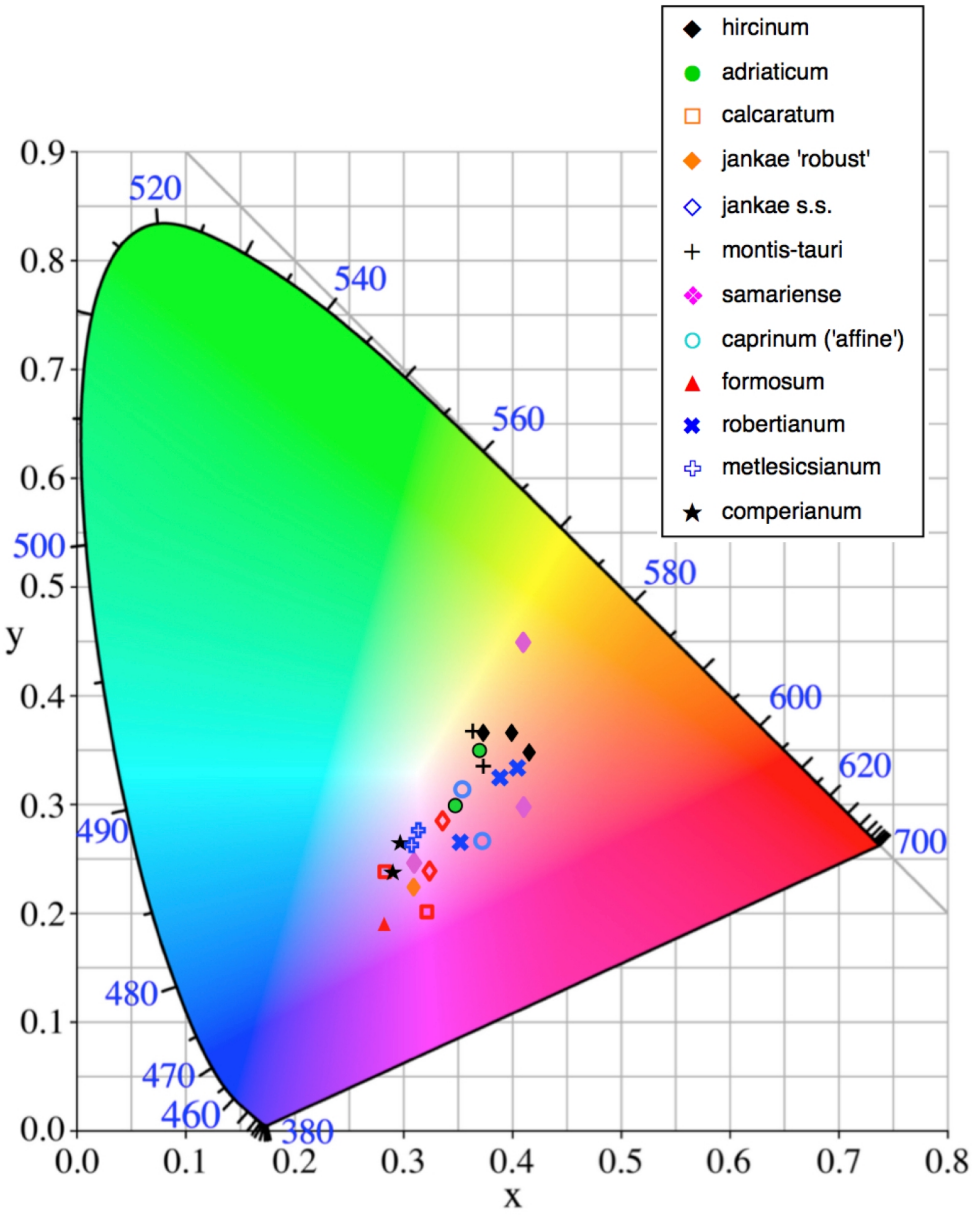
Colour plot for interior labellum margin of the study populations. Population mean values are superimposed onto the CIE colour chart.

**Figure 13:**
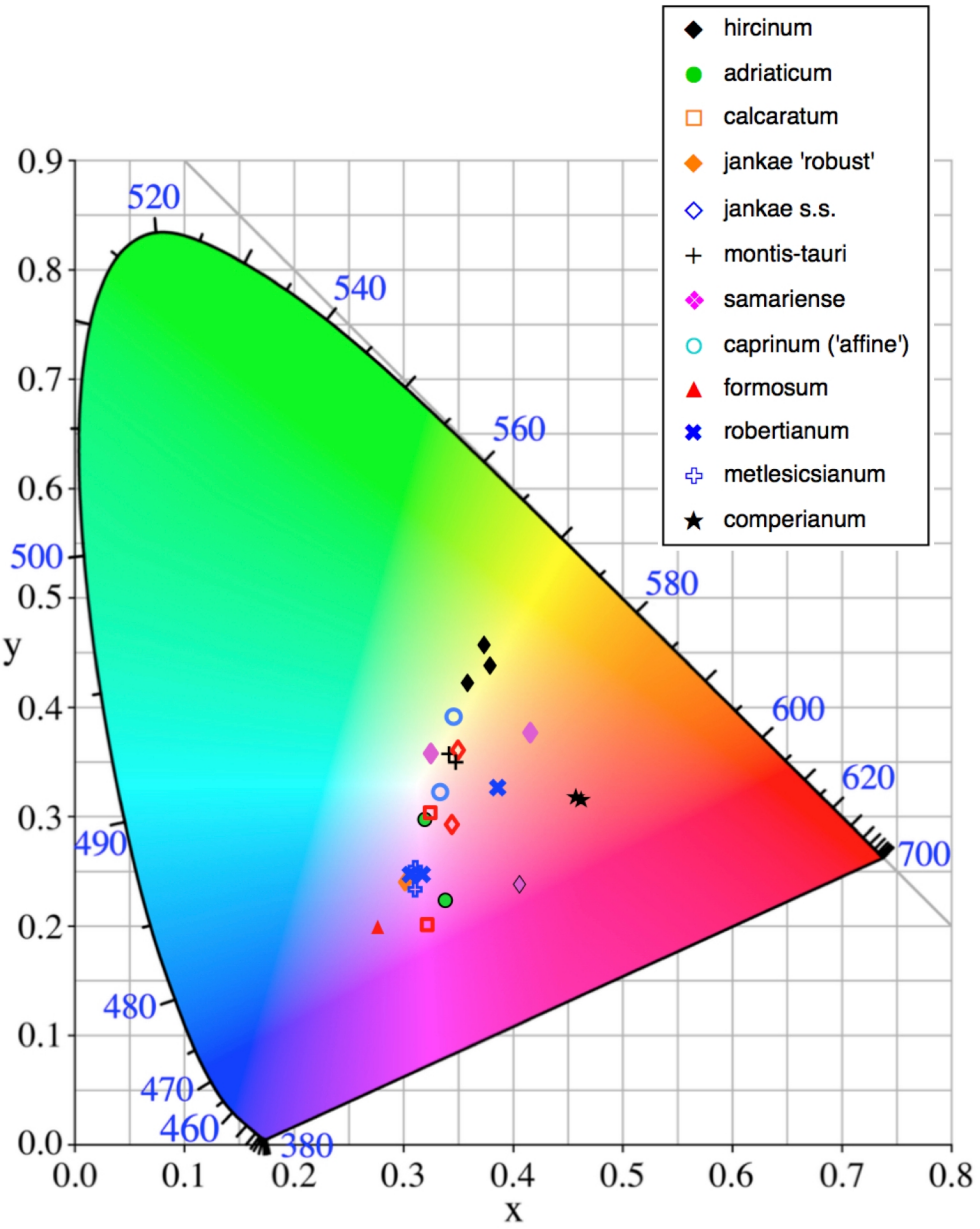
Colour plot for exterior of sepals of the study populations. Population mean values are superimposed onto the CIE colour chart.

**Figure 14:**
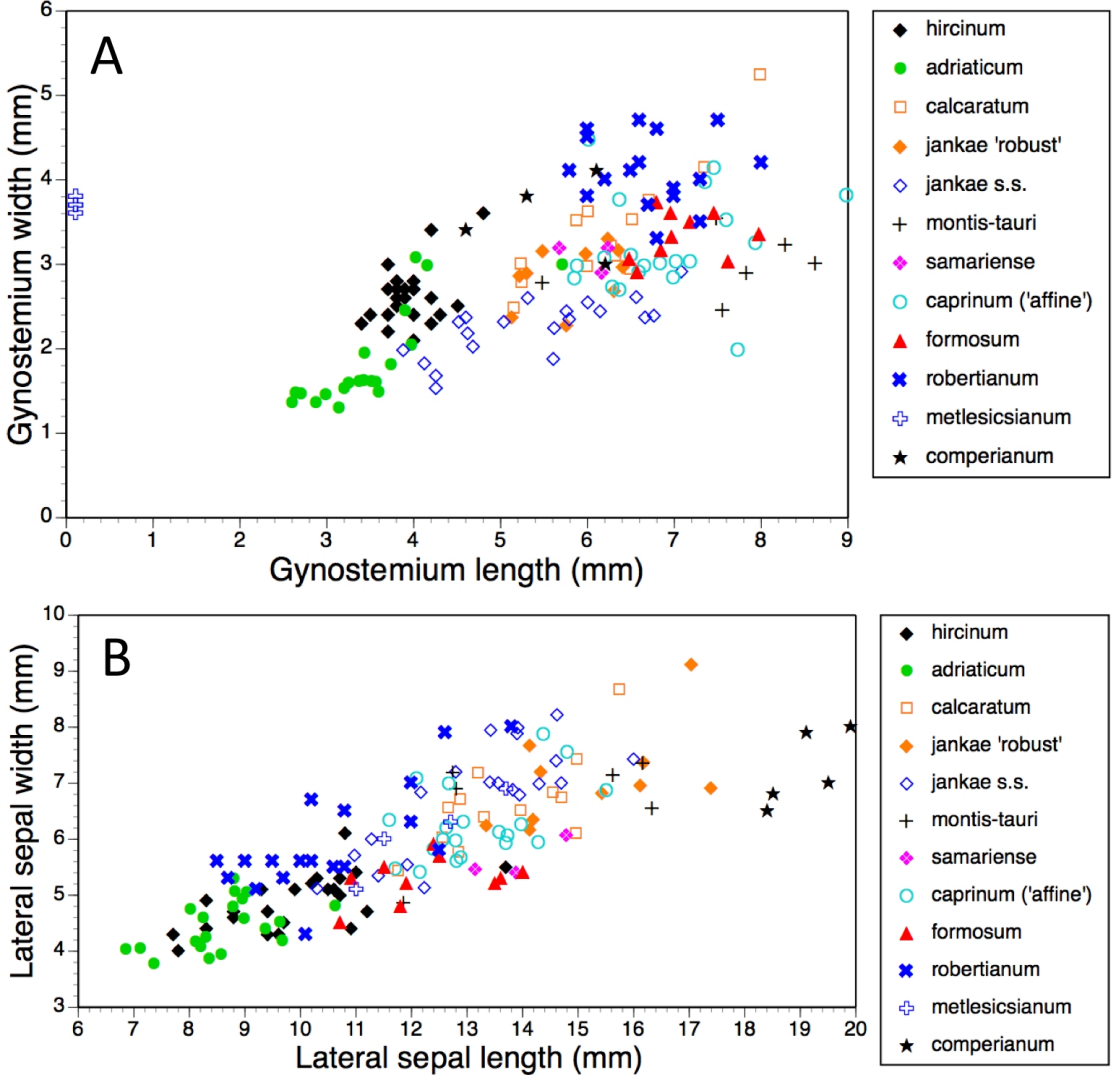
Bivariate scattergrams of (A) gynostemium length versus width and (B) lateral sepal length versus width. Gynostemium length was not measured for *H. metlesicsianum*.

**Figure 15:**
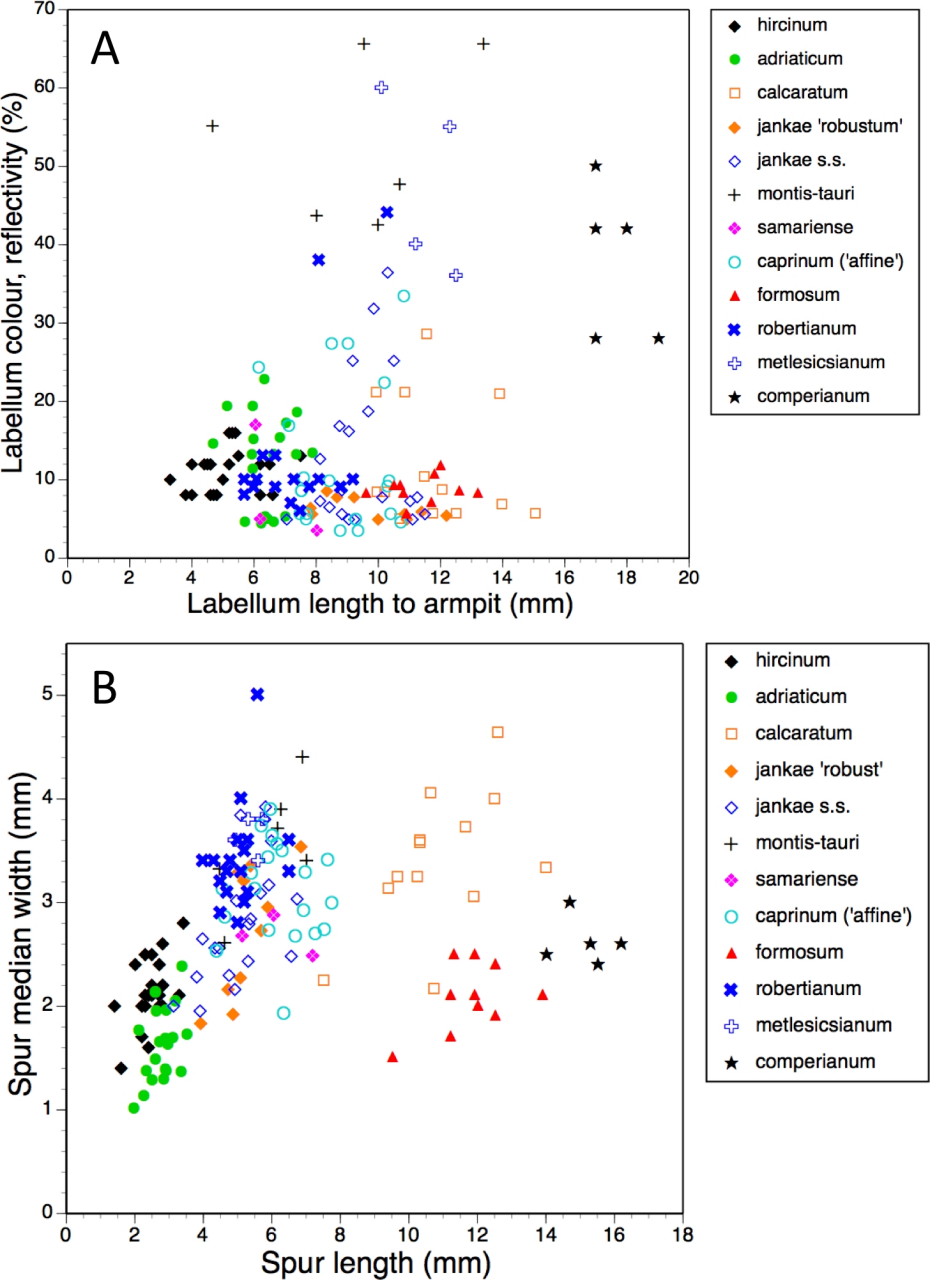
Bivariate scattergrams of (A) labellum length to ‘armpit’ versus labellum colour reflectivity (%) and (B) spur length versus diameter.

**Figure 16:**
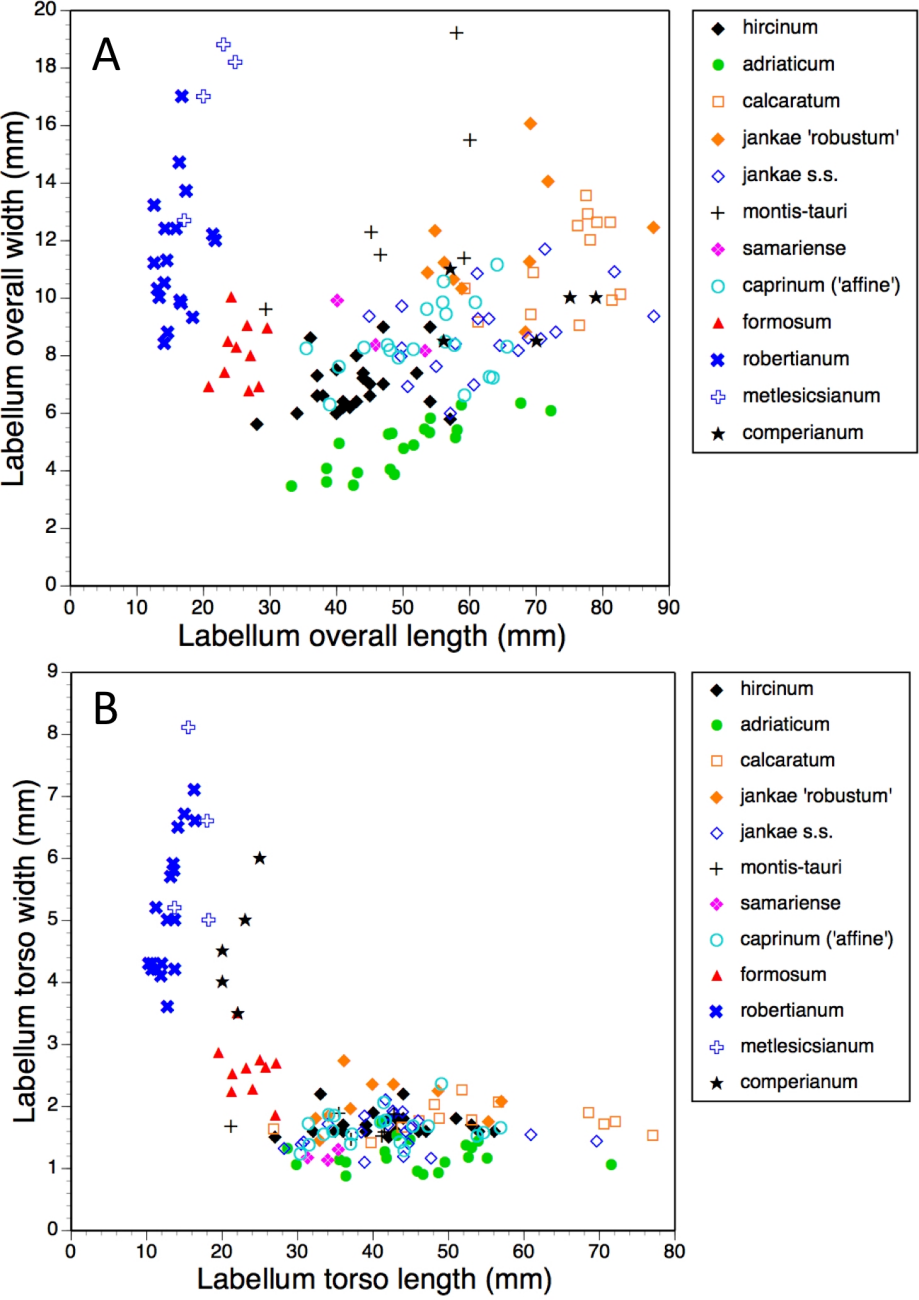
Bivariate scattergrams of (A) labellum overall length versus overall width and (B) labellum ‘torso’ length versus ‘torso’ width.

**Figure 17:**
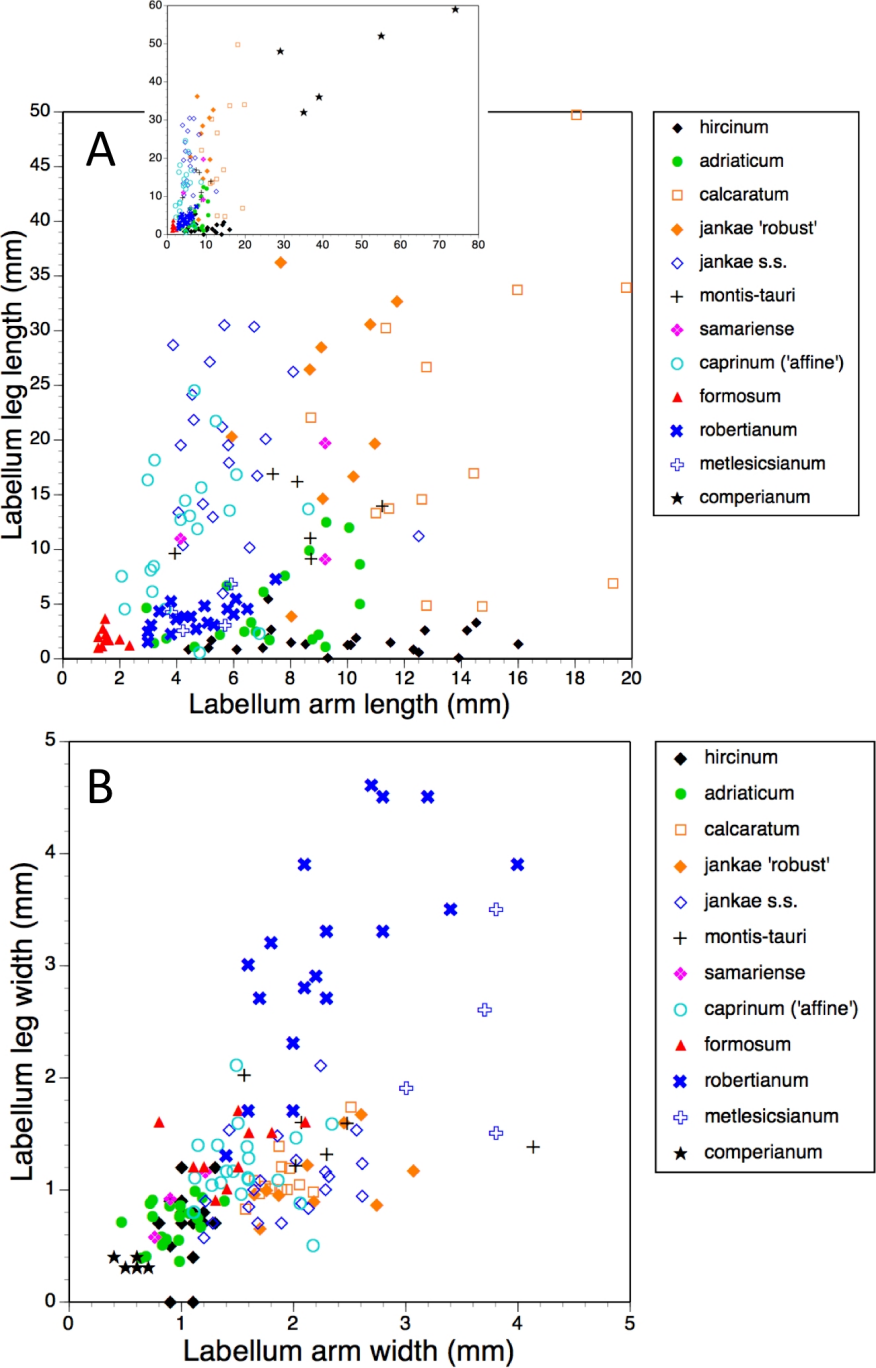
Bivariate scattergrams of (A) arm length versus leg length and (B) arm width versus leg width. The main scattergram in (A) lacks *H. comperianum*, which has exceptionally long arms and is therefore presented in the inset diagram featuring expanded axes.

Understanding of the patterns of morphological similarity depicted in the principal coordinates plots can be further refined through consideration of individual variables, particularly those identified in the multivariate plots as potentially taxonomically sigificant. In total, 25 of the more informative characters are summarised in Figs. 11–17.

#### Pigmentation

The summary of frequencies of characters representing discrete floral markings (Fig. 11A; see also Fig. 7A) makes clear how the presence or absence of each of the four categories of floral marking (discrete spots on the labellum, discrete spots on the interior of the sepals, discrete dashes on the interior of the sepals, peripheral stripes on the exterior of the sepals) interact to diagnose four groups of species. The only species to possess labellum markings but no sepal markings is *H. comperianum* (labellum markings were especially numerous in the Sanctuario population of *comperianum*), whereas *H. robertianum* and *H. metlesicsianum* also possess sepal spots. The only marking type possessed by most individuals of *H. formosum* and *H. caprinum* is the peripheral stripe on the sepals. Most plants of the remaining species possess all four kinds of marking, except that the majority of *H. montis-tauri* lack peripheral stripes. However, the presence of each kind of marking in each taxon cannot be wholly relied upon; only *H. adriaticum*, together with *H. metlesicsianum* and *H. comperianum*, appeared to be fixed for presence or absence of all four categories of marking (Fig. 11A). And given our small sample sizes for these species (only four and five plants, respectively), it is likely that we may simply failed to detect such variants.

Flower colour proved to be challenging to summarise when considering *Himantoglossum* species, as the perianth segments typically had a base colour of yellow-green that then appeared to be ‘overwashed’ with brown, purple or red pigments (Figs. 1–3). Figure 12 shows mean values for two of the three quantified CIE parameters (x and y) that together represent the background colour of the marginal regions of the labellum; Fig. 13 presents comparable data for the exterior surfaces of the sepals.

Most study populations of the same taxon averaged similar labellum colours (Fig. 12), though the three plants of *H. ×samariense* are spread especially widely on the colour grid and the Spanish (Torcal) population of *H. robertianum* exhibited unusually purplish hues that approached those more typical of *H. metlesicsianum*. *Himantoglossum formosum* and *H. comperianum* tended to have labellar margins that are purplish rather than greenish-brown, whereas *H. hircinum*, *H. adriaticum*, *H. montis-tauri* and especially the mainland Greek (Taygeti) *H. ×samariense* leaned toward brownish-yellow.

A wider range of mean values is evident in the equivalent plot for sepal colour (Fig. 13). Although most populations have broadly similar colours in the labellum and sepals, there are exceptions. Most notably, the sepals of *H. comperianum* are unusually red, whereas their labella are purple. The converse is true of two of the three populations of *H. robertianum*, which have purplish sepals associated with browner labella. In addition, sepal colour usefully distinguishes the yellow-green-sepalled *H. hircinum* from the mauve-sepalled Köszeg population of *H. adriaticum* (Fig. 13).

In addition to the precise hue, we can also usefully consider the depth of coloration of the labellum margin (Fig. 14A). Clearly, the majority of taxa are reliably dark flowered (defined here as a reflectivity of incident light of less than 20%). However, *H. caprinum*, *H. jankae s.s.* and *H. calcaratum* show wider spreads of pigmentation density; a minority of individuals of these taxa, together with some plants of *H. comperianum*, have moderately reflective labella (20–40% reflectivity). The remainder of the *H. comperianum* plants, together with all of the *H. montis-tauri* and *H. metlesicsianum* plants measured here and approximately one tenth of the *H. robertianum*, have comparatively pale flowers that are characterised by reflectivities that exceed 40%.

#### Sepal, petal and gynostemium

Gynostemia of *H. adriaticum*, *H. jankae* and *H. montis-tauri* are narrower relative to their length than are those of the remaining taxa (Fig. 14A). Those of *H. adriaticum* and *H. hircinum* are shorter than those of the remaining taxa, though there exists partial overlap in size with the gynostemia of *H. jankae s.s*.

Dimensions of sepals and lateral petals readily distinguish the long-hooded *H. comperianum* from the remaining species (Table 1, Fig. 14B). Although overlapping in length with *H. robertianum*, section *hircinum* has the shortest sepals (excepting the Moroccan population of *H. hircinum*: Bateman et al., 2013), and possesses lateral petals that are both the shortest and narrowest. Also, the sepals of *H. ×samariense* are unusually narrow relative to their length.

Lateral sepal orientation, as perceived relative to the vertical when the flower is viewed from the front, readily distinguishes subgenus *Barlia* plus *H. formosum* (Fig. 11B). They show a mixture of partially and wholly spreading sepals, whereas the remaining taxa reliably incorporate the lateral sepals into the hood (galea) that is consistently formed by the median sepal and lateral petals. The hood in turn completely overhangs the gynostemium.

Long, filiform lateral teeth proved to be ubiquitous on the lateral petals of *H. comperianum*. Shorter, sturdier teeth projected from the petals of the majority of plants of *H. formosum*, as well as from a small minority of plants of each of *H. jankae s.s.*, *H. calcaratum* and *H. hircinum* (Table 1). Such teeth are less frequent across the genus as a whole than was implied by some previous authors (e.g., Delforge, 1999; Sramkó et al., 2014).

**Figure 18:**
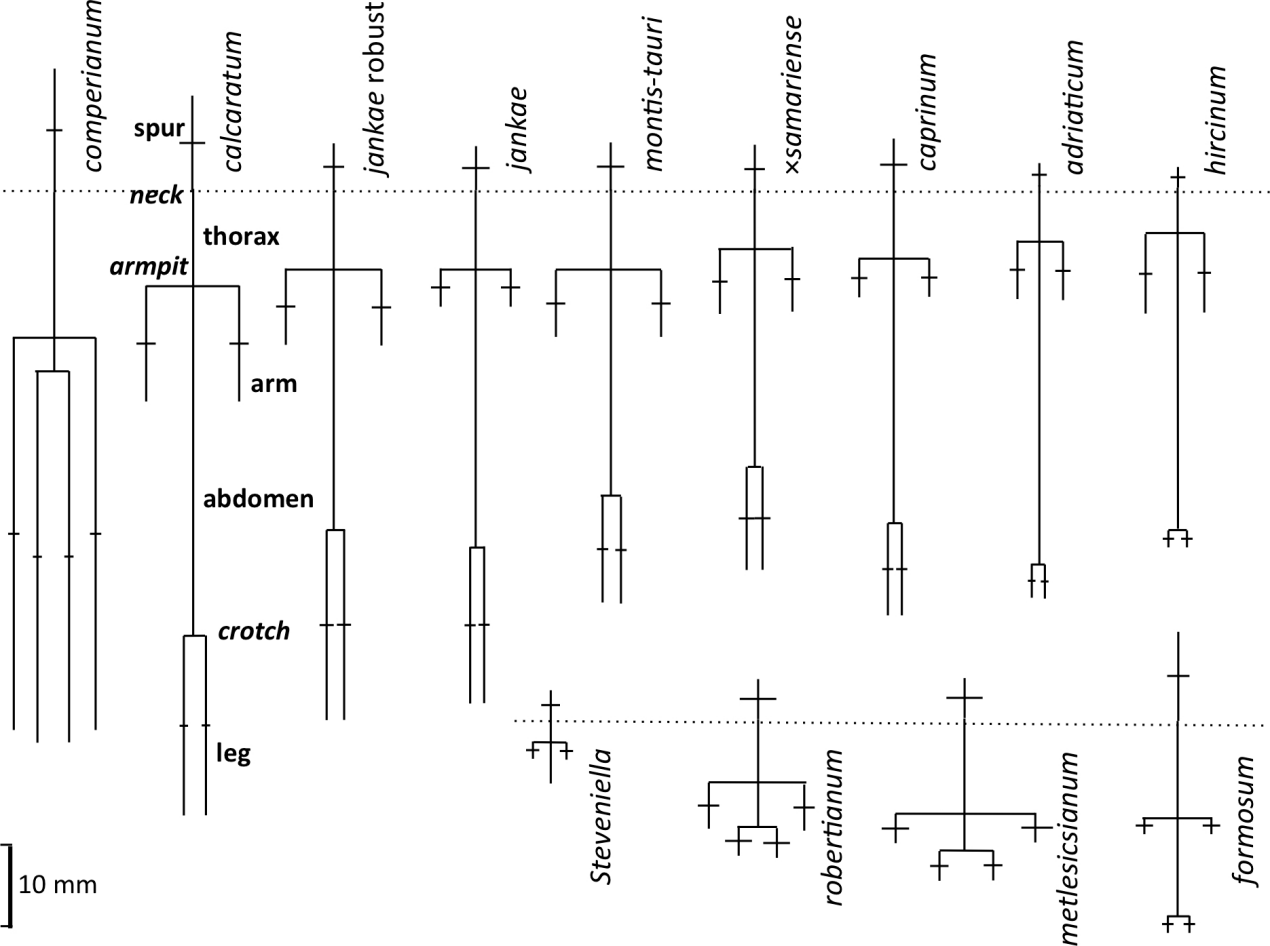
Mean morphologies of the labella of the study taxa, reconstructed from the morphometric dimensions measured. Also shows the anthropomorphic terminology adopted to describe contrasting regions of the labellum.

#### Labellum, including spur

Several characters were needed to represent with acceptable accuracy the unusually complex labellum shape of *Himantoglossum s.l.* species (Figs. 15A, 16–18).

The plot of maximum length versus width of the labellum (Fig. 16A) alone is sufficient to distinguish several of the study species. Comparatively short, broad labella characterise subgenus *Barlia*, which has an especially high width : length ratio and incurs a greater coefficient of variation for width than for length (mean width is greater for *H. metlesicsianum* than for *H. robertianum*). In contrast, the remaining taxa have labella that are much longer than wide and greater coefficients of variation for length than for width. Furthermore, section *caprinum* can achieve greater mean and maximum lengths than can section *hircinum*. The width : length ratio is greatest for *H. montis-tauri* and least for *H. adriaticum*. Individuals of *H. formosum* form a fairly compact, intermediate cluster.

A broadly similar pattern is evident in labellum torso dimensions (Fig. 16B); species of subgenus *Barlia* and subgenus *Himantoglossum* differ radically in length : width ratios, *H. formosum* occupying a position intermediate between them. The main exception is *H. comperianum*, which is long overall (Fig. 16A) but has a comparatively short torso, of similar length of *H. formosum* and similar width to *H. robertianum* (Figs. 16B and 18). Shoulders are reliably narrow in section *hircinum*.

The length of the ‘thorax’—the proximal portion of the labellum that stretches from the spur entrance (‘neck’) to the ‘armpit’ (Fig. 6)—is greatly expanded in *H. comperianum* relative to the other species (Figs. 15A and 18), which may be the reason that it lacks the three-dimensional ‘crenulae’ that characterise the shoulders of all other species in the genus. Although present, these marginal irregularities are larger—and therefore fewer in number—in subgenus *robertianum*. The ‘thorax’ is also comparatively long in *H. formosum* and *H. metlesicsianum* (which exceeds *H. robertianum* in this character), whereas it is short in *H. adriaticum* and especially in *H. hircinum*. The spread of values for this character is especially wide within *H. montis-tauri*.

The main plot of labellum arm versus leg length (Fig. 17A) shows that arms are shortest in *H. formosum* and, to a lesser degree, in the *robertianum* group. The smaller inset diagram shows clearly that the legs of *H. comperianum* are on average longer, and the arms much longer, than those of any other *Himantoglossum* species. Variation in arm length is great within most species, incurring remarkably large coefficients of variation. *Himantoglossum hircinum* and *H. calcaratum* are capable of generating longer arms than the remaining species. Even more striking variation surrounds leg length in most taxa of the *hircinum–caprinum* clade, though not in *H. hircinum* itself (Figs. 17A and 18). Indeed, 11% of the *H. hircinum* plants studied lacked legs altogether, the central labellar lobe being entire rather than apically notched into the characteristic leg-like ‘lobules’. Legs exceeding 5 mm in length form a great majority of most other taxa in the aggregate, notably in the largest-flowered taxa—*H. calcaratum* and *H. jankae* ‘robust’ (Fig. 18). The two populations of *H. adriaticum* differ significantly in this character; those from Köszeg have legs that are considerably shorter than those from Nyirád (mean values 1.8 versus 7.5 mm), thus being more comparable in size with those of *H. hircinum*. Arms are considerably longer than legs in most individuals of section *hircinum* (Fig. 18).

Limb widths (Figs. 17B and 18) readily separate the filiform elongations of *H. comperianum* labella from the wide-armed and especially wide-legged subgenus *Barlia*. In between these two extremes, section *hircinum* plus *H. ×samariense* tend to have narrower limbs than do either section *caprinum* or *H. formosum.* Only 32% of subgenus *Barlia*, together with a single plant of *H. hircinum*, possessed small fifth lobes (‘tails’) located between the legs in the ‘crotch’ of the labellum.

Spur dimensions (Fig. 15B) are also highly diagnostic. In particular, *H. formosum* and *H. calcaratum* have long spurs (those of *H. calcaratum* being broader than those of *H. formosum*, comparable in width with spurs of *H. montis-tauri* and subgenus *Barlia*) and *H. comperianum* has even longer spurs; those of both *H. comperianum* and *H. formosum* typically exceed 75% of the length of the corresponding ovary. Spurs of section *hircinum* are shortest, and within that section, those of *H. adriaticum* tend to be even narrower than those of *H. hircinum*. Greater length appears to permit greater downward curvature of the spur, most notably in *H. comperianum* (Table 2).

Recording the approximate angle subtended by the labellum relative to the stem showed that *H. ×samariense* possesses the most outwardly projecting flowers, whereas in contrast, those of *H. metlesicsianum* are held even closer to the vertical than are those of *H. robertianum*. Also, relative to the attitude of the torso, the minute arms of *H. formosum* project forward, the more substantial arms of *H. metlesicsianum* are borne in approximately the same plane as the torso, but those of all other species (including *H. robertianum*) usually recurve—most strongly so in *H. montis-tauri* (Table 2).

#### Vegetative organs

Unusually for a morphometric study of European orchids, vegetative vigour here plays a comparatively minor role in providing highly variable, and thus potentially taxonomically diagnostic, characters. Moreover, only occasionally do highs and lows in the number and sizes of various vegetative organs strongly co-vary. Hence, vegetative characters are not explored in detail in the present text.

In summary, subgenus *Barlia* have the most robust stems, though they are matched in this character by the Ifrane population of *H. hircinum*. *Himantoglossum caprinum*, *H. ×samariense*, *H. montis-tauri* and especially *H. comperianum* tend to have fewer flowers, whereas inflorescences are densest in subgenus *Barlia* and in *H. hircinum*.

Total leaf numbers are comparatively low in subgenera *Comperia* and *Barlia*, and also in *H. montis-tauri* and *H. ×samariense*. Both subgenus *Barlia* and the relevant members of subgenus *Himantoglossum* appear to compensate in other ways for this potential deficiency in photosynthetic surface area; subgenus *Barlia* produces comparatively large leaves, whereas both *H. montis-tauri* and *H. ×samariense* produce comparatively large bracts.

## Discussion

### Contrast between morphometric analyses of individual plants versus population means

Focusing on population means inevitably reduces the (often considerable) impact of ontogenetic variation among individuals, particularly in vegetative characters such as plant height, flower number, and leaf number and dimensions (e.g., Bateman & Denholm, 1989; Bateman, 2001). Consequently, multivariate plots based on mean values reliably represent a larger percentage of the total variation than do equivalent plots for individuals, as the dimensionality of the data has inevitably been reduced. In contrast, most ecophenotypic influences would affect entire populations, and thus be fully reflected in the resulting population mean values. However, under some circumstances, means can over-simplify individual-level variation, most notably in situations where one or more of the variables is multi-modal (e.g., a population consisting of half white-flowered individuals and half red-flowered individuals would score on CIE colour coordinates as averaging darkish pink—a condition actually found in none of the plants present in the population). Lastly, it is highly desirable that sets of population mean values should each reflect at least several individuals, in order to avoid the negative effects of exaggerated sampling errors.

**Figure 19:**
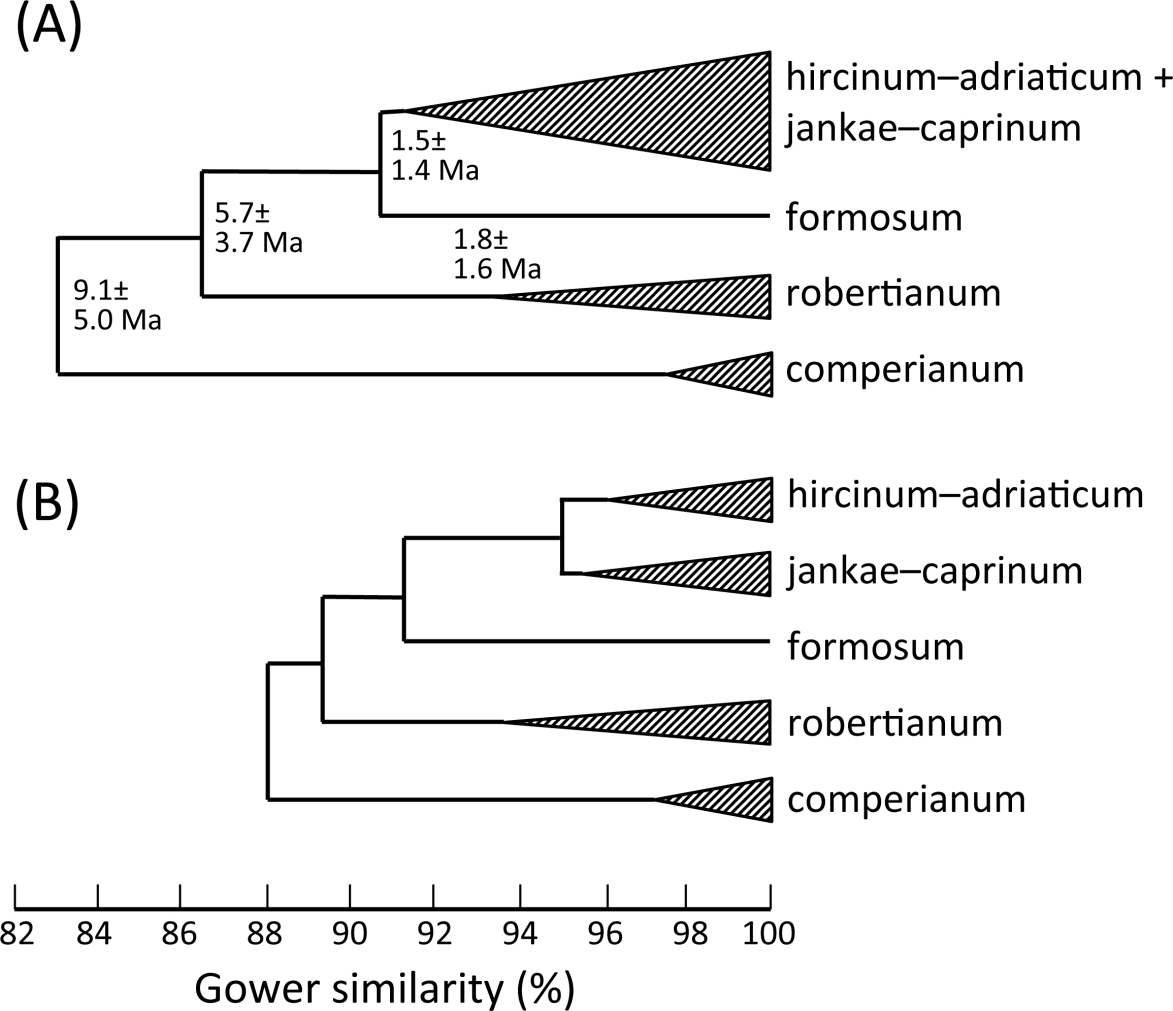
Morphology-based dendrograms simplified to the five major groups within *Himantoglossum s.l.*, based on (A) all characters and (B) pigmentation characters omitted. Internal nodes bear lineage divergence dates estimated via a molecular clock approach by Sramkó et al. (2014, their Fig. 8).

**Figure 20:**
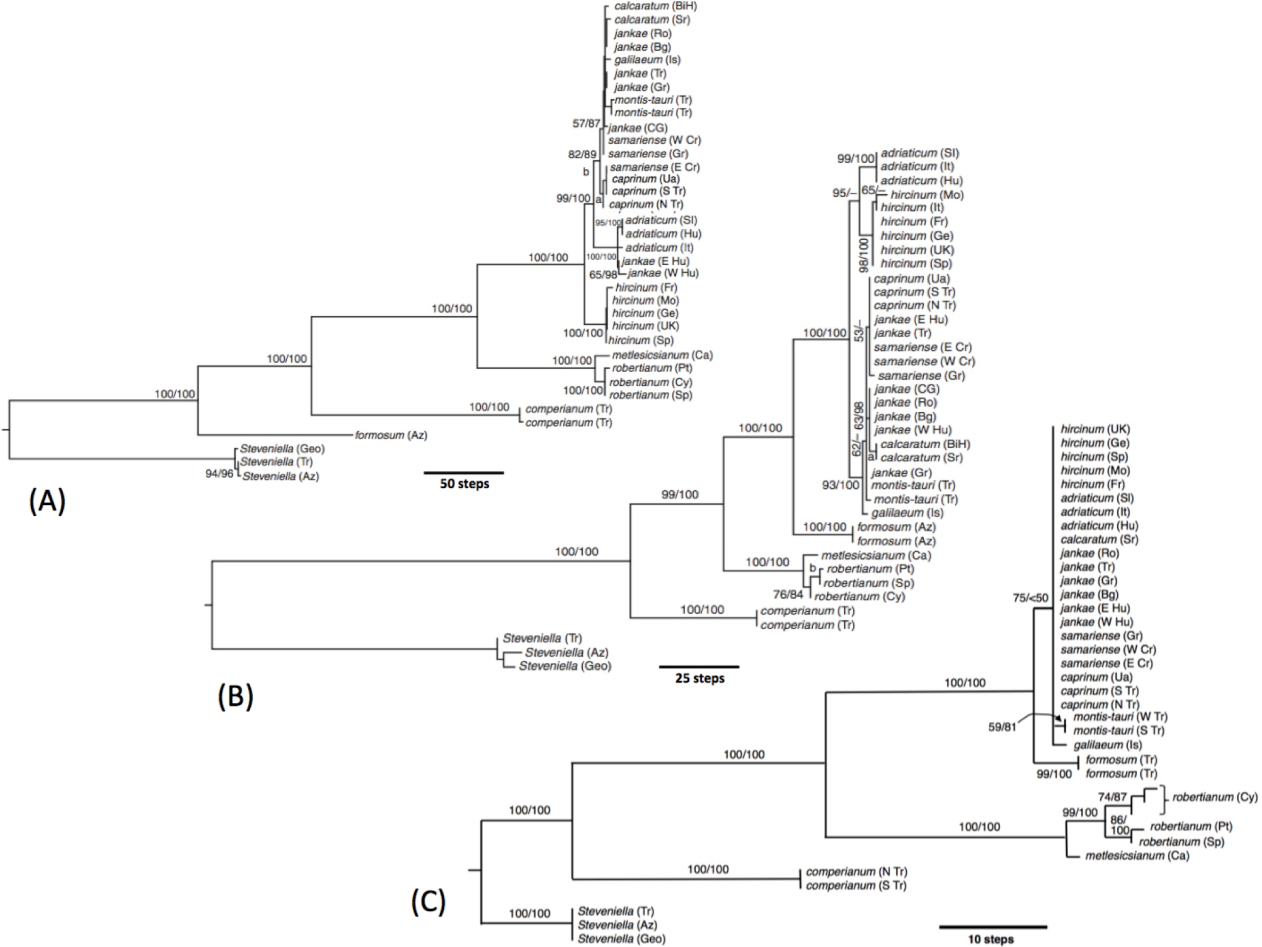
Molecular phylogenies of *Himantoglossum s.l.* (A) Low-copy nuclear gene *LEAFY*. (B) Three fast-mutating plastid regions. (C) nrITS. Abbreviated countries: Az, Azerbaijan; Bg, Bulgaria; BiH, Bosnia-Herzegovina; Ca, Canary Islands; Cr, Crete; Ua, Crimea; Cy, Cyprus; Fr, France; Geo, Georgia; Ge, Germany; Gr, Greece; Hu, Hungary; Is, Israel; CG, Montenegro; Mo, Morocco; Pt, Portugal; Ro, Romania; Sl, Slovenia; Sp, Spain; Sr, Serbia; Tr, Turkey; UK, United Kingdom.

Given these potential complicating factors, it is unsurprising that some significant differences of emphasis are evident here between the morphometric multivariate analyses for individual plants (Figs. 7 and 9) versus those for population mean values (Figs. 8 and 10). These discrepancies at least partly reflect comparatively high dimensionality in the data; in particular, there is limited correlation (either positive or negative) among suites of characters reflecting plant size, flower size, labellum shape and flower colour. The net result is that *H. comperianum* appears more distinct from the other species in analyses of populations versus those of individuals. Much of the taxonomic overlap evident in the principal coordinates plots of individual plants between *H. hircinum* and *H. adriaticum*, and among members of the *H. jankae–caprinum* group, is likely to reflect ontogenetic and/or ecophenotypic differences. And the unfortunate fact that the three populations of *H.* ×*samariense* are each represented only by a single individual undoubtedly at least partly explains their failure to group in the population-level plot for all characters (Fig. 10A).

### Congruence between morphological and molecular data

Although most studies that compare phylogenetic trees effectively synonymise ‘congruence’ only with tree topologies, we are equally interested in exploring relative branch lengths (i.e., degrees of divergence) when comparing the results of our morphological (Figs. 7–19) and molecular (Fig. 20) studies of the *Himantoglossum* clade.

**Figure 21:**
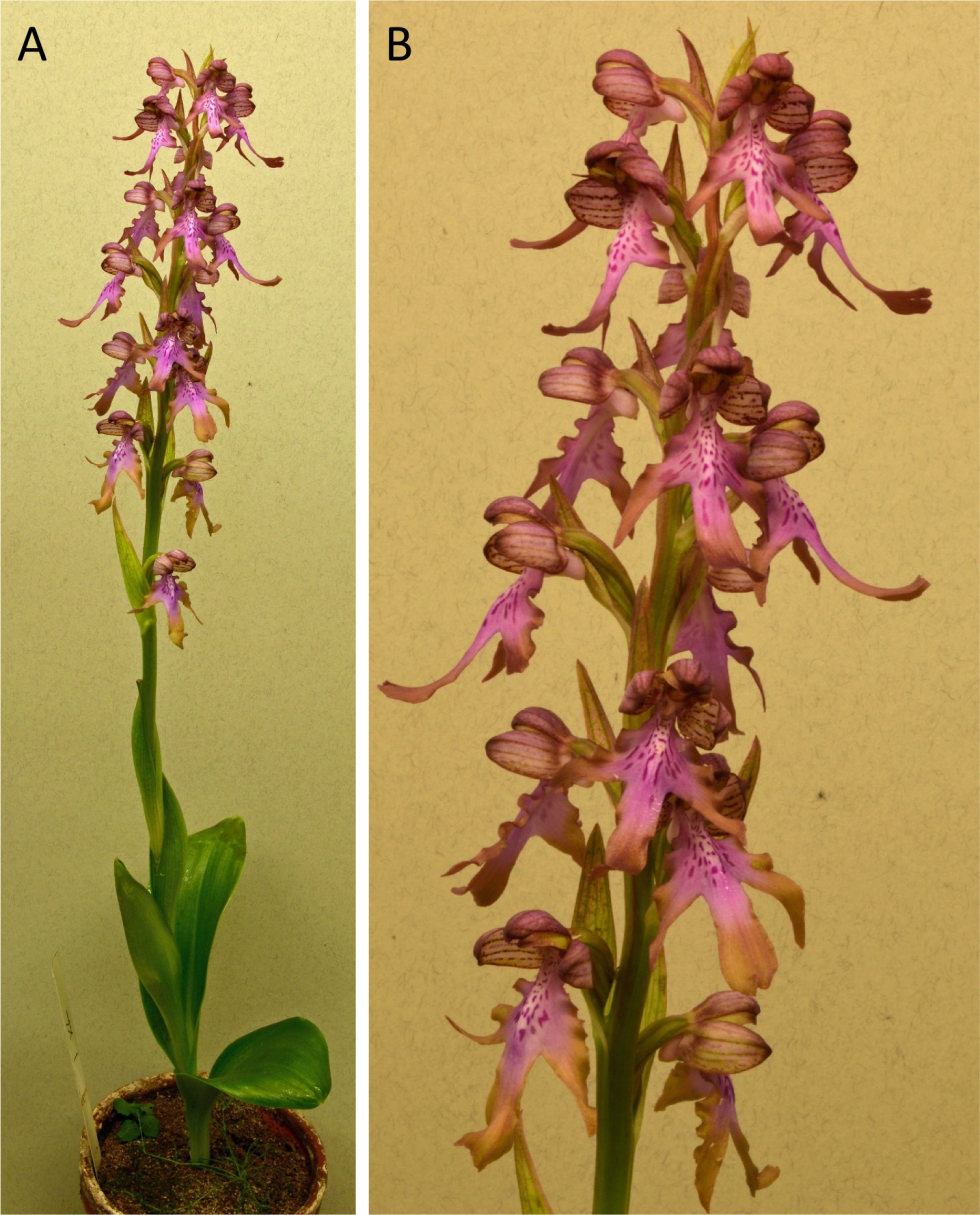
Artificial hybrid of *H. jankae* × *H. robertianum*. Images courtesy of Svante Malmgren.

In our molecular study (Sramkó et al., 2014) we observed two contrasting levels of divergence: (1) strong divergence (irrespective of genic region sequenced) that separated four major groups, and (2) much weaker divergence observed within those four groups. The four groups were, listed in presumed order of divergence, *H. comperianum* (formerly the monotypic genus *Comperia*), *H. robertianum* plus *H. metlesicsianum* (formerly viewed by most observers as a monotypic or near-monotypic genus, *Barlia*), *H. formosum*, and the remaining named taxa that together form the more problematic *hircinum–caprinum* clade. The molecular trees derived respectively from the low-copy nuclear gene *LEAFY*, three concatenated plastid regions, and the high-copy nuclear ribosomal region ITS all yielded broadly similar topologies and branch lengths between these four groups—the three branches separating the divergences of these groups generally being of approximately equal lengths within each of the three trees summarised in Fig. 21.

The main topological uncertainty was caused by *H. formosum*, which (surprisingly) was placed below subgenus C*omperia* and subgenus *Barlia* in the *LEAFY* tree but (more credibly) appeared above these groups in the other two molecular trees. Moreover, *H. formosum* was separated from the *hircinum–caprinum* clade by a comparatively short branch in the ITS tree relative to the corresponding branch in the plastid tree. Sramkó et al. (2014) rejected the early divergence of *H. formosum* implied by the *LEAFY* tree but were unable to strongly advocate any mechanistic explanation for this startling topological incongruence. The most likely explanation is loss from *H. formosum* of the particular copy of *LEAFY* that was sequenced for the remaining species. Indeed, one of us (RMB) has gained the impression that low-copy nuclear genes such as *LEAFY* often yield topologies that (most commonly at comparatively deep nodes) diverge (both profoundly and improbably) from, and hence are less reliable than, both plastid and ribosomal nuclear data derived from those same sampled individuals (*contra* Schlüter et al., 2007).

That opinion is supported by the present morphometric analyses, which reliably show *H. formosum* to be more similar in overall morphology to the *hircinum–caprinum* clade than to subgenus *Comperia* or subgenus *Barlia*, irrespective of whether the analysis is performed at the demographic level of individual plants (Fig. 7) or population means (Fig. 8). The level of morphological disparity shown by *H. formosum* best fits the ITS tree when all morphological characters are included (Fig. 19A), this species being most similar to, but nonetheless distinct from, the *hircinum–caprinum* clade. However, when pigmentation characters are omitted, the revised morphological tree most closely resembles the plastid tree, the branch subtending the *hircinum–caprinum* clade being proportionately longer (Fig. 19B) and thus suggesting an earlier divergence of *H. formosum*. In both of these morphological analyses, *H. formosum* diverges mid-way between subgenus C*omperia* and subgenus *Barlia*, thus mirroring the topology of the plastid tree (Fig. 20B).

When considered at the level of individual characters, *H. formosum* combines features typical of subgenus *Barlia*, such as spreading sepals (Fig. 9B), with features more typical of the *hircinum–caprinum* group, such as possession by the sepals of a marginal stripe (Fig. 11A). *Himantoglossum formosum* is intermediate between the two groups in relative (though not absolute) labellum dimensions (Figs. 16 and 18), but also possesses some more unusual features such as comparatively long, narrow spurs (Fig. 15B), exceptionally short labellar limbs (Figs. 17A and 18), and floral anthocyanins that collectively extend toward the bluer end of the purple spectrum (Figs. 1, 12 and 13).

The remaining topological incongruences among the molecular trees (Fig. 20) occur within the less well-resolved *hircinum–caprinum* clade. Most notably, section *hircinum* is undifferentiable from section *caprinum* in the ITS tree, and the two groups are both clearly differentiable and monophyletic in the plastid tree, but *H. adriaticum* is not placed as sister to *H. hircinum* in the *LEAFY* tree, instead being placed within section *caprinum*. The morphometric data provide better discrimination than does the ITS tree and broadly support the topology of the plastid tree. However, when considered at the population level, the multivariate data representing overall similarity are capable of reliably distinguishing between section *hircinum* and section *caprinum* only when pigmentation characters are omitted (cf. Figs. 9A, 10A and 19A versus Figs. 9B, 10B and 19B). In contrast, multivariate analyses based on individual plants (Figs. 7 and 8B) are more successful at distinguishing the comparatively conservative section *hircinum* from the considerably more morphologically variable section *caprinum*.

Consideration of multivariate contributors (Tables 2–5) and individual characters (Figs. 11–18) shows that members of section *hircinum* bear relatively small flowers, characterised by especially small (in particular, short) spurs (Fig. 15B and 18) and gynostemia (Fig. 14A), short ‘thoraxes’ (Figs. 6 and 18) and legs (Fig. 17A), and narrow labellar limbs (Figs. 17B and 18). In contrast, there are no morphological characters that reliably unify section *caprinum*. In theory at least, this observation could be viewed as circumstantial evidence that section *hircinum* is a monophyletic group that originated from within a more morphologically diffuse and ostensibly paraphyletic section *caprinum*.

Overall, the congruence—in terms of both relationships (topology) and degrees of disparity (branch lengths)—is strong between the molecular matrices gathered by Sramkó et al. (2014) and the morphometric matrices that are the primary focus of the present study. This observation not only increases our confidence in the relationships consistently inferred among the taxa but also implies that averaged relative rates of molecular and morphological evolution were broadly similar, despite the fact that first principles suggest that morphological evolution has more likely followed a punctuational pattern. Comparison of the morphological disparities of the major clades (Fig. 19A) with the divergence dates estimated molecularly by Sramkó et al. (2014, their Fig. 8) suggests that morphology in *Himantoglossum* evolves at a rate of approximately 1.2% divergence in Gower similarity per million years, though in this context it is important to note that the total variation encompassed by Gower similarity is entirely dependent on the nature of the underlying data (i.e., it is a relative rather than an absolute measure).

### Reproductive isolation

#### Geographical separation: overview

The *Himantoglossum* clade brings into sharp relief arguably the most serious general problem that besets systematic biology—that of distinguishing between (1) clinal change across contiguous geographic regions that is best viewed as infraspecific variation versus (2) hybrid zones separating two *bona fide* species that are distinguished by substantial, but nonetheless incomplete, reproductive isolation.

As they are currently conceived, all species other than the Canary Island endemic *H. metlesicsianum* abut geographically at least one other species of *Himantoglossum*—only the High Alps wholly lack* Himanoglossum* populations. However, substantial overlap of species distributions is also uncommon within each subgenus, most notably within the *hircinum–caprinum* clade that is comparatively rich in formal taxonomic epithets (exceptions are the two supposed regional endemics of subgenus *Himantoglossum*: *H. calcaratum* within the broader territory of *H. jankae s.l.*, and *H. montis-tauri* within the broader territory of *H. caprinum*: Fig. 5). This largely jigsaw-like biogeographic arrangement of putative species is consistent with taxonomic partitioning of a morphological (and thus potentially a genetic) continuum. The fact that the morphometric ordinations substantially reconstruct the west–east distribution of the sampled populations (Figs. 7 and 8)—most notably within subgenus *Himantoglossum*—across *ca* 65° of latitude (Fig. 5) obliges us to consider this conundrum particularly seriously. Especially when this pattern is also viewed in the context of the rarity of reports of natural hybrids among the taxa within subgenus *Himantoglossum*.

In his study of the dimensions of flower parts of Greek populations of the *jankae–caprinum* clade, Tsiftsis (2016) detected a south-to-north increase in the lengths of labellum, spur and ovary (but not lateral labellum lobes) potentially driven by differences in regional climates. Although populations from Lesvos and the Peloponnese attributed by some authors to *H. caprinum* (or even occasionally to *H. montis-tauri*) possessed on average smaller flowers than those studied further north, the apparently clinal nature of these size differences caused Tsiftsis (2016) to argue that, among populations of the *H. jankae* group in Greece, there was no clear taxonomic structure. He therefore concluded that Greece supports only a single (albeit highly morphologically variable) species within section *caprinum*. This would be a defensible position to adopt if taxonomic decisions were to be based entirely on morphological data rather than involve reciprocal illumination with genetic data.

#### Cytology and ploidy change

Chromosomal data for members of the genus *Himantoglossum* are patchy and, for some species (notably *H. comperianum*), appear contradictory (reviewed by Bateman et al., 2003). The best-characterised karyotypes (D’Emerico et al., 1990; D’Emerico, Bianco & Medagli, 1993; D’Emerico, Pignone & Scrugli, 2001) show *H. adriaticum* and arguably also *H. hircinum* to be typical members of the 2*n* = 36 clade of subtribe Orchidinae—a monophyletic group that was first recognised by Pridgeon et al. (1997) and whose integrity was most recently reinforced by the study of Tang et al. (2015).

Unfortunately, data on karyotypes and especially on ploidy levels remain weak across the *Himantoglossum* clade as a whole (Bateman et al., 2003). Polyploidy (both allo- and auto-) has long been known to be rife in the digitate-tubered clade of subtribe Orchidinae that includes *Dactylorhiza* (e.g., Hedrén, Nordström & Bateman, 2011) and *Gymnadenia* (e.g., Trávníček et al., 2012). Although the 2*n* = 36 clade that includes *Himantoglossum* and *Steveniella* presently appears less prone to ploidy change, two ploidy levels have been reported in *H. hircinum* via two unconnected studies, one employing chromosome counts (Bernardos & Amich, 2002) and the other employing flow cytometry (Leitch et al., 2009). Even given these very limited data, it is tempting to speculate whether polyploidy could, for example, be responsible for generating the parapatric populations of *H. hircinum* in Germany that reportedly flower on average three weeks later than the nominate race and were recently formally described as var. *aestivalis* by Kreutz & Steinfeld (2013). Population-level application of flow cytometry would be the simplest and easiest method of surveying *Himantoglossum s.l.* for potential ploidy-change events.

#### Artificial versus natural hybridisation

**Figure 22:**
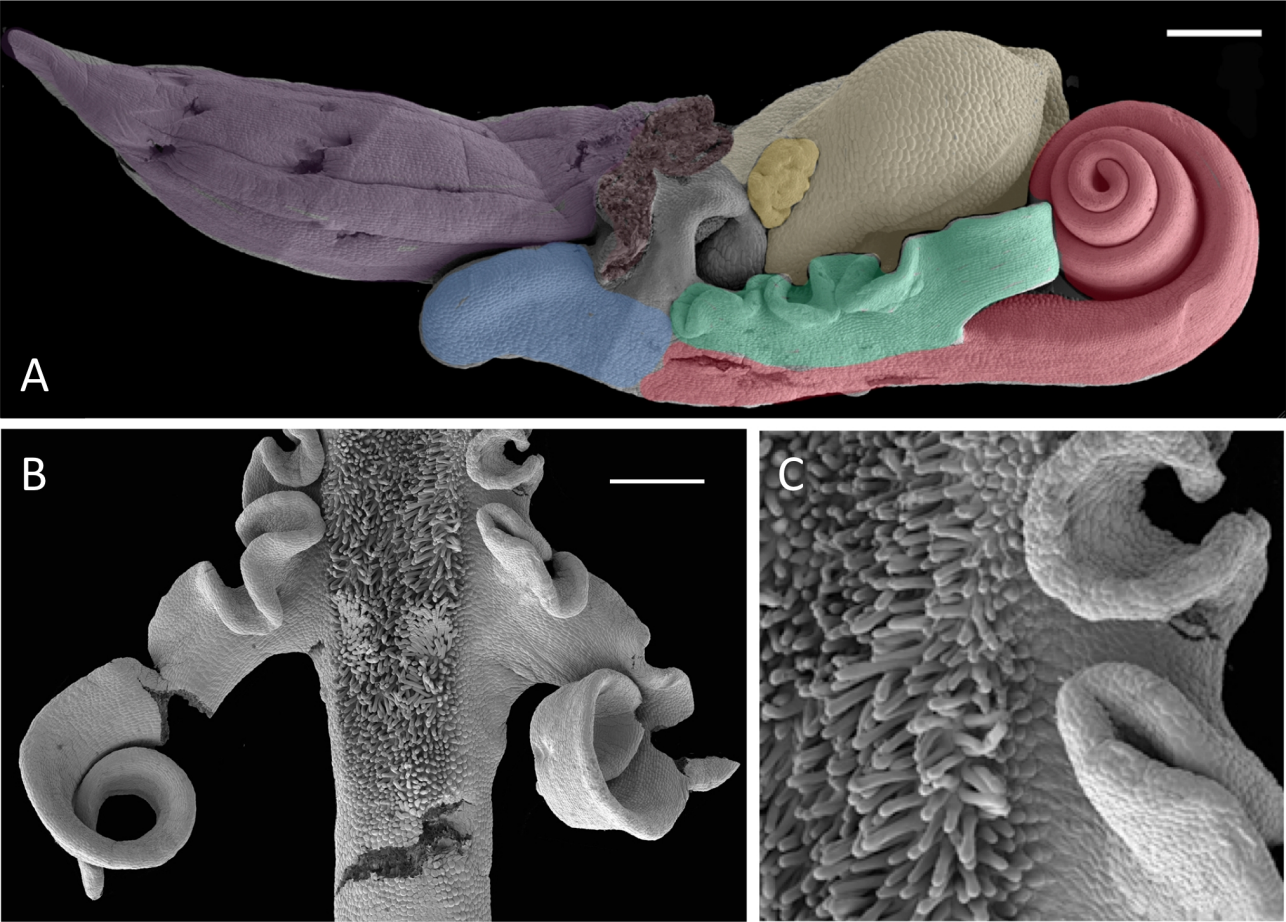
SEM images of *H. hircinum*, showing (A) a lateral view of a late-stage bud, (B) the papillose central region of the labellum of a recently opened flower, and (C) a magnified view of the papillae. In (A), mauve, ovary; grey, base of gynostemium; pale yellow, bursicle and connective; dark yellow, auricles; blue, labellar spur; green, lateral labellar lobes (arms); red, central labellar lobe (torso plus legs). Scale bars: A = 500 µm, B = 250 µm. Images courtesy of Paula Rudall.

Artificial crosses produced by Scopece et al. (2010) between *H. hircinum* and members of other genera of subtribe Orchidinae showed near-complete postzygotic isolation, yielding little if any putatively fertile seed (1.7% with *Serapias cordigera*, none with *Ophrys fuciflora*, and 0.3% with the more phylogenetically distant *Dactylorhiza saccifera*). Artificial hybrids raised by Malmgren & Nyström (2016) between two highly divergent species within *Himantoglosum s.l.*—*H. jankae* and *H. robertianum*—similarly showed reduced degrees of fertility. However, it would be unwise to over-interpret this observation, as the major phenological divergence between these two species meant that it was necessary to freeze the pollinaria of *H. robertianum* for approximately three months until they could be defrosted and applied to the stigma of the captive *H. jankae* ’mother’ plant. The fact that the resulting F1 plants both grew and flowered vigorously (Fig. 22) suggests that intrinsic sterility barriers are at best weak within the group, even when the parents of the primary hybrids are sampled from within different subgenera.

However, if sterility barriers are indeed weak, remarkably few putative examples of recent natural hybridisation within the *Himantoglossum* clade have been reported with confidence. The best-known case—also combining two subgenera—is a few plants on the Aegean island of Lesvos that have been monitored for several years (e.g., Van Lent, 2015) and provide an example of ’wide hybridisation’ between *H. comperianum* and members of section *Caprinum*. The morphology of the plants left little doubt regarding their hybrid origin (Karatzas, 2004). A subsequent molecular investigation of the Lesvos plants, supported by landmark analysis of the labellar outline, not only confirmed their identity but also showed that either *H. caprinum* or its hybrid *H. ×samariense* was their seed parent and *H. comperianum* was their pollen parent (K Hürkan, A Molnár, R Bateman & G Sramkó, unpublished data). This polarity of gene transfer was also demonstrated for two similar hybrid plants found by Molnár and colleagues at Kücükcuker (Samsun) in northeast Turkey.

#### Phenological divergence

Subgenus *Barlia* has undoubtedly acquired strong phenological isolation, its flowering period rarely overlapping with those of other *Himantoglossum* species. Flowering of *H. metlesicsianum* typically extends from December to February, and that of the far more widespread *H. robertianum* from December (in Morocco) to April, depending on latitude and altitude.

In contrast, the remaining species of *Himantoglossum* flower from May to July, the season ending with high-altitude populations of *H. jankae*. Juxtaposed populations of *H. adriaticum* and *H. jankae* in Hungary have flowering periods that overlap but detailed studies have shown that the latter peaks a fortnight later than the former (Molnár, 2011; Biró & Bódis, 2015). *Steveniella*, sister-group of *Himantoglossum*, typically flowers in May. Together, these observations suggest that it is the *robertianum* group that has diverged from the remaining taxa rather than vice versa, its phenology having shifted radically during evolution to achieve a flowering period that is evidently much earlier, even when latitude and altitude are taken into account.

#### Hybrid origin of *H. ×samariense*

We noted previously (Sramkó et al., 2014) that our molecular data strongly indicated a relatively recent hybrid origin for *H. ×samariense*, reinforcing a suggestion made on morphological evidence by some previous authors (Alibertis & Alibertis, 1989; Delforge, 1999). The three plants analysed, sampled from populations in S Greece, W Crete and E Crete respectively, diverged from each other in both plastid and especially *LEAFY* sequences. In the case of *LEAFY* data, the E Crete sample of *H. ×samariense* clustered with plants of *H. caprinum* from the Crimea and S Turkey, whereas the W Crete and S Greece samples clustered with the *H. jankae–calcaratum* group (Fig. 20A). In the case of plastid data, all three samples of *H. ×samariense* are clustered with all samples of *H. caprinum* and some samples of *H. jankae s.s.* (but not *calcaratum*); in trees derived from this organellar genome it is the mainland Greek plant that differs slightly from the two remaining *×samariense* plants (Fig. 20B).

When morphology is considered, it is the W Crete plant of *×samariense* that differs from the other two, primarily in lacking labellum markings—a characteristic that this plant shared with most plants of *H. caprinum*, though unlike *caprinum* it did possess interior dashes on its sepals (Fig. 11A). The three *×samariense* plants are placed comparatively close together on the multivariate ordinations (Figs. 7–10), typically occupying the space between the eastern European *H. jankae* and the Turkish *H. caprinum*, as they do in the plots of paired floral dimensions (Figs. 14–17). However, these populations of *×samariense* are not reliably shown as being most similar to each other; the W Crete sample understandably tends to associate with *H. caprinum* in analyses that include the pigmentation characters lacked by all of these plants (Figs. 9 and 10A). The three *×samariense* plants are also especially divergent in both labellum colour and sepal colour (Figs. 12 and 13).

Taken together, these observations suggest that each of the three *H. ×samariense* populations that we studied had a separate, comparatively recent origin through hybridisation between *H. jankae s.l.* and *H. caprinum*. Unfortunately, the similarity of their plastid genomes to at least some populations of each putative parental species means that we cannot presently distinguish the seed-parent from the pollen-parent in any of the three cases (Fig. 20B). Also, *×samariense* plants are on average smaller-bodied and fewer-flowered than other members of the *jankae–caprinum* clade; they certainly do not show evidence of hybrid vigour.

#### Gene flow between *H. hircinum* and *H. adriaticum*

Within subgenus *Himantoglossum*, *H. hircinum* has the most westerly distribution, which centres on France and extends further longitudinally than it does latitudinally. Moreover, its eastern contact zone with *H. adriaticum* is severely constrained by the presence of the Alps (Fig. 5). However, the opportunity for gene flow between these species is increased by the presence of disjunct populations of *H. hircinum* reputed to occur in Sicily and the southernmost quarter of mainland Italy, where morphologically intermediate populations have been reported (J Bódis, pers. comm., 2016).

These disjunct populations attributed to *H. hircinum* merit more detailed study. The single population from southern Italy that was analysed by Pfeifer et al. (2009) deviated substantially in AFLP spectra from all other populations, while Bateman et al. (2013) reported equally deviant ITS ribotypes in multiple samples of *H. hircinum* analysed from Sicily (admittedly, a further ribotype similarly characteristic of both Sicily and southern Italy was also found in northern France and southeast England). Unfortunately, we do not yet possess morphometric data from Italy to allow comparison with those datasets obtained by us from the core distribution of *H. hircinum* further west.

#### Gene flow between *H. adriaticum* and *H. jankae*

Also in need of explanation is the fact that all *H. adriaticum* plants are shown as sister-group to all *H. hircinum* plants in the plastid tree but in the *LEAFY* tree they cluster with two Hungarian plants of *H. jankae*. Within Hungary (and elsewhere in eastern Europe), a sharp divide has been documented between *H. adriaticum* to the west and *H. jankae* to the east, the two species being separated west of Budapest by as little as 20 km (Molnár, 2011). There are two possible interpretations of these observations: either (1) *H. adriaticum* may currently occasionally hybridise with *H. jankae* along a north–south oriented hybrid zone, or (2) *H. adriaticum* itself may be an older, stabilised hybrid that formed between *H. jankae* and *H. hircinum*, though in this case, theory would have predicted that plastid sequences of *H. adriaticum* would nest within a paraphyletic *H. hircinum* rather than the arrangement seen in Fig. 20B where the plastids of both species are represented as mutually monophyletic.

The present morphological data reliably place *H. adriaticum* as close to *H. hircinum* but with a marginally more extreme phenotype; it shows little potential evidence of any morphological features that are likely to have been inherited from *H. jankae* or its relatives; the two species exhibit noteworthy similarities only in the small width-to-length ratio of their gynostemia (Fig. 14A) and in each possessing only a minority of plants with labellar legs exceeding 5 mm (Fig. 17A). Thus, recent gene flow from *H. adriaticum* into *H. jankae* appears to be the more likely hypothesis to explain the incongruence observed between the *LEAFY* tree and the plastid tree.

#### Gene flow within the *H. jankae–caprinum* clade

Moving eastward, it is section *caprinum* that presents the most serious challenge, both to circumscription of specific/infraspecific taxa (cf. Gölz & Reinhard, 1986; Delforge, 1999; Baumann & Baumann, 2005; Baumann & Lorenz, 2005a; Baumann & Lorenz, 2005b; Shifman, 2008; Ponert, 2014; Sramkó et al., 2014; Tsiftsis, 2016) and to confident recognition of hybrids. When two taxa are distinguished by only subtle phenotypic differences and/or character states that are not fixed in all individuals of each taxon, it becomes impossible to reliably identify hybrids between them using morphology. Any identification based on phenotypic characters requires the presence of a clear morphological discontinuity into which any primary hybrids will fall as a result of combining numerous characteristics of both parents (e.g., Bateman & Denholm, 1983; Bateman, Smith & Fay, 2008).

Admittedly, by definition, hybrids between species that are genuinely morphologically cryptic will be identifiable only via appropriate genetic analyses. In this context, application of a combination of morphometric landmark analysis of labellum shape and *LEAFY* sequencing to taxonomically controversial populations of section *caprinum* on the Aegean island of Lesvos strongly indicate that they reflect hybridogenic origin between members of the *H. jankae* and *H. caprinum* groups (K Hürkan et al., unpublished data)—an explanation that might also apply to morphologically similar (and equally controversial) populations located on the western side of the Aegean in the Taygetos Mountains of the Peloponnese (cf. Petrou, Petrou & Giannakoulias, 2011; Tsiftsis, 2016). Ironically, these potentially hybridogenic plants in turn provided one parent of the hybrids between subgenus *Himantoglossum* and subgenus *Comperia* found on Lesvos.

Considering first the DNA-based datasets, both the plastid and *LEAFY* trees suggest (albeit with limited statistical support) that the other named taxa collectively forming section *caprinum* all emerged from within the comparatively widespread *H. jankae*—a hypothesis that is neither supported nor refuted by the comparatively undiscriminating ITS tree (Fig. 20). The *LEAFY* tree shows both of the putative species that are endemic to Turkey and adjacent regions of Asia Minor—*H. montis-tauri* and *H. caprinum*—to be both monophyletic and derived relative to *H. jankae*. Neither statement applies to the plastid tree, which instead shows *H. calcaratum* (supposedly exclusively Balkan) as being both monophyletic and derived. And lastly, the ITS tree shows *H. montis-tauri* as monophyletic and derived. Thus, within the context of our datasets, the only named taxon within section *caprinum* that wholly lacks molecular autapomorphies relative to *H. jankae s.s.* is *H. jankae* subsp. *robustissimum* from Turkey, a localised taxon originally described by Kreutz (2006).

From a morphological perspective, derivation of the other section *caprinum* taxa from within *H. jankae s.s.* appears feasible; their often subtle differences are multi-dimensional and hence the populations are difficult to resolve into credibly divisible units. This inference is tentatively reinforced by the fact that *H. jankae* has a median overall morphology for subgenus *Himantoglossum* as a whole (Figs. 7–10).

#### Pollination mode and fruit set

Despite occasional assertions to the contrary, no credible evidence has accumulated to suggest that any member of the genus *Himantoglossum* produces nectar (reviewed by Teschner, 1976; Teschner, 1980; Claessens & Kleynen, 2011; Bateman et al., 2013). Although dense papillae evidently occur throughout the interiors of the spurs of both *H. hircinum* and *H. robertianum* (illustrated on pages 262 and 266 of Claessens & Kleynen, 2011)—papillae that are theoretically capable of secreting and/or resorbing nectar—in practice there is a poor positive correlation between the presence in spurs of papillae and production of nectar when they are analysed across subtribe Orchidinae (Bell et al., 2009).

We are not aware of any reports of pseudocopulatory behaviour shown by insects visiting *Himantoglossum*, and fruit-set figures rarely exceed the 80% threshold that constitutes the lower limit typical of autogamous orchids (e.g., Bateman, Sramkó & Rudall, 2015). In fact, fruit-set averages 34.3 ± 20.9% in *H. hircinum*, 28.7 ± 16.1% in the closely related *H. adriaticum*, and 39.6 ± 16.8% in the earlier flowering *H. robertianum* (figures derived by us from 21 individual studies summarised in Appendix 2 of Claessens & Kleynen, 2011). Degradation of the rostellum and/or loss of coherence of pollinia have been reported in *H. robertianum* (Teschner, 1976), but although these phenomena increasingly appear to be widespread among European orchids, they rarely effect self-pollination if they occur only in flowers that have already become senescent. It is therefore reasonable to assume that all members of the genus *Himantoglossum* are pollinated through food-deceit and are dominantly allogamous (cf. Cozzolino & Widmer, 2005; Scopece et al., 2007). The reward-less nature of the flowers is likely to encourage a significant proportion of geitonogamous pollinations, effected while increasingly frustrated visiting insects explore the reliably large, many-flowered inflorescences produced by *Himantoglossum* (Summerhayes, 1951; Kropf & Renner, 2008).

#### Pollinator attractants

Pollinator attraction by orchid flowers typically occurs first through air-disseminated scent, then through vision as the pollinator approaches its target inflorescence, and finally through tactile cues as it lands on its chosen flower.

In the case of *H. hircinum*, the goat-like floral scent has been shown using mass spectroscopy to be a cocktail of alkanoic acids, specifically decenoic acid plus dodecanoic (lauric) acid (Kaiser, 1993). Despite being the closest relative of *H. hircinum*, *H. adriaticum* is said to have a sweeter scent (Vöth, 1990), though it has yet to be analysed biochemically. In contrast, volatile organic compounds released by *H. robertianum* (occasionally said to collectively resemble the scent of hyacinths) are reputedly dominated by monoterpenes, notably pinenes and limonene (Gallego et al., 2012). As already noted, in members of subgenus *Himantoglossum*—at least, in those individuals that bear labellum markings—the epidermal cells that contain the anthocyanins and so delimit the markings occur within a central region of the labellar epidermis that expands outward to form a continuous mat of prominent, densely packed papillae (Fig. 22B). This papillose mat may offer footholds to visiting insects, but more significantly, it may also be an osmophore that is responsible for much of the volatile organic compounds emitted by the plant (see also Vöth, 1990).

Similarly, despite the great variation that we have documented above in floral background colours and discrete markings, exploration of the biochemistry of floral pigments of *Himantoglossum s.l.* has been disappointingly limited. Strack, Busch & Klein (1989) found the flowers of both *H. adriaticum* and *H. robertianum* to be dominated (at least, among those compounds that could be confidently identified) by the evolutionarily labile, cyanin-based compounds serapianin and seranin—compounds that were also shown to be dominant in some other members of the 2*n* = 36 clade of subtribe Orchidinae such as *Anacamptis papilionacea* and *Serapias vomeracea laxiflora*. Surprisingly, *H. metlesicsianum* apparently differed from *H. robertianum* in containing greater relative proportions of orchicyanins (an observation that may actually reflect lower absolute concentrations of serapianin and seranin).

The majority of *Himantoglossum* species have labella where at least the marginal zone, and in some cases all but a small central area, are dominated by a range of brownish hues that are infrequently encountered among European orchids. We suspect that the brownish colour represents the dual presence of green pigment(s) underlying purple anthocyanins, both categories of pigment varying among plants in both relative and absolute densities. If so, the difference in colour between the brownish labellar margins of *H. robertianum* and the pink-purple labellar margins of *H. metlesicsianum* (Fig. 1) would simply reflect the absence from the latter of underlying green pigments. By this logic, the uniformly olive-green labella of *H. montis-tauri* would mean that, unlike other members of the *hircinum–caprinum* clade, it does not express pink-purple pigments diffusely across the labellum, but rather confines them to discrete labellar markings (cf. Fig. 2 vs Fig. 3).

#### Pollinator identity

As summarised by Claessens & Kleynen (2011), most of the pollinator observations among *Himantoglossum* species have been obtained from the usual triumvirate of the three widespread western European species (*robertianum*, *hircinum*, *adriaticum*) and almost exclusively concern bees. Eight species of the solitary bee *Andrena* have been reported as visiting *H. hircinum*, together with representatives of five further bee genera plus the beetle genus *Oedermera* (e.g., Teschner, 1980; Vöth, 1990; Kropf & Renner, 2008). A similar but slightly narrower range of bees are known to visit *H. adriaticum* (Claessens & Kleynen, 2011; Biró et al., 2015), including the social honey bee *Apis mellifera*—a species that has also been witnessed visiting *H. caprinum* and *H. jankae*. Unsurprisingly, compared with visitors to subgenus *Himantoglossum*, bees observed visiting subgenus *Barlia* flowers are on average larger bodied; they encompass at least six species of four genera, most commonly *Bombus* (Teschner, 1977; Claessens & Kleynen, 2011). Only *Bombus canariensis* has so far been seen visiting *H. metlesicsianum* (Teschner, 1993), though it seems to us unlikely that this bumble bee is the sole pollinator of this orchid. The two species of subgenus *Barlia* have similar average frequencies of fruit set (Claessens & Kleynen, 2011).

In summary, there is little evidence in *Himantoglossum s.l.* of the strong pollinator specificity that is all too frequently invoked (though often with negligible evidence) for other groups of European orchids (e.g., Sun, Gross & Schiestl, 2014). Any genuine differences between these orchids in pollinator spectra are more likely to reflect the geographic distributions of the potential pollinators than the functional morphology of the orchid flowers. In contrast, *Steveniella satyrioides* is suspected to operate a contrasting mode of food deceit, wherein social wasps transfer pollinaria as they reputedly seek insect prey within the proportionately substantial spur (Nazarov, 1995; Claessens & Kleynen, 2011).

### Contrasting distributions of the three subgenera among the Mediterranean islands

Recent fluctuations in the northwestern limit of *H. hircinum* (reviewed by Carey, 1999; Carey & Farrell, 2002; Pfeifer, Heinrich & Jetschke, 2006) suggest that its seeds can migrate and then germinate with comparative ease, and suitable pollinating insects are known to be present across the Mediterranean islands. However, despite this supposed long-distance mobility, the mainland distributions of the more widespread *Himantoglossum* species show remarkably little overlap and equally little disjunction (Fig. 5; see also Fig. 9B of Sramkó et al., 2014 and Fig. 1 of Biró & Bódis, 2015). Subgenus *Comperia* is severely constrained to Asia Minor, not occurring further west than the larger of the peri-Turkish Aegean islands: Samos, Lesvos, Chios and Rhodes (Petrou, Petrou & Giannakoulias, 2011). In contrast, both *H. robertianum* and the various members of subgenus *Himantoglossum* occur across most of the Mediterranean Basin (the former being absent only from the Levant and the latter only from northeast Africa).

However, the respective distributions of these two subgenera on the Mediterranean islands are strikingly different. No member of subgenus *Himantoglossum* has succeeded in establishing itself on the Balearics, Corsica, Sardinia, Malta/Gozo or Cyprus, yet all these islands support vigorous populations of *H. robertianum*. Subgenus *Himantoglossum* only reached Crete and Sicily (the latter a mere 3 km distant from the Calabrian mainland). This raises the question of whether *H. robertianum* could have survived on the Mediterranean islands since the catastrophic Zandean flood filled the Mediterranean Basin eastward from the Gibraltar arc within an estimated two-year period (Garcia-Castellanos et al., 2009) at *ca* 5.3 Ma, thereby abruptly ending the Messinian salinity crisis of *ca* (6.0–)5.6–5.3 Ma (Rouchy & Caruso, 2006).

Probably not. Although the Iberian and Cypriot accessions of *H. robertianum*—located at opposite ends of the Mediterranean—differ in both plastid and ITS sequences, the disparities between them are an order of magnitude less than the molecular divergence separating subgenus *Barlia* from subgenus *Himantoglossum* (Fig. 21). And that divergence was dated molecularly by Sramkó et al. (2014) to 5.7 ± 2.5 Ma—approximately the time of the Messinian crisis. Indeed, Sramkó et al. (2014) suggested that the xeromorphic features and precocious flowering of subgenus *Barlia* could have been adaptations to the arid climates that presumably characterised Messinian times.

One possible explanation for the absence of subgenus *Himantoglossum* from most Mediterranean islands would be if suitable mycorrhizal partners were similarly absent. Studies of the mycorrhizae of *H. adriaticum* (Pecoraro et al., 2013) and both species of subgenus *Barlia* (Liebel et al., 2010) identified generalist mycorrhizal associates that are widespread among European Orchidinae. However, it may be significant that although the mycorrhizae gave modest assistance to both species of subgenus *Barlia* when accumulating nitrogen, only the mycorrhizae of *H. robertianum* also contributed to carbon accumulation in the orchid (Liebel et al., 2010). Recent studies showing how germination frequency in seeds of species of *Anacamptis*, *Orchis* and* Gymnadenia* (genera at least fairly closely related to *Himantoglossum*: Bateman et al., 2003; Tang et al., 2015) decline rapidly within 5 m of the ‘mother’ plant vividly illustrate the challenges facing seeds that experience long-distance dispersal (Jacquemyn et al., 2012; McCormick & Jacquemyn, 2014).

In summary, although the potential for long-distance dispersal of seeds may indeed be great, the potential for successful establishment of those seeds appears to be much lower.

### Character evolution

#### Conceptual prologue

Cladistic representation of relationships between a pair of species as sisters rather than ancestor and descendant is a logical necessity if a hypothesis of relationship is to be derived from a data-matrix with maximum objectivity. Unfortunately, this approach also severely handicaps any attempt to reconstruct the morphologies of the ancestors that would have occupied the internal nodes of a cladogram (e.g., Bateman, Hilton & Rudall, 2006). Early attempts to address this problem often relied on ‘Brownian motion’ models. These models assumed that the (hypothetical) common ancestor of species A and species B possessed a phenotype that was in all parameters the precise average of the two descendant sister species. Thus, if species A possesses six white petals and species B possesses four red petals, their common ancestor is assumed to have possessed five pink petals! Unfortunately, logic tells us that their common ancestor almost certainly possessed either four red petals or six white petals, but we cannot be certain which of these two conditions actually pertained; the one fact of which we *can* be confident is that the ancestral species did *not* possess five pink petals!

Hence, more complex models of nodal reconstruction have since become predominant, based on various kinds of probability estimate. These models all depend to varying degrees on weight of numbers; if several successive nodes on the cladogram subtend species possessing six white petals, there is a high statistical probability that those intervening ancestors also possessed six white petals. It is important to realise that this logic does not necessarily mirror evolutionary reality; consider, for example, a single long-lived, widespread, evolutionarily conservative species that repeatedly gives rise to more localised species (e.g., Fig. 2 of Bateman, 2011), each of which possesses the alternative character state to that shown by the long-lived ancestor. This is no mere theoretical consideration; one incontrovertible example is provided by the numerous local autogamous species of the Eurasian orchid *Epipactis* that evolved independently from within a single geographically widespread allogam, *E. helleborine* (Squirrell et al., 2002). At best, reconstruction of internal nodes on a cladogram remains a case of building somewhat subjective scenarios, albeit within a framework of (hopefully) less subjective data.

#### Seeking the most credible outgroup for the *Himantoglossum* clade

In addition, the chosen outgroup(s) play a particularly important role in influencing the perceived set of character states hypothesised to have been possessed by the common ancestor of the entire ingroup, which by definition is located at the so-called root node. This issue is problematic in the case of the *Himantoglossum* clade (reviewed by Bateman, 2012a). Topological congruence at the genus level among well-sampled molecular phylogenetic studies of subtribe Orchidinae is limited to two relevant nodes, specifically agreement that: (1) *Neotinea s.l.* (2*n* = 42) is sister to a 2*n* = 36 clade consisting of the genera *Himantoglossum s.l.*, *Ophrys*, *Serapias* and *Anacamptis s.l*., and (2) *Serapias* and *Anacamptis s.l.* are sisters. Irrespective of whether they constructed trees using maximum parsimony, likelihood or Bayesian algorithms, all three published ITS-only studies yielded uncertain relationships among the five well-supported groups in the 2*n* = 36 clade: *Serapias* plus *Anacamptis s.l.*, *Ophrys*, *Himantoglossum s.l*., and *Steveniella* (Bateman et al., 2003; Sramkó et al., 2014; Tang et al., 2015). By adding to a subset of Bateman et al.’s ITS data a modest number of base-pairs derived from the plastid *rpl16* intron, Inda, Pimentel & Chase (2012) strengthened statistical support for a topology in which *Himantoglossum s.l.* was sister to *Serapias* plus *Anacamptis s.l.* and *Ophrys* was sister to all three genera. Unfortunately, this topology was obtained in the absence of *Steveniella*—a genus whose presence in a phylogenetic analysis of Orchidinae is crucial, as it was tentatively placed as sister to *Himantoglossum s.l.* in the molecular studies of Bateman et al. (2003) and Sramkó et al. (2014).

When seeking close relatives of *Himantoglossum s.l.* that might usefully inform speculation regarding the initial phenotype of this lineage, clearly both *Ophrys* and *Serapias* are too divergent (molecularly and morphologically) and too reproductively specialised to provide useful guidance. *Anacamptis s.l.* also appears unsuitable, given its reliably derived phylogenetic position as sister to *Serapias*. This understanding leaves *Steveniella* as the only credible extant candidate for the role of archetype of the *Himantoglossum* clade.

Eventual acquisition of next-generation sequencing data (e.g., Olson, Hughes & Cotton, 2016) across the 2*n* = 36 clade of subtribe Orchidinae clade should, in theory at least, help to resolve several outstanding issues in phylogeny reconstruction and taxon circumscription. They would provide a further test regarding whether the improbably early divergence of *H. formosum* suggested by our *LEAFY* tree (Fig. 20A) is, as we suspect, a misleading aberration (Sramkó et al., 2014), and could also provide a much-needed additional test of whether *Steveniella* is viewed correctly by us as sister genus to *Himantoglossum s.l.*

**Figure 23:**
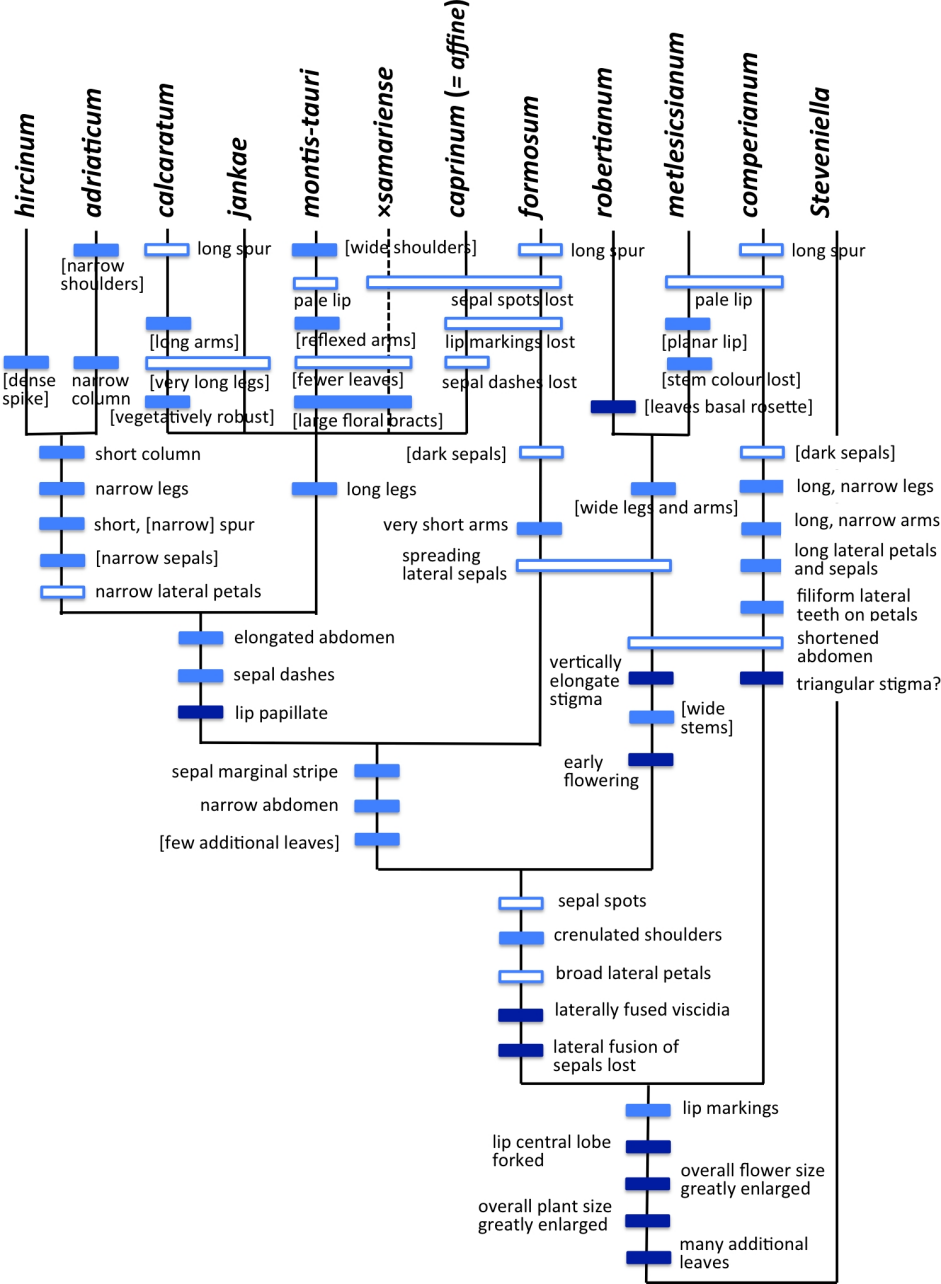
Summary of the inferred evolution of *Himantoglossum s.l.* Estimated positions of key morphological transitions identified during the present study, mapped across a framework molecular topology.

Having emphasised the assumptions that we felt obliged to make in pursuing this line of thought, we can now proceed to consider character evolution in *Himantoglossum s.l.* As described by Bateman et al. (2003, p. 16), “the smaller, 1–2-leaved, small-flowered *Steveniella* superficially resembles members of the *Neotinea s.l.* and the [dominantly East Asian: Tang et al., 2015] *Neottianthe∼Hemipilia* clade, though its gynostemium structure, purplish-brown galea, strongly three-lobed labellum and short, robust spur do—as Sprengel (1826) perceived—suggest similarities to the more derived himantoglossids”. Here, we further extend that earlier morphological comparison, placing *Steveniella* at the root of the evolutionary scenario that we have built upon our present morphometric matrix (Figs. 18 and 23).

#### Labellum shape

We will begin this part of the discussion by considering the ‘floral skeletons’ that we abstracted from our raw data in order to facilitate systematic comparison of labellum size and shape (Fig. 18).

*Steveniella* is, in shape and especially size, more typical of earlier-divergent groups of subtribe Orchidinae than of any member of *Himantoglossum s.l.* Admittedly, the labellum of *H. formosum* could in theory be generated simply by considerably expanding that of *Steveniella* and incising a small distal notch into its central lobe. This is an especially interesting observation in the light of the fact that these two species have the eastern-most distributions of any of the taxa under scrutiny (they actually co-exist in the Caucasus), and might therefore be predicted to be closely related. However, both ITS and plastid topologies refute any such closeness of relationship (Sramkó et al., 2014) (Fig. 20).

Labella of subgenus *Comperia* and subgenus *Barlia* were most likely generated from a *Steveniella*-like ancestor by lengthening all structures except the abdomen, which consequently became proportionately shorter, and substantially broadening the labellum (most notably in *H. metlesicsianum*). It is equally parsimonious for the comparatively short abdomen to have arisen in parallel in both lineages, or to have been acquired only once, before becoming greatly elongated during the origin of subgenus *Himantoglossum*. In subgenus *Barlia* the limbs and spur became short and robust, whereas in subgenus *Comperia* they became slender but greatly elongated (Fig. 18). This developmental trend for extreme elongation even affected the lateral teeth on the lateral petals, causing the teeth to become filiform. Following divergence of subgenus *Barlia*, the arms became very short and the abdomen became considerably narrower in *H. formosum*, thereby permitting development of the sinistral spiralling that characterises the remainder of subgenus *Himantoglossum*. The abdomen then became greatly elongated (and often more tightly spiralled) to generate the distinctive labellar bauplan of the *hircinum–caprinum* clade. Separation between section *caprinum* and section *hircinum* involved substantially increasing the length of the legs and slightly broadening the shoulders of the former (Fig. 18). In section *hircinum*, spur size was reduced back to that characteristic of *Steveniella* and legs became even narrower.

Although it is not clear from the molecular phylogenies which member of section *caprinum* originated first, *H. jankae* is most geographically widespread and most similar in labellar ratios to *H. hircinum* and *H. adriaticum*. Taking into account all the available evidence, it appears most likely that *H. jankae* gave rise to *H. calcaratum*, *H. montis-tauri* and *H. caprinum* comparatively recently; each most likely arose independently as a parapatric variant. The concomitant changes in flower dimensions were subtle; simple enlargement of the labellum in the case of *H. calcaratum* (abdomen and spur became especially large) but slight shortening of the labellum of *H. caprinum* and *H. montis-tauri* plus, in the case of the latter, widening of the labellum (Fig. 18).

#### Shapes of other flower parts

It is tempting to view changes in the length and/or width of the two lateral petals and especially of the three sepals as direct functional consequences of the changes documented in the shape and/or size of the third, median petal—the labellum—which must be wholly enclosed throughout the development of the bud (Fig. 22A). These perianth segments became elongated in *H. comperianum* to accommodate the filiform limbs of its distinctive labellum and broadened in the remaining species to encompass their more robust labella. The entire flower became more compact during the origin of section *hircinum*, the column also being shortened. Both the column and labellum narrowed further in *H. adriaticum* relative to *H. hircinum.*

The sepals of *Steveniella* and *H. comperianum* are laterally fused to about one-third of their length from their base (a feature also characteristic of early-divergent members of the related orchid genus *Anacamptis s.l.*: Bateman & Hollingsworth, 2004), but this feature was then lost prior to the origins of the remaining taxa of *Himantoglossum*. The lateral sepals were thus free to spread partially outward in subgenus *Barlia* and even more strongly outward in *H. formosum* (Fig. 11B), leaving only the median sepal and lateral petals to form the galea overarching the gynostemium. It is possible that spreading sepals had a single origin before the divergence of subgenus *Barlia*, followed by re-integration of the lateral sepals into the galea in the *hircinum–caprinum* clade. However, it seems more likely that spreading lateral sepals arose independently in the two lineages. Surprisingly, lateral fusion of the sepals was lost at the same point in the evolutionary history of the group as the viscidia became laterally fused (Fig. 23). Consequently, in the case of subgenera *Barlia* and *Himantoglossum*, both pollinaria must be removed by pollinators as a single physical unit.

#### Flower markings and colour

The *Himantoglossum* clade possesses a remarkable range of floral markings, but it is even more remarkable that each different category of markings appears to be inherited independently of the others. Labellar markings certainly originated at the evolutionary origin of *Himantoglossum* (and may have been inherited from its direct ancestor—although labellum markings are absent from *Steveniella*, species of the more closely related of the genera in the 2*n* = 42 clade, *Neotinea s.l.* and *Orchis s.s*., all routinely possess them). With regard to markings on the sepals of *Himantoglossum*, interior spots arose only after the divergence of *H. comperianum*, the marginal stripe only after the divergence of the *robertianum* group, and the interior dashes only after the separation of *H. formosum*, when the labellum markings also became raised on a central papillate region (Figs. 22C and 23) (Bateman et al., 2013). All markings other than sepal marginal stripes were subsequently lost independently from *H. formosum* and from *H. caprinum* (which also lost the papillae that are reliably correlated with labellum markings in the *hircinum* and *jankae* groups: Sramkó et al., 2012).

The base colour of the labellum appears to have become on average paler independently in *H. comperianum*, *H. metlesicsianum* and *H. montis-tauri*, whereas the reverse (abaxial) surface of the sepals darkened independently in *H. comperianum* and *H. formosum*. *Himantoglossum* offers particularly graphic examples of how combinations of contrasting pigments can generate unusual flower colours; the way in which these plants achieve such results merits more detailed investigation of both the nature of the pigments involved and the precise location within the anatomy of the flower where those pigments are concentrated.

#### Vegetative characters

It is striking how small a role vegetative features play in the evolutionary scenario summarised in Fig. 23. By far their most critical involvement was the remarkable increase in overall body size (and associated increase in leaf number) that must have occurred during the origin of the genus *Himantoglossum*, as no close relative of the *Himantoglossum* clade contains species capable of showing an equivalent degree of vegetive vigour (among subtribe Orchidinae, only the *Anacamptis palustris* group can rival *Himantoglossum* for average body size). Otherwise, we detected only a further, and more modest, increase in body size in *H. calcaratum*, and a further, and equally modest, increase in leaf number at the origin of subgenus *Himantoglossum*. This increase in average leaf number subsequently reversed during the origin of *H. montis-tauri* (and that of *H. ×samariense*), where it was apparently compensated for photosynthetically by increased size of the already foliose bracts. Otherwise, mention need be made only of the somewhat fleshier stems of subgenus *Barlia*, and of the comparatively condensed inflorescence of *H. hircinum* (Fig. 23).

### Evolutionary overview

The morphology of a terrestrial orchid ultimately reflects the interaction of its genome(s) with its particular environment (ecophenotypy), both operating within the constraint of the size/maturity of the plant—this in turn is at least partly a consequence of its age since germination (ontogeny *s.l*.). The ratio of putatively genetic versus putatively epigenetic variation in morphology differs greatly among morphometric studies. Although genetic factors typically dominate, epigenetic factors are capable of demonstrating at least an equivalent influence on phenotype in analyses where the taxonomic spectrum has been constrained and morphological differences are therefore comparatively subtle (e.g., Bateman & Denholm, 1989; Bateman et al., 2014). In such cases, vegetative characters tend to figure as strongly as floral characters in the resulting multivariate ordinations, though they typically incur larger coefficients of variation (Bateman & Rudall, 2011).

This scenario certainly does not apply in the case of *Himantoglossum s.l.* Members of the clade are probably best known for their exceptionally large and complex labella, but we have demonstrated here that flower shape and size only slightly exceeds flower pigmentation when dictating the main axes of variation within the genus. Moreover, in contrast with some other orchid groups (e.g., Bateman & Denholm, 1985; Gigord, Macnair & Smithson, 2001), trends in pigmentation characters reflect various kinds of discrete markings more strongly than general variation in the background colour of the perianth members. Nonetheless, comparison of morphometric data with molecular data has shown that the value of visually striking discrete anthocyanin markings has been exaggerated when circumscribing species in at least some other groups of Eurasian orchids (e.g., Bateman, 2011).

The *Himantoglossum* clade presents a fascinating panoply of evolutionary patterns and processes. Its labellum—large, and unusually complex in both shape and pigmentation—offers an excellent case-study in both the potential for phenotypic diversification but also the developmental and structural constraints that ultimately limit that diversification. For example, the complexity of the labellum is ultimately constrained by the (admittedly remarkably sophisticated and efficient) manner in which the labellum is packaged within the developing bud (Fig. 22A). The relative sizes and shapes of contrasting regions of the labellum offer a marvellous case-study in allometry and especially heterochrony within a single organ (most studies of heterochrony focus on evolutionary changes in the relative timing of developmental events *between* organs rather than within them). The fluctuations in the size and shape of the labellum also usefully indicate that the later-developing features of this organ—arms, legs and spur—are more subject to intrapopulation variation than are earlier-developing features. Such fine details of orchid flowers are conventionally studied under the prior assumption that they represent fine-tuned adaptation to pollinators. However, they more likely reflect developmental plasticity. This in turn reflects epigenetic, and at least partially ecophenotypic, influences on floral development that in most cases ultimately prove to be of little macroevolutionary consequence.

The multidimensionality of morphological variation within the clade permits a considerable degree of homoplasy that is sufficient to complicate any attempt to identify broad evolutionary trends. For example, the orchid family owes its origin to profound congenital fusion of the polliniferous and ovuliferous organs (e.g., Rudall, Perl & Bateman, 2013), a condition that persisted throughout its diversification into today’s estimated *ca* 20,000 species. Nonetheless, of the two cases of organ fusion evident within the clade—lateral fusion of the three sepals in *Steveniella* and subgenus *Comperianum*, lateral fusion of the viscidia in subgenus *Barlia* and subgenus *Himantoglossum*—both transitions occur on the same branch of the evolutionary tree but with opposite polarities. Specifically, just as basal fusion of the three sepals is lost, fusion of the two viscidia is acquired; moreover, this feature has been retained by all subsequent members of the lineage (Fig. 23).

Evolutionary reversals occur frequently in the dimensions of various regions of the labellum and in several pigmentation characters. Interestingly, the polarity of changes in pigmentation characters can be asymmetric; discrete markings are clearly acquired sequentially by the lineage—first labellum spots, then sepal spots, then the sepal marginal line, and finally sepal dashes (Fig. 23), whereas it is at least theoretically feasible for all of these categories of marking to be lost via a single genetic change, at least in circumstances where that change caused the plants to cease the manufacture of the defining anthocyanin pigments. The fact that each of these categories of marking can demonstrably be lost separately implies a surprisingly complex set of genetically-based control mechanisms.

Also evident are several examples of phenotypic shifts that, within the conceptual framework proposed by Scotland (2011), are most appropriately termed parallelism at the level of morphological change and as convergence at the level of underlying genetic (or possibly heritable epigenetic) change. The spreading lateral sepals of subgenus *Barlia* and *H. formosum* probably originated independently. Spur length is a notoriously evolutionarily and developmentally labile character (e.g., Bateman, Rudall & Moura, 2013), so it is not surprising that considerable lengthening of the spur evolved in three species: *H. comperianum*, *H. formosum* and *H. calcaratum*. *Himantoglossum formosum* and *H. caprinum* independently lost labellum markings. Labella of *H. comperianum*, *H. metlesicsianum* and *H. montis-tauri* independently became paler (i.e., more reflective), whereas in contrast, sepals darkened in both *H. comperianum* and *H. formosum*.

Overall, the *Himantoglossum* clade includes likely examples of an impressive range of evolutionary mechanisms.

## Conclusions

The *Himantoglossum* clade has proved to be a particularly good model system for evolutionary studies. It contains a modest and manageable (if as yet uncertain) number of species that encompass wide ranges of both molecular and morphological divergence, but also differ radically in the amount of divergence evident between sister groups (i.e., its constituent lineages are highly likely to have diverged at very different times in the history of the clade: Sramkó et al., 2014). It therefore offers a reasonable prospect of developing causal explanations for particular evolutionary events.

The pattern of divergence in the *Himantoglossum* clade is fractal, the larger divergences in both genotype and phenotype (or, in an alternative scenario, the greater extinction frequencies) occurring comparatively early in the evolutionary history of the group (Figs. 19 and 20). Its more recent evolutionary history has yielded only far more subtle differences in both phenotype and genotype, implying that at least some gene flow is ongoing among those taxa that emerged comparatively recently. However, differences documented by Sramkó et al. (2014) in both plastid and nuclear ITS sequences between the Iberian and Cypriot representatives of *H. robertianum*, and among the Turkish, Georgian and Azerbaijani accessions of *Steveniella satyrioides* (Fig. 20), suggest that significant taxonomic structure may in fact be present within these apparently highly distinct and cohesive species.

Even a cursory glance at Fig. 18 is sufficient to suggest that the evolution of labellum (including spur) size and shape in *Himantoglossum* has been a story of shifts in the degree of development of contrasting portions of the labellum; some are allometric (percentage change in the size of the structure is equal in all directions, thereby retaining the ancestral shape) but most are heterochronic, involving directional heterogeneity in size change and thus causing shape change (e.g., Gould, 1977; Bateman, 1994; Bateman, Rudall & Moura, 2013).

In contrast, pigmentation features of *Himantoglossum* flowers relate more to shifts in the expression of particular pigments (and possibly also epidermal textures) at contrasting locations on the flower, apparently controlled by unexpectedly complex genetic systems. Having said that, the correlated loss from *H. caprinum* of labellum markings and the associated papillae may represent paedomorphic heterochrony, as papillae are formed only late in the ontogeny of the flowers of subgenus *Himantoglossum* (Bateman et al., 2013). Meanwhile, the vegetative robustness that characterises the entire genus has undergone only comparatively trivial evolutionary changes since its origin, the main exception being the increased xeromorphy of subgenus *Barlia*.

Overall patterns of character change have been complex and multi-directional (Figs. 7–10 and 23), thereby challenging attempts to simplify that variation into broader trends. Character parallelism/convergence and loss have been frequent, and inter-organ fusion has also played a potentially significant role. The extent of gene-flow within subgenus *Himantoglossum* remains undesirably speculative, but good evidence exists of at least one case of presumed homoploid hybrid speciation (sensu Rieseberg, 1997; Mallet, 2007) in the form of *H.* ×*samariense*.

*Himantoglossum* populations inhabiting islands, both in the Mediterranean and the North Atlantic, are potentially evolutionarily informative regarding the geographic expansion of species—not only by their presence but also by their frequent absence. The sister-group relationship within subgenus *Barlia* of *H. robertianum* versus the Canarian endemic *H. metlesicsianum* would be of even greater interest if sufficient circumstantial evidence could be gathered to infer that one of these species resembled the ancestor of the other (Bateman, 2012b). Molecularly estimated dates of arrival of *Himantoglossum* populations on particular Mediterranean islands could also be highly informative (Sramkó et al., 2014). And given sufficiently dense sampling, population genetic data could elucidate post-glacial migration routes (cf. the preliminary phylogeographic studies of *H. hircinum* conducted by Pfeifer et al., 2009).

Although artificial crossing has been used repeatedly to estimate comparative levels of post-zygotic reproductive isolation in European orchids (e.g., Scopece et al., 2007), the phenotypes presented by the resulting plants (e.g., Fig. 21) have thus far escaped serious discussion. Such experiments actually represent a golden opportunity to explore patterns of heritability of particular phenotypic features, seeking evidence of dominance, over-expression and linkage, as well as possibly epigenetic influences—phenomena that our experiences suggest more often nudge the phenotypes of primary hybrids toward the phenotype of the ovule parent rather than that of the pollen parent (e.g., Bateman, Smith & Fay, 2008).

*Himantoglossum* also offers intriguing conundra within the field of developmental biology. For example, it would be interesting to discover whether the labellar ‘limbs’ emulate labellar spurs in expanding mainly through cell elongation rather than cell division (e.g., Mack & Davis, 2015). And the occurrence of only sinistral helical torsion (chirality) of the distal portion(s) of the labellum throughout the genus remains extraordinary, as most other examples of torsion observed in plants have been declared to be random with regard to the ‘handedness’ of the relevant helical structure (e.g., Schilthuizen & Gravendeel, 2012). Moreover, if these two features are compared, there emerges a stark contrast between the exceptional developmental flexibility evident in the length of the limbs versus the developmental conservatism that is epitomised by the unidirectional chirality of the abdomen.

Clearly, there remains much still to be learned about this genus. Relatively new technologies that would help to better circumscribe species and elucidate evolutionary mechanisms include next-generation sequencing, flow cytometry and evolutionary-developmental (epi)genetics. However, further progress will equally depend on detailed, distribution-wide field observations and sampling that together allow reciprocal illumination between the demographic levels of individual plants, local breeding populations and *bona fide* species. Some previous piecemeal studies have caused more confusion than enlightenment because of the lack of such reciprocity (Appendix 1). Nonetheless, even though it reflects detailed reciprocal exploration, our revised classification (Appendix 2) should be regarded as provisional pending further studies.

## Appendix 1: Taxonomic Review

Despite the fact that it is a genus containing relatively few *bona fide* species, *Himantoglossum s.l.* has become a veritable graveyard of piecemeal taxonomy and hence of extensive synonymy. It provides a classic example of why nomenclatural decisions should follow, rather than precede, the application to taxonomic problems of multiple analytical approaches. The establishment of the name *H. jankae* (Molnár et al., 2012; Sramkó et al., 2012) had some scientific justification, but subsequent recombinations of other epithets as infraspecific taxa of *H. jankae* (Kreutz, 2014; Ponert, 2014; Delforge, Verstichel & Breuer, 2015) were mere deskbound exercises in nomenclatural floor-sweeping. Occasional expressions of intense frustration at the ensuing lack of nomenclatural stability (e.g., Van Lent, 2015) are therefore understandable.

### One, two or three genera?

The inclusion of *Comperia* and *Barlia* within *Himantoglossum* advocated by Delforge (1999) and then supported molecularly by Bateman et al. (2003) has found favour with only a minority of authors of European orchid floras and monographs, who often claim to prefer the “traditional” classification. Such statements overlook the fact that the placement of *Barlia* at sectional rank within *Himantoglossum* was enacted by orchid taxonomic guru Rudolf Schlechter 81 years earlier (Schlechter, 1918) (Table 6). Prior to Delforge (1999), most authors recognised a monotypic genus *Comperia*, though occasionally, *C. comperiana* was instead assigned to *Orchis s.l.* (e.g., Camus & Camus, 1929). Also, from a purely nomenclatural viewpoint, Camus & Camus (1929) and Nelson (1968) preferred *Loroglossum* to *Himantoglossum*. Among the authors of classifications listed in Table 6, only Schlechter (1918) explored the use of ranks intermediate between genus and species.

**Table 6:** Comparison of classifications of *Himantoglossum s.l.* published during the last half-century.

Present study	First use of epithet	Govaerts (2016) [World checklist]	Sramkó et al. (2014)	Delforge (2006)	Kreutz (1998), Kreutz (2004), Kreutz (2006)	Wood (1983)	Baumann & Künkele (1982)	Sundermann (1980)	Moore (1980)^1^	Vermeulen (1977)	Sundermann (1973)	Nelson (1968)	Camus & Camus (1929)	Schlechter (1918)
*H. comperianum*	1829	Hco	Hco	Hco	Cco	NA/T	Cco	Cco	Cco	Cco	NA/T	NA/T	Oco	Cco^2^
*H. robertianum*	1806	Hro	Hro	Hro	Bro	NA/T	Bro	Bro	Bro	Bro	NA/T	Bro	Blo	Hlo
*H. metlesicsianum*	1982	Hme	Hme	Hme	Bme	NA/T			NA/G	NA/G	NA/T			
*H. formosum*	1813	Hfo	Hfo	Hfo	Hfo	Hfo	Hfo	Hfo	NA/G	NA/G	Hfo	Lfo	Lhi=fo	Hfo
*H. hircinum*	1753	Hhi	Hhi	Hhi	Hhi	Hhi	Hhi	Hhi	Hhi	Hhi	Hhi	Lhi	Lhi	Hhi
*H. adriaticum*	1978	Had	Had	Had	Had	Hhi=ad	Had	Hhi=ad						
*H. calcaratum*=*calcaratum*	1887	Hca	Hca	Hcp–ca^3^		Hhi=ca	Hca	Hhi=ca	Hhi=ca	Hhi=ca	Hhi=ca	Lca	Lhi=ca	Hcp–ca
*H. calcaratum*=*jankae*	2012	Hja	Hja	Hcp	Hcp	Hhi=cp	Hcp	Hhi=cp	Hhi=cp	Hhi=cp	Hhi=cp	Lhi–cp	Lhi=cp	Hcp
*H. calcaratum*–*robustiss*.	2006	Hja=rb	Hja–rb^3^											
—“*rumelicum*”^*^	2005	Hja=ru												
*H. caprinum*	1819^4^	Hcp	Hcp	Haf	Haf	Hhi=af	Haf	Hhi=af		Haf	Haf	Laf	Laf	Haf
—“*pseudocaprinum*”^*^	1983	Hhi–pc				Hhi=af–pc								
*H. galilaeum*=*galilaeum*^*^	2008	Hga	Hga		NA/G									
*H. galilaeum*=*levantinum*^*^	2005				NA/G									
*H. montis-tauri*	1997^5^	Hmt	Hmt	Hbo	Hmt								Lhi=bo	Hbo
*H.*×*samariense*	1989	Hsa	Hsa	Hsa	Hsa									

**Notes.**

Taxonomic abbreviations: Genera B *Barlia* C *Comperia* H *Himantoglossum* L *Loroglossum* O *Orchis*
Species/infraspecific taxa ad *adriaticum* af *affine* bo *bolleanum* ca *calcaratum* co *comperianum* cp *caprinum* fo *formosum* hi *hircinum* ga *galilaeum* ja *jankae* le *levantinum* lo *longibracteatum* me *metlesicsianum* mt *montis-tauri* pc *pseudocaprinum* rb *robustissimum* ro *robertianum* ru *rumelicum* sa *samariensis*

^*^Taxonomic assignment provisional, as taxon not included in our morphometric analysis; NA/T Not applicable, as taxon lay outside the taxonomic spectrum of the study; NA/G Not applicable, as taxon lay outside the geographic catchment of the study. ^1^Treatment of *Comperia* by Von Soó (1980). ^2^Taxon omitted from the formal classification but binomial used in the accompanying text. ^3^Varietal status implied rather than explicitly stated. ^4^The epithet *affine* was coined in 1882. ^5^The older epithet *bolleanum* was coined a century earlier, in 1898.

### Species or subspecies?

Table 6 and Appendix 2 also makes clear the reluctance of the majority of observers to view as full species the taxa within subgenus *Himantoglossum* that were traditionally named *caprinum* and *calcaratum*; they were more often treated as subspecies of *Himantoglossum hircinum*. In contrast, only Sundermann (1980) and Wood (1983) viewed as a subspecies the taxon traditionally named *affine*, while *formosum* was judged to merit species status by all authors other than Camus & Camus (1929). From Kreutz (1998) onward, it became *de rigueur* to view all widely accepted species of subgenus *Himantoglossum* as full species—to a large degree, this collective decision represents a reversion to views originally expressed by Schlechter (1918) (Table 6).

### Taxonomic and nomenclatural history within the *hircinum–adriaticum* clade

*Himantoglossum adriaticum* was first described by Baumann (1978) as a full species but was rapidly demoted to a subspecies of *H. hircinum* by Sundermann (1980) and Wood (1983). However, authors from Baumann & Künkele (1982) and Kreutz (1998) onward have consistently treated this taxon as a full species distinct from *H. hircinum* (Table 6).

Several other taxa were initially described as infraspecific taxa of *H. hircinum* but were later either elevated to full species status or progressively transferred to species within the *jankae–caprinum* clade (see below). Nonetheless, new taxa are still occasionally described within the *H. hircinum–adriaticum* group, the most recent being the late-flowering *H. hircinum* var. *aestivalis* (Kreutz & Steinfeld, 2013).

### Taxonomic and nomenclatural history within the *jankae–caprinum* clade

Almost simultaneously, two taxonomists decided to distinguish between northern and southern populations of *H. jankae* (then known as *H. caprinum*). Baumann & Lorenz (2005b) separated these spotted-lipped southern populations as *H. caprinum* subsp. *rumelicum* (later recombined as *H. jankae* subsp. *rumelicum* by Ponert, 2014, and as *H. jankae* var. *rumelicum* by Delforge, Verstichel & Breuer, 2015). This taxon was reputedly distinguished largely on the basis of a greater mean length of its labellum, especially the lateral lobes, even though it was clear from the three mounted flowers illustrated in Fig. 1 of Baumann & Lorenz (2005b) that plants within the same population from northeastern Greece varied greatly in these characters (see also Tsiftsis, 2016). A year later, Kreutz (2006) used similar characters to distinguish northerly populations of *H. caprinum* as *H. caprinum* subsp. *robustissimum*, based on a holotype from northwest Turkey. Although this taxon appears to us to most likely be a synonym of subsp. *rumelicum*, it was subsequently recombined by Kreutz (2014) as *H. jankae* subsp. *robustissimum*,** evidently with the intention of maintaining *robustissimum* in parallel with subsp. * rumelicum*.

Wood (1983) formally distinguished plants of *H. caprinum* (referred to as *H. affine* by Wood) from Iraq bearing comparatively long legs (*ca* 15 mm) as var. *pseudocaprinum*, but we note that our northern Turkish Küçüçkucur population bore legs that similarly averaged 15 mm (Table 1). Var. *pseudocaprinum* was later mis-attributed to *H. hircinum* by Govaerts (2016).

A similar history surrounds *H. montis-tauri*, which was described by Kreutz (1997) to encompass populations in southwest Turkey with red-spotted labella that are unusually broad with compatively long lateral lobes (see also Kreutz, 2004; Kreutz, 2006). Baumann & Baumann (2005) compared selected features of *H. montis-tauri* with those of *H. bolleanum*, their comparative table suggesting that the two taxa differed only in *bolleanum* having lateral labellum lobes that averaged twice the length of those of *montis-tauri*. Consequently, Baumann & Baumann viewed *montis-tauri* as a synonym of *bolleanum*. *Himantoglossum bolleanum* was described from southwest Turkey more than a century earlier as *Aceras bolleana* by Siehe & Haussknecht (in Dammer, 1898; a species later transferred to *Himantoglossum* by Schlechter, 1918), albeit on the basis of a mediocre lectotype of a specimen from southern Turkey. Baumann & Baumann (2005), in their Fig. 3, argued that it was unclear whether the plant bore labellum markings. Baumann & Lorenz (2005a) therefore established as a new combination *H. caprinum* (= our *H. jankae*) subsp. *bolleanum*—the first usage of either of the two epithets *bolleanum* and *montis-tauri* at subspecies level since Camus & Camus (1929) had treated *bolleanum* as a subspecies of *H. hircinum* (Table 6). In his detailed reply, Kreutz (2006) noted that the (regrettably brief) description accompanying the lectotype of *‘Aceras’ bolleanum* does clearly state that the “flowers are green and rosy red. No spots occur on the labellum” (Dammer, 1898, p. 365). Kreutz consequently argued that *bolleanum* does not deviate significantly in morphology from typical *H. caprinum*, and that the 1997 epithet *montis-tauri* should therefore stand.

A decade before Siehe & Haussnecht (in Dammer, 1898) described *‘Aceras’ bolleana*, Beck (1887) had first described the large-spurred species *‘Aceras’ calcarata*. This putative species was relegated to varietal status as *H. hircinum* var. *calcaratum* by Schlechter (1918), but a decade later he altered his opinion and restored its species status as *H. calcaratum* (Schlechter, 1927)—immediately before Camus & Camus (1929) and Von Soó (1929) employed the one rank for this taxon that had remained vacant, by recognising *H. hircinum* subsp. *calcaratum*. The rank of subspecies was also preferred by Baumann & Lorenz (2005a), who transferred the taxon to *H. ‘caprinum’* (= our *H. jankae*)—a combination subsequently recombined as *H. jankae* subsp. *calcaratum* by Kreutz (2014), before Delforge (in Delforge, Verstichel & Breuer, 2015) interceded to reduce the rank of *calcaratum* back to the varietal level that it had occupied a century before under Schlechter’s scheme. However, Gölz & Reinhard (1986) had previously rejected *calcaratum*, at least as a credible species, after their morphometric study had identified only a negligible difference between taxon means for *calcaratum* and those for ‘typical’ *H. jankae*, in contrast with the large morphological disparities clearly separating *H. jankae* from both *H. hircinum* and *H. adriaticum*. Unfortunately, the rank awarded to *calcaratum* is crucial from a nomenclatural viewpoint. Once Sramkó et al. (2012) had shown that the epithet *‘caprinum’* should actually be applied to the species formerly widely known as *H. affine*, Molnár et al. (2012) established the name *H. jankae* for the widespread, typically lip-marked species that extends eastward from Slovakia at least as far as northern Turkey. But if the more geographically restricted Jugoslavian endemic *calcaratum* is not viewed as being distinct from *H. jankae* at species level, the law of nomenclatural priority (ICBN Art. 11.3) would dictate that *calcaratum* should in turn replace the (consequently remarkably short-lived) epithet *jankae* (*contra* Kreutz, 2014; Delforge, Verstichel & Breuer, 2015).

Rückbrodt & Rückbrodt (1987) first drew taxonomic attention to an apparently atypical population of subgenus *Himantoglossum* in the Lefka Ori mountains of western Crete, argung that it was morphologically intermediate between, and combined features typical of, *H. jankae* and *H. caprinum*. They understandably hesitated to formally name the population, but this was done two years later by Alibertis & Alibertis (1989). Delforge (1999) subsequently suggested that both *H. samariense* and *H. montis-tauri* could actually be stabilised hybrids between *H. jankae* and *H. caprinum*. In the case of *H. ×samariense* at least, this hypothesis of hybridogeny received support from the molecular phylogenetic study of Sramkó et al. (2014).

Sramkó et al. (2014) also analysed molecularly a sample of a taxon morphologically intermediate between the *caprinum* and *jankae* groups that had been collected in northern Israel. This plant nested within the *jankae–montis-tauri* clade in the *LEAFY* tree, was placed immediately below the *jankae–montis-tauri* clade in the plastid tree, but in the ITS tree it was shown as derived due to possession of a single autapomorphic base (Fig. 20). Although they are weak and internally contradictory, these slight molecular differences from other Himantoglossums suggest that plants from Israel (and elsewhere in the Levant) could be subtly distinct. Certainly they would have made a useful addition to our morphometric analysis. Baumann & Baumann (2005) described Lebanese plants with unusually long labella (including lateral lobes) and red bursicles as *H. caprinum* (= our *jankae*) subsp. *levantinum*. Just three years later, Shifman (2008) described from Israel *H. galilaeum*, which he considered to be closer to our *H. caprinum* than to our *H. jankae*. However, the holotypes of *levantinum* and *galilaeum* appear to differ primarily in arm length—shown here to be an especially weak taxonomic character. Moreover, even Shifman’s (2008) own comparative table suggests that there is no meaningful morphological difference between *H. galilaeum* and typical *H. caprinum* other than an unusually even distribution across the labellum of diffuse pink-purple anthocyanins, while Kreutz (2006) suggested that *H. caprinum* subsp. *levantinum* differed from subsp. *caprinum* only in having somewhat larger, less horizontally oriented flowers.

Considered together, the examples outlined above make clear that several taxonomic uncertainties within the *jankae–caprinum* clade cannot be adequately resolved without a demographic approach being applied to the entire clade across its entire distribution, involving sampling that is sufficiently dense to distinguish geographic clines from the more abrupt congruent phenotypic and genotypic discontinuities that are the strongest evidence of species boundaries (Bateman, 2001; Bateman, 2012b). Any conclusions that we draw below from our own data should therefore be viewed as interim, awaiting studies that involve even more detailed sampling of both populations and characters.

### Taxonomic and nomenclatural history within subgenus *Barlia*

*Himantoglossum metlesicsianum* was first described as *Barlia metlesicsiana* by Teschner (1982), before being transferred to *Himantoglossum* by Delforge (1999). Unlike most European orchids that are only subtly morphologically distinct from their closest relative, the epithet *metlesicsianum* has never been used at any infraspecific level. Originally viewed as a comparatively high-altitude endemic confined to the ancient volcanic cores of the Canarian island of Tenerife (Stierli-Schneider, 2004; Kropf, Sommerkamp & Bernhardt, 2012), the orchid has since been found (typically at lower altitudes) in the north and west of the island (Claessens, 2015) and also in even smaller numbers on the nearby island of La Palma (Acevedo Rodriguez & Mesa Coello, 2013). It is of great conservation interest (Rankou, Fay & Bilz, 2011; Sramkó et al., 2014).

The degree of molecular divergence between *H. metlesicianum* and *H. robertianum* first demonstrated by Bateman et al. (2003) and then documented in greater detail by Sramkó et al. (2014) is in itself sufficient justification for accepting their status as separate species, irrespective of any morphological divergence. Although our whole-genus multivariate analyses (Figs. 7 and 9) suggest little morphological discrimination between these two species, a more nuanced comparison of taxon mean values for individual characters reveals several significant differences. Compared with *H. robertianum*, labella of *H. metlesicsianum* are on average *ca* 28% larger in linear dimensions (Fig. 16), much of the additional length reflecting a longer neck. This consequently has more sloping shoulders (Fig. 18) grading into broader arms; these occupy the same plane as the torso rather than being somewhat recurved. The leaves of *H. metlesicsianum* are on average 20% more numerous and 30% narrower, giving them a more lanceolate appearance. This impression is further enhanced by the fact that the leaves are often evenly distributed along the stem and decline gradually in size toward the inflorescence, whereas those of *H. robertianum* tend to be concentrated into a basal rosette. Also, the inflorescence averages one-third of the total height of *H. metlesicsianum* but almost half the total height of *H. robertianum*. In terms of pigmentation, our study plants of *H. metlesicsianum* reliably lacked stem pigmentation (but see Fig. 1.3 of Claessens, 2015) and had labella with much paler margins (Fig. 15A) that resemble the sepals in colour, as they lack the greenish-brown marginal pigments that characterise *H. robertianum*.

### Circumscription and ranks suggested by the present study

Applying the phylogenetic classification criteria of Bateman (2012a) makes clear that the three former genera encompassed by today’s more broadly circumscribed genus *Himantoglossum* definitely merit retention as subgenera on both molecular and morphological grounds; thus, subgenus *Comperia*, subgenus *Barlia* and subgenus *Himantoglossum*. Within subgenus *Himantoglossum*, again both molecular and morphological data support recognition of three formal taxonomic sections: *Formosum*, *Hircinum* and *Caprinum*.

Considering now the all-important species level, no evidence has accumulated to suggest that subgenus *Comperia* contains more than one species. Our molecular and morphological results support the segregation of the Canary Island populations of subgenus *Barlia* as a species separate from the far more widespread *H. robertianum*. And within subgenus *Himantoglossum*, the earliest-divergent species *H. formosum* clearly differs substantially from the remaining species in molecular, morphological and biogeographic characteristics. These taxonomic decisions are thus unambiguous, whatever the ongoing nomenclatural uncertainties

However, within the remainder of subgenus *Himantoglossum*, distinguishing species from named taxa more appropriately treated as infraspecific becomes a more complex, and in some cases speculative, challenge. A conservative approach that required reliable divergence in both morphological and molecular characteristics would recognise only two species, based on the *hircinum–adriaticum* clade (for which the name *H. hircinum* has priority) and the *jankae–caprinum* clade (for which the name *H. caprinum* (Von Bieberstein, 1819) Sprengel (1826) would have priority over *H. calcaratum* Beck (1887)). If more subtle but nonetheless consistent levels of molecular, morphological and geographic distinction are accepted, *H. adriaticum* unequivocally merits segregation from *H. hircinum*, due primarily to its reliable distinction on both the plastid phylogeny (Fig. 20B) and the multivariate analyses (Figs. 8A and 10). In this context, every aspect of the biology of *H. adriaticum* will soon be the subject of a separate, detailed review (J Bódis et al., unpublished data). The remaining challenge then becomes determining whether the level of distinction evident between *H. hircinum* and *H. adriaticum* has any parallel within the *jankae–caprinum* clade.

Setting aside the nothospecies *H.* ×*samariense*, which apparently had multiple origins via hybridity, the taxonomic consensus (Table 6) would suggest that the primary division within the *jankae–caprinum* clade should separate *H. caprinum* (formerly *H. affine*) on the one hand from *H. jankae* (formerly *H. caprinum*), *H. calcaratum* and arguably also *H. montis-tauri* on the other. However, our data suggest that there exists a case that is arguably stronger for distinguishing the southwest Turkish endemic *H. montis-tauri* from the remainder. This taxon is monophyletic and distinct—albeit only subtly—in both the *LEAFY* and ITS trees (Fig. 20), seemingly refuting any hypothesis of hybridogenesis that might reasonably be inferred from its unusually extensive morphological variation evident especially in pigmentation characters (cf. Figs. 7A vs 7B; also Fig. 11A). Moreover, the taxon appears to be both cohesive and distinct when viewed at the population level (Figs. 9 and 10), being held together by characters such as its exceptionally pale labella (Fig. 15A) and high width-to-length ratio of its labellum (Figs. 16A and 18). If pigmentation characters are ignored, *H. montis-tauri* resembles *H. caprinum* more closely than the *H. jankae* group at an individual level (Fig. 8B) but the *H. jankae* group more closely than *H. caprinum* at a population level (Fig. 10B); both these taxa have distributions which overlap with that of *H. montis-tauri* in Turkey (Fig. 5). *Himantoglossum montis-tauri* may owe its subtle distinctiveness to comparative isolation that reflects the high elevation of its headquarters in the Taurus Mountains.

Character weighting becomes a more crucial issue when comparing *H. caprinum* (formerly *H. affine*) with the *H. jankae* group (including *calcaratum* and *robustissimum*). In classical taxonomy, *H. caprinum* was viewed as a separate distinct species even when both *jankae* and *calcaratum* were viewed as subspecies of *H. hircinum* (Sundermann, 1973; Vermeulen, 1977; Wood, 1983) (Table 6). Although several authors have argued that *H. caprinum* is easily distinguished using morphological characters such as a pale unmarked labellum and short labellar arms, none of the genic regions analysed by us allowed reliable distinction (Fig. 20). And comparison of our ordinations that include all scored characters with those omitting pigmentation characters show that the apparent distinction between *H. caprinum* and *H. jankae s.l.* relies heavily—arguably too heavily—on floral markings (Figs. 8, 10 and 11). Moreover, our data clearly show that neither depth of flower colour (Fig. 15A) nor arm length (Fig. 17A) actually allows consistent separation of the two taxa.

Among the named taxa analysed morphometrically by us, only the supposed Balkan endemic *H. calcaratum* and supposed North Turkish endemic *H. jankae robustissimum* remain to be discussed. Our Turkish population of *H. jankae*, which represented subsp. *robustissimum*, was reliably identical to samples of *H. jankae* from eastern Europe for all three genic regions sequenced (Fig. 20). Other than its substantially greater spur length (Fig. 15B) and arm length (Fig. 17A), *robustissimum* overlaps strongly with *H. calcaratum* in both the multivariate ordinations (Figs. 8 and 10) and the plots of individual characters (Figs. 12–18); it also resembles *H. calcaratum* in mean labellum shape except in possessing a length of abdomen closer to that of *H. jankae s.s.* (Fig. 18). In addition, the two taxa exhibit similar frequences of the various kinds of flower markings (Fig. 11A). Thus, *robustissimum* presents a morphology that is in most characteristics intermediate between that of *H. jankae s.s.* and that of *H. calcaratum*, but overall is closer to the latter. If *calcaratum* is henceforth to be treated as a subspecies of *H. jankae*, this implies that *robustissimum* should be treated as a variety rather than a subspecies (*contra* Kreutz, 2006). Given the substantial geographic disjunction that separates the Jugoslavian *calcaratum* from the north Turkish *robustissimum*, it would be most appropriate to simply attribute var. *robustissimum* to *H. jankae s.l*.

As shown by Table 6, those previous authors who regarded *H. jankae* as a subspecies of *H. hircinum* also regarded *H. calcaratum* as a subspecies of *H. hircinum*. More recently, both Delforge (1999; see also Delforge, 2006) and Kreutz (2004) synonymised *calcaratum* into *H. jankae*, though *calcaratum* has persisted as a full species in the World Checklist (Govaerts, 2016). Our data show that, when ordinated at the level of individual plants, *calcaratum* largely associates with *jankae robustissimum*. And when analysed at the population level, *calcaratum* reliable connects with *jankae s.s.* or, less frequently, with *jankae robustissimum* (Figs. 8 and 9). As already noted, the only characters offering clear distinction of *calcaratum* from the other named taxa in the *jankae–caprinum* clade is its long (10–14 mm) spur, supported to a lesser degree by long labellar arms (10–20 mm) and an unsually long torso (Fig. 18). When this phenotype is considered alongside its highly restricted geographical distribution, and the fact that these features decrease in size clinally outwards from Bosnia-Herzegovina (J Bódis, pers. comm., 2015), these observations suggest that *calcaratum* is best treated as a subspecies of *H. jankae*. A similar conclusion was reached, on the basis of a less ambitious morphometric analysis confined to floral dimensions, by Gölz & Reinhard (1986).

This conclusion has particularly unfortunate implications for nomenclature within the *jankae–caprinum* group. It was only since 2012 that we were able to demonstrate that the taxon previously named at species level *H. affine* should in fact be named *H. caprinum*, and that the taxon previously know as *H. caprinum* should therefore be awarded a newly coined epithet, *jankae*. However, if it is accepted that *calcaratum* is best treated a subspecies of *H. jankae*, as suggested above, the epithet *jankae* (Molnár et al., 2012) then becomes a synonym of *calcaratum* (Beck, 1887) at species level. This outcome is demanded by the law of nomenclatural priority, despite the fact that *calcaratum* is not an accurate descriptor of the vast majority of the populations within *H. jankae*, which have spurs that are merely average in size when considered across subgenus *Himantoglossum* as a whole. And priority also undermines the epithet *‘jankae’* after a mere four years of its existence, at a time when the epithet was becoming more widely accepted by the orchidological community (e.g., Kreutz, 2014; Ponert, 2014; Delforge, Verstichel & Breuer, 2015; Govaerts, 2016).

The most notable taxon not yet discussed, which we analysed genetically (Sramkó et al., 2014) but not morphometrically, is the putative Levant endemic *H. galilaeum* (cf. Baumann & Baumann, 2005; Kreutz, 2006; Shifman, 2008). Although both the ITS and plastid trees show modest deviation from genotypes typical of the *H. jankae–caprinum* clade, comparative morphological-taxonomic tables for this taxon indicate that it combines characteristics typical of *H. caprinum* with those more typical of *H. montis-tauri* and *H. jankae* (especially *‘robustissimum’*). On the basis of the available (albeit inadequate) evidence, we suspect that *galilaeum* and *levantinum* are actually both morphologically similar and closely related to each other, being in turn more closely related to the *H. caprinum* group than to the *H. calcaratum/jankae* group. In the absence of either morphometric or molecular data we have provisionally treated *levantinum* as a subspecies of *H. galilaeum* in Appendix 2 and Table 6, though credible cases could be made for either downgrading *levantinum* to varietal status or treating both *galilaeum* and *levantinum* as infraspecific taxa within *H. caprinum* (as was recently suggested by Vela & Viglione, 2015). There is evidently a particularly urgent need for more detailed and broadly based investigations of populations of subgenus *Himantoglossum* occurring in the Levant.

The net result of these multi-faceted deliberations is the revised classification presented in the Introduction of the present work as Fig. 4 (previous classifications are summarised as Table 6).

### Taxonomic postscript

No formal taxonomic (re)descriptions are attempted here in support of the above classification. Although generating quantitative descriptions, combining morphological and molecular characters, would be feasible based on our accumulated data (e.g., Bateman, Rudall & Moura, 2013), experience has taught us that morphometrically analysing a minimum of 100 plants representing ten populations per putative species is desirable in order to generate an acceptably accurate formal taxonomic description. Nonetheless, we have accrued sufficient data to be confident that previous formal descriptions, particularly within subgenus *Himantoglossum*, are rich in inaccuracies.

In this context, we learned only when completing the present manuscript that Tsiftsis (2016) had recently conducted a well-sampled morphometric survey of 363 plants from 24 populations of the *H. jankae* group—a group that is widely distributed in mainland Greece, together with the more taxonomically controversial populations of the Peloponnese and Aegean (as represented by Lesvos). The resulting data were subjected to rigorous statistical analyses but were confined to only 11 metric characters, most describing dimensions of perianth members: sepals (4), petals (2), labellum plus spur (4), and ovary (1). Having acquired these measurements, Tsiftsis (2016) challenged the conclusion reached by Sramkó et al. (2012), on the basis of measurements taken from 28 herbarium specimens, that *H. jankae s.l.* and *H. caprinum s.l.* (formerly *H. affine*) could be reliably distinguished on the grounds that *H. jankae* has longer spurs and much longer lateral lobes of the labellum (‘arms’). The present data offer some support Tsiftsis’ (2016) arguments: spur lengths are 5.1 ± 0.9 mm for *H. jankae s.s.* (these figures are unchanged if *‘robustissimum’* is included) versus 6.2 ± 1.0 mm in *H. caprinum*, and arm lengths are 5.9 ± 1.9 mm in *H. jankae* (mean rising slightly to 7.0 ± 1.9 mm if *‘robustissimum’* is included) versus 4.4 ± 1.6 mm in *H. caprinum*. Thus, the differences between the two taxa in these characteristics are too subtle to achieve acceptable levels of statistical significance.

The incongruence between the present results, obtained from *in situ* populations, and those derived from herbarium material by Sramkó et al. (2012) supports previous assertions that obtaining metric data from herbarium specimens rather than living plants is hazardous. They are rarely sufficiently numerous, and often are insufficiently representative of their source populations. Moreover, unless the measured organs are isolated and glued flat when still fully hydrated, dehydration can greatly reduce organ size and often also alters organ shape when shrinkage is anisotropic (cf. Bateman & Rudall, 2006; Bateman, Rudall & Moura, 2013; Parnell et al., 2013). In addition, it becomes abundantly clear that arm (i.e., lateral labellum lobe) and leg (i.e., secondary lobules of central labellum lobe) lengths—much vaunted as key diagnostic characters among taxa of subgenus *Himantoglossum*—routinely incur exceptionally large coefficients of variation within populations and are, in truth, among the least valuable characters for taxonomic use within subgenus *Himantoglossum*.

## Appendix 2: Revised Classification of *Himantoglossum s.l.*

This revised classification is based on integration of our morphometric and molecular data. An asterisk indicates taxa whose status is provisional, pending more detailed studies, and the list excludes several trivial varieties/forms, notably those of *H. hircinum*. We note that, given the appalling nomenclatural complexity inherent in this (and many other) group of orchids, it is unlikely that this classification is itself entirely free of errors.

**Table:**

Genus *Himantoglossum* Spreng., *Syst. Ve*g. 3, 675, 694. 1826, emend. W.D.Koch, *Syn. Fl. Germ. Helv.* 689, 841. 1837 [nom. cons.].
Synonyms: *Loroglossum* L.C.Rich., *Mém. Mus. Paris* 4: 41, 47. 1918 [nom. illegit.].
*Aceras* R.Br. subgenus *Calcaratae* Kränzlin, *Gen. Sp. Orch*.: 165: 1901.

Subgenus *Comperia* (C.Koch) R.M.Bateman, Molnár & Sramkó,** stat. nov**.
Type (here designated): *Himantoglossum comperianum* (Steven) P.Delforge.
Synonym: Genus *Comperia* C.Koch, *Linnaea* 22: 287. 1849.

*Himantoglossum comperianum* (Steven) P.Delforge, *Natural. Belges* 80(3): 401. 1999.
Basionym: *Orchis comperiana* Steven, *Nouv. Mém. Soc. Imp. Natural. Moscou* 1, 8: 259. 1829.
Synonyms: *Comperia taurica* C.Koch, *Linnaea* 22: 288. 1849, nom. illeg.
*Comperia comperiana* (Steven) Ascherson & Gräbner, *Syn. Mitteleur. Fl*. 3(5): 620. 1907.
*Comperia karduchorum* Bornmüller & Kränzlin, *Bull. Herb. Boissier* 3: 141. 1895.

Subgenus *Barlia* (Parlatore) Orchidaceae Himantoglossum subgen. *Barlia* (Parl.) R.M. Bateman, Molnár & Sramk.
Type: *Himantoglossum robertianum* (Loisel.) P.Delforge.
Synonym: Genus *Barlia* Parlatore, *Fl. Ital.* 3: 445. 1854.

*Himantoglossum robertianum* (Loisel.) P.Delforge, *Natural. Belges* 80: 401. 1999.
*Barlia longibracteata* Parl., *Nuov. Gen. Spec. Mon. eoest*: 5. 1854.
*Loroglossum longebracteatum* Moris, in *Ard. Fl. Alp. Mar*.: 351. 1867.
*Himantoglossum longibracteatum* (Biv.) Schltr., *Die Orchid*.: 52. 1914.
*Barlia robertiana* (Loisel.) Greuter, *Boissiera* 13: 192. 1967.

*Himantoglossum metlesiscianum* (W.P.Teschner) P.Delforge, *Natural. Belges* 80: 401. 1999.
Basionym: *Barlia metlesicsiana* W.P.Teschner, *Orchidee* (Hamburg) 33(3): 117. 1982.

Subgenus *Himantoglossum*.
Synonym: Subgenus *Euhimantoglossum* Schlechter, *Feddes Repert. sp. nov*., 285. 1918. [illegitimate under ICN Article 21.3].

Section *Formosum* R.M.Bateman, Molnár & Sramkó,** sect. nov**.
Type (here designated): *Himantoglossum formosum* (Stev.) C.Koch.
**Section diagnosis:** Labellar lateral lobes (‘arms’) less than 3 mm, labellar ‘abdomen’ less than 20 mm, labellar spur greater than 4 mm; lateral sepals spreading rather than connivent into hood; gynostemium greater than 4.5 mm.
ITS sequence contains the following motif within ITS2: T**A**AACCCAACTCAATATTCAAT**TCAGT**AGAAG (i.e., includes the SNP A and the five-base insertion TCAGT; *trnL*(UAG)*–rpl32* IGS plastid sequence contains the following motif: AGAACCTCATT**CACTTTATT**CAC (i.e., includes the nine-base insertion CACTTTATT; *rpl32–ndhF* IGS plastid sequence contains the following motifs: AT**TCAAAATTTAT**ATTGAATTCGTGAGATTCTT**C**ATCA (i.e., includes the 11-base sequence TCAAAATTTAT and SNP C) and AAAAGC**C**CAATG**G**TGATAT (i.e., includes the SNPs C and G).

*Himantoglossum formosum* (Stev.) C.Koch, *Linnaea* 22: 287. 1849.
Basionym: *Orchis formosa* Stev., in *Mém. Soc. Nat. Mosc.* 4: 66. 1813.
Synonyms*: Aceras formosa* (Stev.) Lindl., *Gen. Spec. Orch Pl.* 1: 282. 1835.
*Loroglossum formosum* (Stev.) E.G.Camus, Bergon & A.Camus, *Monogr. Orch. Eur*.: 83. 1908.
*Loroglossum hircinum* L.C.Rich. subsp*. formosum* (Stev.) E.G.Camus & A.Camus, *Iconogr. Orch. Eur*. 1: 123. 1928.

Section *Hircinum* R.M.Bateman, Molnár & Sramkó,** sect. nov**.
Type (here designated): *Himantoglossum hircinum* (L.) Spreng.
**Section diagnosis:** Labellar lateral lobes (‘arms’) greater than 3 mm, labellar ‘abdomen’ greater than 20 mm, labellar spur less than 4 mm; lateral sepals connivent into hood rather than spreading; gynostemium less than 4.5 mm.
ITS sequence contains the following motif within ITS2: T**T**AACCCAACTCAATATTCAATAGAAG (i.e., excludes the SNP A and the five-base insertion TCAGT); *trnL*(UAG)*–rpl32* IGS plastid sequence contains the following motif: AGAACCTCATTCAC (i.e., excludes the nine-base insertion CACTTTATT; *rpl32–ndhF* IGS plastid sequence contains the following motifs: AT**TCAAAATTTAT**ATTGAATTCGTGAGATTCTT**C**ATCA (i.e., includes the 11-base sequence TCAAAATTTAT and SNP C) and AAAAGC**A**CAATG**A**TGATAT (i.e., includes the SNPs A and A).

*Himantoglossum hircinum* (L.) Spreng., *Syst. Veg.* 3: 694. 1826.
Synonyms: *Satryium hircinum* L., *Spec. Pl*.: 944. 1753.
*Orchis hircina* Crantz, *Strip. austr*.: 484. 1769.
*Loroglossum hircinum* (L.) L.C.Rich., *Mém. Mus. Paris* 4: 54. 1818.
*Aceras hircina* (L.) Lindley, *Gen. Sp. Orch*.: 282. 1835.

*Himantoglossum adriaticum* H.Baumann, *Orchidee* (Hamburg) 29(4): 171. 1978.
Synonym: *Himantoglossum hircinum* (L.) Spreng. subsp. *adriaticum* (H.Baumann) H.Sund., *Eur. Medit. Orc*h. ed. 3: 40. 1980.

Section *Caprinum* R.M.Bateman, Molnár & Sramkó,** sect. nov**.
Type (here designated): *Himantoglossum caprinum* Spreng.
**Section diagnosis:** Labellar lateral lobes (‘arms’) greater than 3 mm, labellar ‘abdomen’ greater than 20 mm, labellar spur greater than 4 mm; lateral sepals connivent into hood rather than spreading; gynostemium greater than 4.5 mm.
ITS sequence contains the following motif within ITS2: T**T**AACCCAACTCAATATTCAATAGAAG (i.e., excludes the SNP A and the five-base insertion TCAGT); *trnL*(UAG)*–rpl32* IGS plastid sequence contains the following motif: AGAACCTCATTCAC (i.e., excludes the nine-base insertionCACTTTATT); *rpl32–ndhF* IGS plastid sequence contains the following motifs: ATATTGAATTCGTGAGATTCTT**A**ATCA (i.e., lacks the 11-base deleted sequence TCAAAATTTAT and possesses SNP A) and AAAAGC**A**CAATG**A**TGATAT (i.e., includes the SNPs A and A).

*Himantoglossum calcaratum* (G.Beck) Schltr., *Repert. Spec. Nov. Regni Veg*. A1: 145. 1927.
(formerly *H. caprinum* auct mult., *H. jankae* Somlyay, Kreutz & Óvári)
Basionym: *Aceras calcarata* G.Beck, *Ann. Naturhist. Hofmus. Wien* 2: 55. 1887.
Synonyms*: Loroglossum calcarata* Beck (G.Beck) G.Beck, *Ann. Naturhist. Hofmus. Wien* 5: 576. 1890.
*Himantoglossum hircinum* (L.) Spreng. var. *calcaratum* (G.Beck) Asch. & Graebn., *Syn. Mittel. Fl*. 3: 787. 1907.
*Himantoglossum hircinum* (L.) Spreng. var. *calcaratum* (G.Beck) Schltr., *Repert. Spec. Nov. Regni Veg*. 15: 287. 1918.
*Loroglossum hircinum* L.C.Rich. var. *calcaratum* Janchen, Österr. *Bot. Zeit.* 68: 338. 1919.
*Himantoglossum calcaratum* (G.Beck) Schltr., *Repert. Spec. Nov. Regni Veg*. 1: 145. 1927.
*Himantoglossum hircinum* (L.) Spreng. subsp. *calcaratum* (G.Beck) Soó, *Bot. Arch*. 23: 90. 1929/1928.
*Himantoglossum caprinum* (M.Bieb.) Spreng. subsp. *calcaratum* (G.Beck) H.Baumann & R.Lorenz, *J. Eur. Orch.* 37: 716. 2005.

Subsp. *calcaratum**
Synonyms: *Himantoglossum jankae* Somlyay, Kreutz & Óvári subsp. *calcaratum* (G.Beck) Kreutz, *Ber. Arbeitskr. Heim. Orchid.* 31: 119. 2014 [publ. 2015, nom. superfl.].
*Himantoglossum jankae* Somlyay, Kreutz & Óvári var. *calcaratum* (G.Beck) Delforge, *Nat. Belges* 96: 16. 2015.

Subsp. *jankae* (Somlyay, Kreutz & Óvári) Bateman, Molnár & Sramkó, **comb. nov.**
Basionym: *Himantoglossum jankae* Somlyay, Kreutz & Óvári, *Phytotaxa* 73: 9. 2012.

Var. *robustissimum* (Kreutz) Bateman, Molnár & Sramkó, **comb. nov.**
Basionym: *Himantoglossum caprinum* (M.Bieb.) Spreng. subsp. *robustissimum* Kreutz, *J. Eur. Orch.* 38: 122. 2006.
Synonyms: *Himantoglossum jankae* Somlyay, Kreutz & Óvári subsp. *robustissimum* (Kreutz) Kreutz, *Ber. Arbeitskr. Heim. Orchid*. 31: 119. 2014 [publ. 2015].
*Himantoglossum jankae* Somlyay, Kreutz & Óvári subsp. *rumelicum* (H.Baumann & R.Lorenz) J.Ponert, *J. Eur. Orch*. 46: 563. 2014.
*Himantoglossum jankae* Somlyay, Kreutz & Óvári var. *rumelicum* (H.Baumann & R.Lorenz) P.Delforge, Verstichel & Breuer, *Nat. Belges* 96: PAGE. 2015.

*Himantoglossum caprinum* Spreng., *Syst. Veg*. 3: 694. 1826.
(formerly *H. affine* (Boiss.) Schltr.)
*Aceras affinis* Boiss., *Fl. Orient*. 5(1): 56. 1884/82.
*Loroglossum affine* E.G.Camus, Bergon & A.Camus, *Monogr. Orch. Eur*.: 83. 1908.
*Himantoglossum affine* (Boiss.) Schltr., *Repert. Spec. Nov. Regni Veg*. 15: 287. 1918.
*Himantoglossum hircinum* (L.) Spreng. subsp. *affine* (Boiss.) H.Sund., *Eur. Medit, Orch*. ed. 3: 40. 1980.
*?Himantoglossum hircinum* (L.) Spreng. subsp. *affine* (Boiss.) H.Sund. var. *pseudocaprinum* J.J.Wood, *Kew Bull*. 38: 75. 1983.

Subsp. *caprinum*.
Basionym: *Orchis caprina* M.Bieb., *Fl. Taur. Cauc.* 3: 602. 1819.
Synonyms: *Aceras caprina* Lindl., *Gen. et Spec. Orch*.: 282. 1825.
*Loroglossum caprinum* Beck, in *Ann. Nat. Hofm. Wien V*: 571. 1890.
*Himantoglossum hircinum* (L.) Spreng. subsp. *caprinum* (M.Bieb.) K.Richt., *Pl. Eur*. 1: 276. 1890.
*Himantoglossum hircinum* (L.) Spreng. var. *caprinum* (M.Bieb.) W.Zimm., *Allg. Bot. Z. Syst*. 23: 11. 1917.

Subsp. *?levantinum** (B.Baumann & H.Baumann) Kreutz, *Eurorchis* 17: 106. 2005/2006.
Synonyms: *Himantoglossum caprinum* subsp. *levantinum* B.Baumann & H.Baumann, *J. Eur Orch*. 37: 256. 2005. [No holotype].
*?Himantoglossum galilaeum* Shifman, *J. Eur. Orc*h. 40: 731. 2008.

*Himantoglossum montis-tauri** Kreutz & W.Lüders, *J. Eur. Orch*. 29: 655. 1997.
Synonyms: *Aceras bolleana* Siehe, *Gard. Chron*. 1: 265. 1898.
*Himantoglossum bolleanum* (Siehe) Schltr., *Rep. Spec. Nov. Reg. Veg*. 15: 287. 1918.
*Himantoglossum caprinum* (M.Bieb.) Spreng. subsp. *levantinum* H.Baumann & R.Lorenz, *J. Eur. Orch*. 37: 258. 2005.
*Himantoglossum caprinum* (M.Bieb.) Spreng. subsp. *bolleanum* (Siehe) H.Baumann & R.Lorenz, *J. Eur. Orc*h. 37: 716. 2005.

*Himantoglossum* nothosp. *×samariense* C.Alibertis & A.Alibertis, *Orchidophile* 20(87): 110. 1989.
*(=?H. caprinum*×*calcaratum*).
*Himantoglossum affine* (Boiss.) Schltr. subsp. *samariense* (C.Alibertis & A.Alibertis) H.Baumann & R.Lorenz, *J. Eur. Orch*. 37: 713. 2005.

## Appendix 3: Morphometric Characters Measured

Numbers of characters measured in the field are asterisked. Those that were excluded from the multivariate analyses—because they proved to be unmeasurable (C51), were measured inconsistently (C10), were scored too late for inclusion in the analysis (C39), duplicated another character (C13) or proved to be invariant (C14, C20)—are placed in brackets.

### A. Stem and inflorescence (8 characters, all used)

Note that floral bract length and ovary length were measured on the sampled floret subsequently mounted for characterisation of floral organs.

1.* Stem height, above ground level (including inflorescence).
2.* Stem diameter, above uppermost sheathing leaf.
3.* Anthocyanin pigmentation on stem immediately below inflorescence, on a scale 0–2 (0 = none, 1 = diffuse, 2 = dense).
4.* Inflorescence length.
5.* Number of flowers plus buds.
6.* Bract length, basal.
7. Bract length, floral.
8. Ovary length.

### B. Leaves (6 characters, 3 used)

The leaves of *Himantoglossum s.l.* often wither immediately before or during flowering (especially in desiccating habitats), thereby preventing accurate measurement. They can also be difficult to categorise in the genus. By definition, basal leaves form a spreading rosette immediately above ground level, whereas cauline leaves arise from the stem above its base, and are usually significantly smaller than any of the basal leaves. Unfortunately, some leaves that appear basal early in the annual growth cycle of a plant later appear cauline as the stem elongates. In order to minimise operator-specific inconsistencies, leaf counts for C9 and C10 were amalgamated prior to analysis, together forming C9a. Wholly omitted characters were width of widest leaf (C13), which usually equated with width of longest leaf (C12), and shape of longest leaf (C14), which in *Himantoglossum* proved to score uniformly as state 1 (i.e., no measured plant possessed oblanceolate rather than lanceolate leaves).

[9a.]* Total number of leaves (basal plus cauline).
[9.]* Number of basal leaves.
[10.]* Number of cauline leaves.
11.* Length of longest leaf.
12.* Width of longest leaf.
[13.]* Width of widest leaf [omitted, as value often equalled that of C12].
[14.]* Shape of longest leaf, as determined by the position of maximum width relative to length, on a scale 1–2 (1 = <50%; 2 = >50%).

### C. Labellum (19 characters, 18 used)

Coding of colours via the standard Commission Internationale de l’Éclairage coordinates is well-illustrated in Figs. 12 and 13. The anthropomorphic terminology used to describe contrasting regions of the labellum is illustrated in Fig. 6. Broadly, ‘arms’ are the ‘lateral lobes’ of most previous authors, ‘abdomen’ is their ‘mid-lobe’, and ‘legs’ are their ‘lobules’. The ‘torso’ consists of the abdomen plus the ‘thorax’. The potential landmarks that separate these regions are termed ‘neck’ (= spur entrance), ‘armpit’ and ‘crotch’, respectively. ‘Shoulder’ width was measured because the variable posture of the ‘arms’ precluded accurate measurement of overall labellum width.

15. Width across ‘shoulders’.
16. Minimum width of ‘torso’.
17. Maximum length of labellum.
18. Overall length of ‘torso’ (= ‘thorax’ plus ‘abdomen’).
19. Length of ‘thorax’, from spur entrance to ‘armpit’.
[20.] Spiralling of ‘abdomen’, on a scale 0–2 (0 = absent; 1 = dextral; 2 = sinistral). This character was omitted from analysis, as in those taxa that possess labellar torsion the chirality proved to be uniformly sinistral.
21. Number of crenulations (if present) along left ‘shoulder’.
22. Length of ‘arm’, from apex to ‘armpit’.
23. Width of ‘arm’, measured halfway along length.
24. Length of ‘leg’, from apex to ‘crotch’.
25. Width of ‘leg’, measured halfway along length.
26. Length of ‘tail’ [in the population-level analysis, this was converted to C26a: absence (0) or presence (1) of tail].
27. Colour of ‘limbs’, x (arbitrary values potentially ranging from 0–740).
28. Colour of ‘limbs’, y (arbitrary values potentially ranging from 0–830).
29. Colour of ‘limbs’, percentage reflectivity (Y, ranging from 3–89%).
30. Number of dark pigmented spots on ‘torso’ [in the population-level analysis, this was converted to C30a: absence (0) or presence (1) of spots].
31. Distribution of spots across ‘torso’ (if present), on a scale 0–3 (0 = absent; 1 = concentrated immediately below spur entrance, through to; 3 = distributed across most of ‘torso’).
32.* Attitude of ‘torso’ relative to stem, on a scale 1–3 (1 = parallel; 2 = diagonal; 3 = perpendicular).
33.* Attitude of ‘arms’ relative to ‘torso’, on a scale 1–4 (1 = shallowly decurved; 2 = planar; 3 = shallowly recurved; 4 = strongly recurved).

### D. Spur (3 characters, all used)

34. Length, from entrance to apex.
35. Diameter, halfway along length when viewed laterally.
36. Curvature, on a scale 1–5 (1 = strongly recurved, through to 5 = strongly decurved).

### E. Lateral petals (2 characters, both used)

37. Length.
38. Maximum width (measured immediately distal to any lateral teeth present).
39. Presence (1) or absence (0) of a single tooth on both petal margins (in order to qualify as a tooth, the apex of the distal margin of the lateral projection must subtend an angle of less than 90^∘^ relative to the distal margin of the main blade of the petal) [this character was omitted from the multivariate analyses as it was scored after they had been completed].

### F. Lateral sepals (9 characters, all used)

40. Length.
41. Maximum width.
42. Base colour of external surface, x.
43. Base colour of external surface, y.
44. Base colour of external surface, percentage reflectivity (Y).
45. Presence (1) or absence (0) of continuous peripheral linear stripe on external surface.
46. Presence (1) or absence (0) of multiple linear markings on internal surface, alnged parallel to the venation.
47. Presence (1) or absence (0) of dispersed dots and/or dashes on internal surface.
48. Position of lateral sepals, on a scale 1–3 (1 = connivent with lateral petals and median sepal to form a hood; 2 = somewhat separated from other perianth segments; 3 = strongly spreading laterally in a wing-like posture).

### G. Column (3 characters, 2 used)

49. Length.
50. Maximum width.
[51.] Distance separating the centres of the two viscidia [this character was omitted from the multivariate analyses, as in practice it proved unmeasurable for most taxa].

Selected characters are later used to calculate the following 12 ratios, which summarise the shapes of certain vegetative and floral structures. The characters are numbered according to the above list and preceded by the letter ‘C’:

a. Robustness of stem. C2/(C1 + C2).
b. Percentage of stem bearing flowers. (100 × C4)/C1.
c. Density of inflorescence (flowers/cm). C5/C4.
d. Length of ovary relative to length of floral bract. C8/(C8 + C7).
e. Length of spur relative to length of ovary. C34/(C34 + C7).
f. Shape of longest leaf. C12/(C11 + C12).
g. Width of ‘torso’ relative to width of ‘shoulders’. C16/(C15 + C16).
h. Length of ‘arms’ relative to length of ‘torso’. C22/(C18 + C22).
i. Length of ‘legs’ relative to length of ‘torso’. C24/(C18 + C24).
j. Length of ‘arms’ relative to length of ‘legs’. C22/(C24 + C22).
k. Width of ‘arms’ relative to length of ‘arms’. C23/(C22 + C23).
l. Width of ‘legs’ relative to length of ‘legs’. C25/(C24 + C25).

## Additional Information and Declarations

### Field Study Permissions

The following information was supplied relating to field study approvals (i.e., approving body and any reference numbers): Collections were made under UK permit NE662 and Hungarian permits from the Hortobagy National Park Directorate (45–2/2000, 250–2/2001), Middle-Danube Environmental and Nature Conservation Authority (19642), and Trans-Tisza Environmental, Nature Conservation and Hydrological Authority (60547).

### Data Availability

The following information was supplied regarding data availability: The novel data are included in Table 1.

### Life Science Identifiers (LSIDs)

77159056-1 Orchidaceae *Himantoglossum* sect. *Caprinum* R.M.Bateman, Molnár & Sramkó 77159052-1 Orchidaceae *Himantoglossum* subgen. *Comperia* (K.Koch) R.M.Bateman, Molnár & Sramkó Orchidaceae *Himantoglossum* subgen. *Barlia* (Parl.) R.M.Bateman, Molnár & Sramkó 77159054-1 Orchidaceae *Himantoglossum* sect. *Formosum* R.M.Bateman, Molnár & Sramkó 77159055-1 Orchidaceae *Himantoglossum* sect. *Hircinum* R.M.Bateman, Molnár & Sramkó 77159057-1 Orchidaceae *Himantoglossum caprinum* subsp. *jankae* (Somlyay, Kreutz & Óvári) R.M.Bateman, Molnár & Sramkó 77159060-1 Orchidaceae *Himantoglossum caprinum* var. *robustissimum* (Kreutz) R.M.Bateman, Molnár & Sramkó
